# Shared and specific competing endogenous RNAs network mining in four digestive system tumors

**DOI:** 10.1016/j.csbj.2024.11.005

**Published:** 2024-11-05

**Authors:** Yulai Tang, Aamir Fahira, Siying Lin, Yiming Shao, Zunnan Huang

**Affiliations:** a Key Laboratory of Computer-Aided Drug Design of Dongguan City, The First Dongguan Affiliated Hospital, School of Pharmacy, Guangdong Medical University, Dongguan 523710, China; b Key Laboratory of Big Data Mining and Precision Drug Design of Guangdong Medical University, Key Laboratory for Research and Development of Natural Drugs of Guangdong Province, School of Pharmacy, Guangdong Medical University, Dongguan 523808, China; c Dongguan Key Laboratory of Sepsis Translational Medicine, The First Dongguan Affiliated Hospital, Guangdong Medical University, Dongguan 523710

**Keywords:** Digestive system tumor, Competing endogenous RNA, Prognosis, Cross-cancer analysis

## Abstract

**Background:**

Digestive system malignancies, including esophageal carcinoma (ESCA), stomach adenocarcinoma (STAD), liver hepatocellular carcinoma (LIHC), and colon adenocarcinoma (COAD), pose significant global health challenges. Identifying shared and distinct regulatory mechanisms across these cancers can lead to improved therapies. This study aims to construct and compare competing endogenous RNA (ceRNA) networks across ESCA, STAD, LIHC, and COAD to identify RNA biomarkers that could serve as precision therapeutic targets to enhance clinical outcomes and advance personalized cancer care.

**Methods:**

Clinical and transcriptomic data from The Cancer Genome Atlas (TCGA) were analyzed to predict differentially expressed RNAs using the edgeR package. The ceRNA networks were constructed using the miRcode and ENCORI databases. Functional enrichment analysis and prognostic RNA screening were performed with ConsensusPathDB and univariate Cox regression analysis.

**Results:**

we identified 6, 88, 55, and 41 RNA biomarkers in ESCA, STAD, LIHC, and COAD, respectively. Network analysis revealed shared and specific elements, with shared nodes enriched in cell cycle and mitotic processes. Several biomarkers, including HMGB3 and RGS16 (ESCA), COL4A1 and COL6A3 (STAD), CDCA5 and CDCA8 (LIHC), and LIMK1 and OSBPL3 (COAD), were consistent with prior studies, while novel biomarkers, such as C3P1 (ESCA), P2RY6 (STAD), and N4BP2L1 and PPP1R3B (LIHC), were discovered. Based on RNA correlation analysis, 1, 23, and 2 potential ceRNA regulatory axes were identified in STAD (PVT1/miR-490-3p/HMGA2), LIHC (DLX6-AS1/miR-139-5p/TOP2A, etc.), and COAD (STRCP1 & LINC00488/miR-142-3p/GAB1), respectively.

**Conclusions:**

This study advances the understanding of ceRNA networks in digestive cancers, highlighting RNA biomarkers with potential as therapeutic targets for personalized treatment strategies.

## Highlights

• Distinct and shared differentially expressed lncRNAs, miRNAs, and mRNAs identified across four digestive cancers.
• ceRNA networks revealed shared, semi-shared, and specific lncRNA-miRNA-mRNA interactions for ESCA, STAD, LIHC, and COAD.
• Prognostic analysis identified key RNA nodes with significant prognostic value across ESCA, STAD, LIHC, and COAD.
• Semi-shared ceRNA axes are more prevalent than shared or specific axes across digestive system tumors.
• Potential biomarkers include N4BP2L1 and PPP1R3B in LIHC, P2RY6 in STAD, and C3P1 in ESCA suggest precision medicine avenues.

## Graphical Abstract

**Figure:**
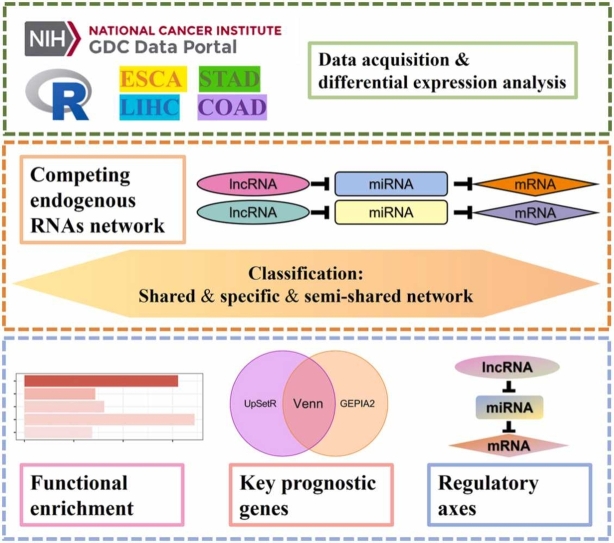

## 1 Introduction

Digestive tumors, including esophageal carcinoma (ESCA), stomach adenocarcinoma (STAD), liver hepatocellular carcinoma (LIHC), and colon adenocarcinoma (COAD), are significant contributors to global health challenges due to their complex nature and impact on patient outcomes [1]. Recent advancements in molecular medicine research have significantly enhanced our understanding of tumors, offering valuable insights into their discovery, diagnosis, and clinical treatment [2], [3]. Nevertheless, challenges persist in the screening, diagnosis, treatment, and prognosis of patients with digestive tumors, making it generally challenging. Recent bioinformatics studies have highlighted key functional gene sets and RNA network nodes that show potential as prognostic biomarkers for various digestive tumors [4], [5]. Therefore, identifying novel molecular targets associated with the prognosis of digestive system cancers, investigating their regulatory mechanisms, and developing potential biomarkers are crucial for improving patient treatment. The exploration of the molecular aspects of tumors extends beyond focusing on a single type. A shared RNA-mediated pathogenic process may be prevalent across numerous cancer types. Examining a diverse array of tumors helps identify both shared and distinct genetic traits among various cancer types [6]. For example, Chatziantoniou et al. identified key shared genes and their transcription regulators in adenocarcinoma and squamous cell carcinoma through a comprehensive analysis of multiple datasets [7]. Similarly, Salifu et al. identified novel therapeutic targets for hematological malignancies by conducting a comparative analysis of RNA-seq data from four common types of these cancers [8].

Competing endogenous RNA (ceRNA) network analysis is a major focus in cancer research, especially for understanding molecular mechanisms. Additionally, comparing the ceRNA of different cancers provides valuable insights through comparative analysis. The concept of the ceRNA network links protein-coding genes with noncoding RNAs (ncRNAs), such as microRNAs (miRNAs), and long noncoding RNAs (lncRNAs): lncRNAs interactively bind to miRNAs, which regulate mRNA levels. This interaction can disrupt mRNA expression and impact the occurrence and development of cancer [9]. While certain ceRNA networks may be shared across tumors within the same classification system, RNA networks involving the same dysregulated genes can exhibit variability depending on the specific tumor context [10], [11]. Zhao et al. demonstrated the involvement of the MIR17HG/miR-138-5p/HK1 axis in colorectal cancer liver metastasis [12]. Huang et al. demonstrated that the IGF2-AS/miR-503/SHOX2 axis promotes the progression and metastasis of gastric adenocarcinoma [13]. These studies suggest that shared ceRNA networks are clinically significant in cancer biology for identifying biomarkers, understanding cancer mechanisms, and predicting therapeutic responses. Furthermore, similar studies have strengthened our prospects for the exploration of tumor-shared dysregulation networks. By leveraging extensive sample data from public databases, establishing a ceRNA network, and conducting RNA node analysis, this comparative approach aims to elucidate RNA-mediated mechanisms underlying shared and specific disease biology, providing insights into novel prognostic biomarkers and advancing our understanding of RNA-mediated molecular mechanisms in cancer.

Initially, we acquired data and analyzed differential expression to identify lncRNAs, miRNAs, and mRNAs differently expressed in four digestive system cancers (ESCA, STAD, LIHC, and COAD). Furthermore, based on a comparison of these cancers, we classified genes into “shared”, “specific”, and “semi-shared” categories. This was followed by functional enrichment analysis and ceRNA network construction. Univariate Cox regression analysis and differential expression re-screening using GEPIA2 identified key mRNA nodes. Finally, potential ceRNA regulatory axes were identified by RNA correlation analysis. By constructing and comparing “shared”, “specific”, and “semi-shared” ceRNA networks across four digestive system tumors, we revealed potential RNA-mediated mechanisms underlying both shared and specific disease biology. This approach led to the identification of novel potential targets tailored to specific cancer subsets and the discovery of ceRNA regulatory axes associated with oncogenic processes. These findings provide valuable insights for tumor biomarkers discovery and advance our understanding of RNA-mediated molecular mechanisms in cancer research.

## 2 Materials and methods

### 2.1 Data acquisition and differential expression analysis

The dataset for esophageal carcinoma (ESCA), stomach adenocarcinoma (STAD), liver hepatocellular carcinoma (LIHC), and colon adenocarcinoma (COAD) before February 24, 2023, was obtained from the TCGA database (https://portal.gdc.cancer.gov/). The dataset comprised information on four key aspects: mRNA sequencing, miRNA sequencing, lncRNA sequencing, and clinical characteristics of the samples. The study adhered to the regulations of the TCGA public database, and all the data are publicly available without requiring additional consent from local ethics committees. Furthermore, Utilizing the “edgeR” package in R software (version R 4.3.1, https://www.r-project.org/), we conducted a differential expression analysis for lncRNA, miRNA, and mRNA in each digestive system tumor. Genes with an absolute log2 fold change value (|log2FC|) greater than 1 and an adjusted *P*-value (*P*-adj) less than 0.05 were considered significantly differentially expressed.

### 2.2 Construction of the lncRNA-miRNA-mRNA network

According to the concept of the competing endogenous RNA network, mRNAs and lncRNAs exhibit similar expression patterns. Conversely, miRNAs show expression patterns that are inversely related to both lncRNAs and mRNAs. These guidelines apply to the overlap of differentially expressed RNA (DE-RNA) and predicted RNA (pre-RNA) sourced from the ENCORI and miRcode databases. The lncRNA-miRNA pairs were identified using the miRcode database (http://www.mircode.org/) [14], and the pairing data for “highly conserved microRNA families” were downloaded. The miRNA-mRNA pairs were identified from the ENCORI database (https://rnasysu.com/encori/index.php) [15], applying the criterion that pairs must be present in at least two of the seven databases (Microt, Mirmap, Miranda, Pictar, Pita, RNA22, and Targetscan), and have a minimum CLIP-Data value of one. Cytoscape software (v3.9.1, https://cytoscape.org/) was used to visualize the RNA network.

### 2.3 Functional enrichment analysis

The ConsensusPathDB database (http://cpdb.molgen.mpg.de/CPDB) was used to conduct the pathway enrichment study [16]. The analysis included all pathway databases, with a set minimum overlap of 2 with the input list and a *P*-value cutoff of 0.01. The results were visualized using the Hiplot website (https://hiplot.cn/) [17].

### 2.4 Screening key prognostic genes

The “survival” package was employed to conduct univariate Cox regression analysis. Genes with a *P*-value < 0.05 were considered to have significant prognostic value, indicating their potential relevance in predicting outcomes. RNA nodes with significant prognostic value were classified and visualized using the “UpSetR” R package (https://github.com/hms-dbmi/UpSetR). The screening criteria for key prognostic mRNAs were defined as follows: (1) mRNAs with high expression in tumor patients, where high expression correlates with poor prognosis, were classified as potential oncogenes (red section). Similarly, mRNAs with low expression in tumor patients, where reduced expression is also linked to poor prognosis, were identified as potential tumor suppressor genes (green section). (2) Significant expression differences were observed in the merged data from TCGA and GTEx provided by the GEPIA2 database (http://gepia2.cancer-pku.cn/#index) [18].

### 2.5 Correlation analysis of prognostic RNAs in the ceRNA network

The correlations among prognostic RNAs within the ceRNA network were analyzed using the ENCORI database. Correlation coefficients for lncRNA/mRNA-miRNA pairs were calculated using the “miRNA-Target CoExpression” function while those for lncRNA-mRNA pairs were computed using the “RNA-RNA CoExpression” function. Based on these correlations, we identified the ceRNA regulatory axes and assessed their statistical significance, considering a *P*-value < 0.05.

## 3 Results

### 3.1 The workflow for mining shared and specific CeRNA networks in four digestive system cancers

**Fig. 1:**
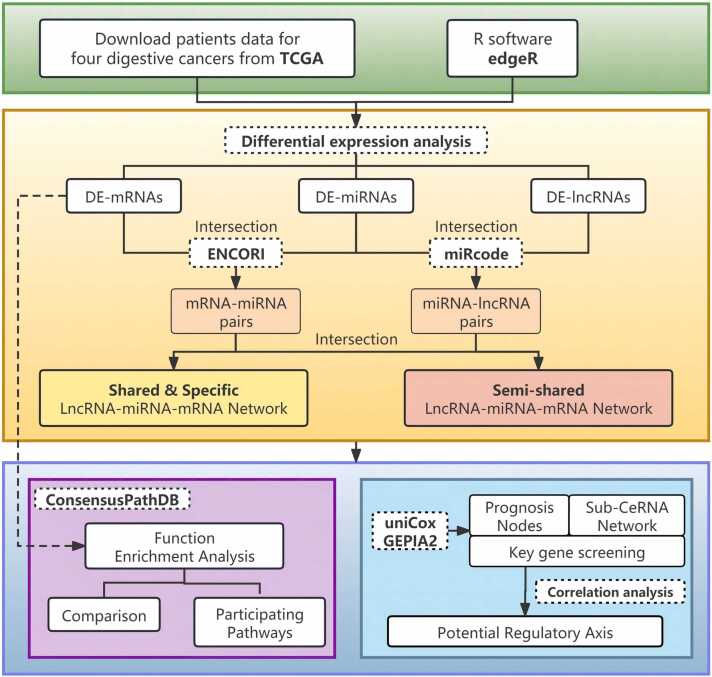
The workflow of this study. The diagram contains specific bioinformatics approaches and data mining tools.

Fig. 1 illustrates the workflow of constructing ceRNA networks, screening biomarkers, and identifying ceRNA regulatory axes in tumors by categorizing them as “shared”, “semi-shared”, or “specific” among four digestive system cancers. Concisely, RNA sequencing and clinical data for four digestive tumors were obtained from the TCGA database. The “edgeR” package was employed to identify differentially expressed lncRNAs (DElncRNAs), miRNAs (DEmiRNAs), and mRNAs (DEmRNAs). DE-RNAs (differentially expressed RNAs) were categorized as “shared”, “specific”, or “semi-shared” through comparison across digestive tumors. Functional enrichment analysis of the DEmRNAs was performed using the ConsensusPathDB database to unravel the shared and specific carcinogenic mechanisms underlying these four digestive tumors. The miRNA-lncRNA and miRNA-mRNA pairs were identified using miRcode and ENCORI databases, respectively. The lncRNA-miRNA-mRNA networks were subsequently visualized using Cytoscape software, revealing shared and specific RNA-mediated carcinogenesis mechanisms across the four digestive tumors. Through Univariate Cox regression analysis, we obtained the prognosis-related RNAs (DE-RNAs-os). Furthermore, upregulated genes with poor prognostic and downregulated genes with good prognostic were re-screened in the GEPIA2 database and then interested to remove inconsistent genes. This process identified key prognostic genes characterized by high expression levels associated with poor prognostic outcomes, as well as genes with low expression levels associations with good prognostic outcomes across four digestive tumors. Further analysis revealed related sub-ceRNA networks associated with these prognostic genes. Finally, conducting RNA correlation analysis through the ENCORI database enabled the identification of ceRNA regulatory axes influencing cancer progression.

### 3.2 Identification of differentially expressed RNAs in digestive system tumors

**Fig. 2:**
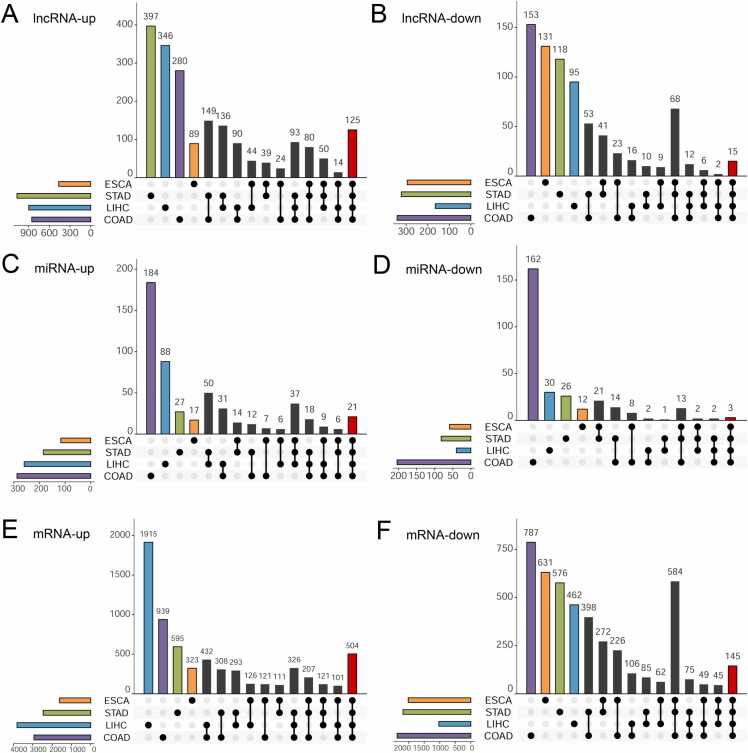
Identification of differentially expressed RNAs in digestive system tumors. (**A-B**): UpSetR plot of up-regulated / down-regulated lncRNAs in the four digestive tumors; (**C-D**): UpSetR plot of up-regulated / down-regulated miRNAs; (**E-F**): UpSetR plot of up-regulated / down-regulated mRNAs. **Annotation**: In each sub-graph, rows represented the sets of differentially expressed RNAs (DE-RNAs) in each of, between, or among four digestive system tumors, and columns represented the number of DE-RNAs in each category.

The differential expression analysis results revealed both distinct and shared patterns across various digestive tumors. Concisely, ESCA exhibited tumor-specific 760 DElncRNAs, 176 DEmiRNAs, and 3628 DEmRNAs. STAD displayed 1392 tumor-specific DElncRNAs, 267 DEmiRNAs, and 4645 DEmRNAs. LIHC demonstrated 1063 tumor-specific DElncRNAs, 300 DEmiRNAs, and 4847 DEmRNAs. COAD showcased 1197 tumor-specific DElncRNAs, 492 DEmiRNAs, and 5308 DEmRNAs (Table 1). Through intersection analysis, each cancer type exhibited dysregulation of specific genes. Concisely, In ESCA, 220 DElncRNAs were identified (89 upregulated, 131 downregulated). STAD showed 515 dysregulated DElncRNAs (397 upregulated, 118 downregulated), LIHC had 441 (346 upregulated, 95 downregulated), and COAD had 433 DElncRNAs (280 upregulated, 153 downregulated) (Fig. 2**A-B**). For DEmiRNAs, ESCA had 29 DEmiRNAs (17 upregulated, 12 downregulated), STAD had 53 (27 upregulated, 26 downregulated), LIHC had 118 (88 upregulated, 30 downregulated), and COAD had 346 (184 upregulated, 162 downregulated) (Fig. 2**C-D**). Furthermore, regarding DEmRNAs, ESCA had 954 DEmRNAs (323 upregulated, 631 downregulated), STAD had 1171 (595 upregulated, 576 downregulated), LIHC had 2377 (1915 upregulated, 462 downregulated), and COAD had 1726 (939 upregulated, 787 downregulated) (Fig. 2**E-F**). These dysregulations are specific to each type of digestive tumor, indicating distinct expression patterns in each. Additionally, a common set of dysregulated genes was identified across the four digestive system tumors. Concisely, there were 140 shared DElncRNAs, with 125 upregulated and 15 downregulated. Additionally, there were 24 shared DEmiRNAs, including 21 upregulated and 3 downregulated, and 649 shared DEmRNAs, with 504 upregulated and 145 downregulated. These shared DE-RNAs are represented by the red bar in Fig. 2**A-F**.

**Table 1:** Summary of the differentially expressed RNAs.

Cancer type	DE-lncRNA			DE-miRNA			DE-mRNA		
	Up	Down	Total	Up	Down	Total	Up	Down	Total
ESCA	465	295	760	117	59	176	1614	2014	3628
STAD	1069	323	1392	185	82	267	2465	2180	4645
LIHC	898	165	1063	260	40	300	3818	1029	4847
COAD	855	342	1197	288	204	492	2938	2370	5308

### 3.3 Functional enrichment analysis

**Fig. 3:**
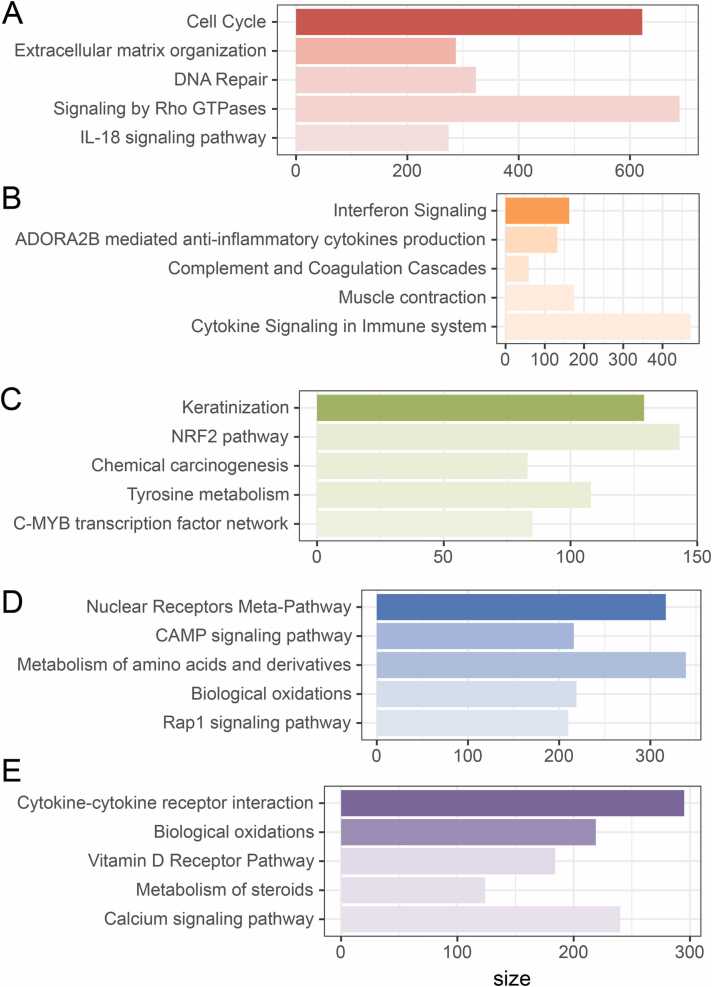
Functional enrichment analysis. (**A**): Functional annotation of shared mRNA network nodes; (**B-E**): Functional annotation of ESCA, STAD, LIHC, and COAD-specific mRNA network nodes, respectively.

Fig. 3 depicts the functional enrichment analysis of shared and specific mRNA nodes conducted by ConsensusPathDB. The results show that the shared mRNA nodes were predominantly enriched in the pathways related to cell cycle, extracellular matrix organization, DNA Repair, signaling by rho GTPases, and IL-18 signaling (Fig. 3**A**). The ESCA-specific mRNA nodes were mainly enriched in interferon signaling, ADORA2B-mediated anti-inflammatory cytokines production, complement, and coagulation cascades, Muscle contraction, and cytokine signaling in the immune system (Fig. 3**B**). The STAD-specific mRNA nodes were mainly enriched in keratinization, the NRF2 pathway, chemical carcinogenesis, tyrosine metabolism, and the C-MYB transcription factor network (Fig. 3**C**). The LIHC-specific mRNA nodes were primarily enriched in nuclear receptors meta-pathway, cAMP signaling pathway, metabolism of amino acid and derivatives, biological oxidation, and Rap1 signaling pathways (Fig. 3**D**). The COAD-specific mRNA nodes were primarily enriched in pathways associated with cytokine-cytokine receptor interaction, biological oxidations, vitamin D receptor pathway, metabolism of steroids, and calcium signaling (Fig. 3**E**). Future investigations are warranted to delve deeper into and validate the shared pathways involved in the carcinogenesis of digestive system tumors. Additionally, it is crucial to pay attention to the pathways enriched by specific mRNA nodes. These efforts will enhance our understanding of the molecular mechanisms underlying specific digestive system tumor development.

### 3.4 Construction of shared, semi-shared, and specific lncRNA-miRNA-mRNA networks in digestive system tumors

**Fig. 4:**
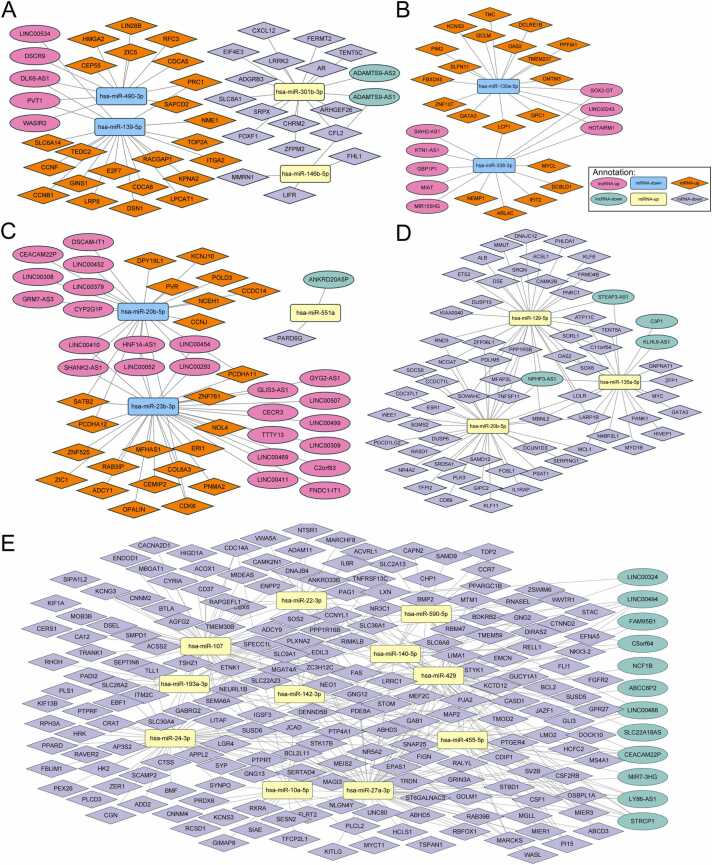
The shared and specific lncRNA-miRNA-mRNA networks in four digestive system tumors. **Panel (A)** presents the shared ceRNA network landscape, while **Panels (B-E)** showcase the specific ceRNA networks related to ESCA, STAD, LIHC, and COAD. (**Annotation**: Pink/green circles indicated up-regulated/down-regulated lncRNAs, yellow/soft blue squares indicated up-regulated/down-regulated miRNAs, orange/light dark blue rhombuses indicated up-regulated/down-regulated mRNAs.).

Using shared differentially expressed RNAs across four cancers, we constructed a shared lncRNA-miRNA-mRNA network. Briefly, we identified 10 lncRNA-miRNA pairs using the miRcode database and 41 miRNA-mRNA pairs using the ENCORI database. Fig. 4**A** illustrates the shared lncRNA-miRNA-mRNA network in digestive system tumors (Table S1). This network includes 5 up-regulated lncRNAs, 2 down-regulated lncRNAs, 2 up-regulated miRNAs, 2 down-regulated miRNAs, 23 up-regulated mRNAs, and 17 down-regulated mRNAs. Additionally, specific lncRNA-miRNA-mRNA networks were constructed for each of the four digestive system tumors using the same analytical approaches (Table S2). Fig. 4**B-E** shows the specific lncRNA-miRNA-mRNA network for each of the four digestive system tumors. The ESCA-specific network comprises 8 lncRNAs, 2 miRNAs, and 20 mRNAs. The STAD-specific network includes 25 lncRNAs, 3 miRNAs, and 24 mRNAs. The LIHC-specific network features 4 lncRNAs, 3 miRNAs, and 62 mRNAs. The COAD-specific network contains 12 lncRNAs, 11 miRNAs, and 196 mRNAs.

**Fig. 5:**
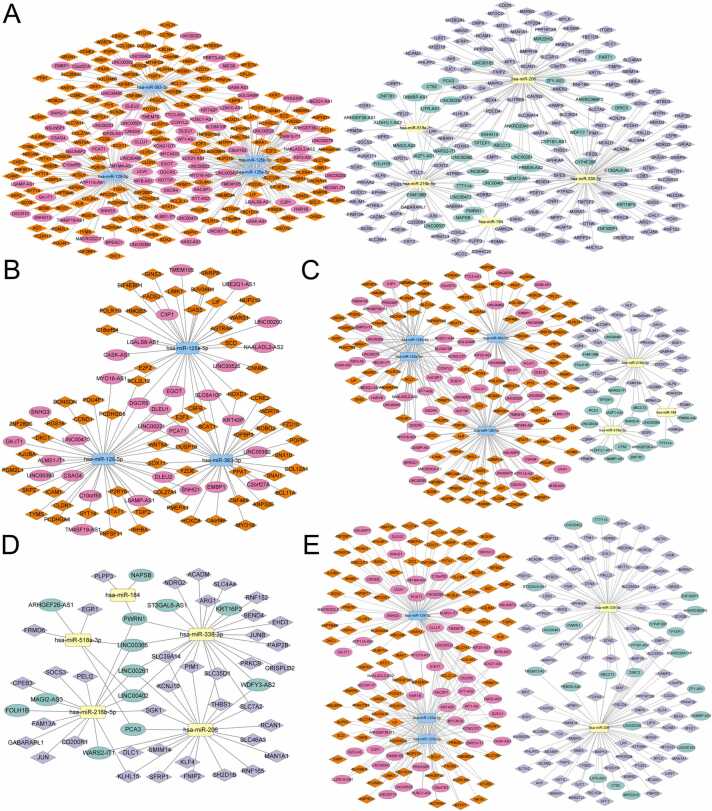
Network illustration of the semi-shared lncRNA-miRNA-mRNA network in four digestive system tumors. Node annotations follow the same format as in Fig. 3. Panel **(A)** showcases the landscape of the ceRNA network, while panels **(B-E)** depict the portions of the network associated with ESCA, STAD, LIHC, and COAD.

A semi-shared ceRNA network was constructed, comprising 117 lncRNAs, 9 miRNAs, and 327 mRNAs, by expanding the analysis of the dysregulated genes network across 2 and 3 digestive tumor types (Fig. 5**A,** Table S3). Fig. 5**B-E** shows the semi-shared lncRNA-miRNA-mRNA network based on the dysregulated RNAs in each digestive system tumor: The ESCA-semi-shared network includes 30 lncRNAs, 3 miRNAs, and 64 mRNAs. The STAD-semi-shared network comprises 74 lncRNAs, 7 miRNAs, and 132 mRNAs. The LIHC-semi-shared network contains 13 lncRNAs, 5 miRNAs, and 39 mRNAs. The COAD-semi-shared network features 71 lncRNAs, 5 miRNAs, and 189 mRNAs. Some genes showed the same dysregulated trend across two or three cancers. For example, RGS16 is upregulated in ESCA and COAD, while FRMD6 is downregulated in STAD and LIHC. Conversely, the lncRNA-miRNA-mRNA network involving the same dysregulated genes can vary between cancers. For example, SGK1 has a “9 lncRNAs/hsa-miR-216b-5p/SGK1” network in STAD, “7 lncRNAs/hsa-miR-216b-5p/SGK1” and “4 lncRNAs/hsa-miR-206/SGK1” in LIHC, “15 lncRNAs/hsa-miR-206/SGK1” network in COAD (Fig. S1, Table S4). Further exploration and investigation of these RNA dysregulation mechanisms are needed.

### 3.5 Screening of prognostic-related RNAs

**Fig. 6:**
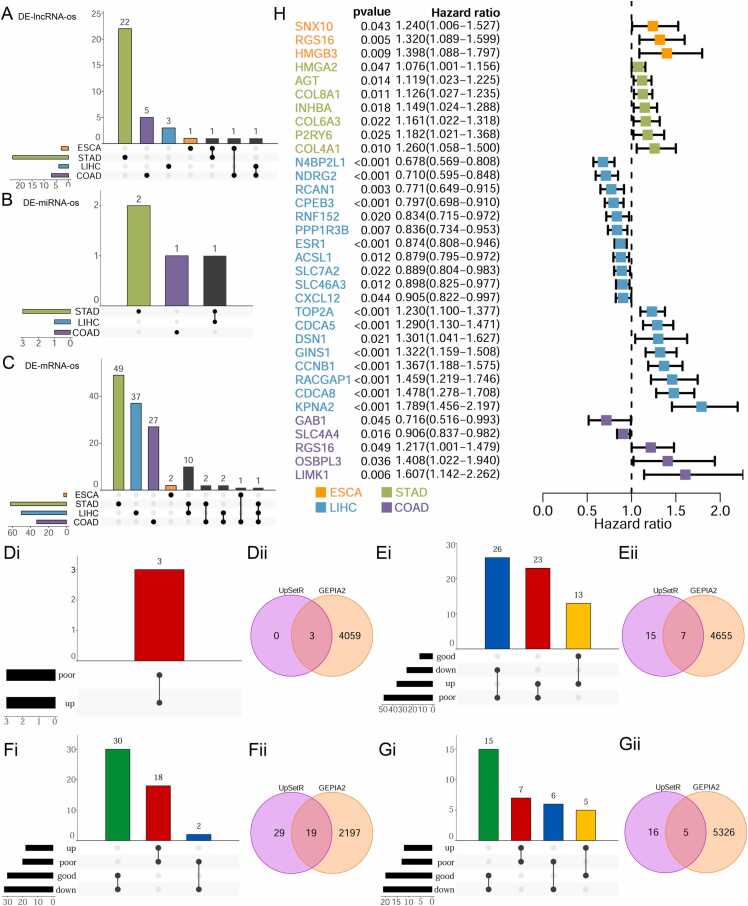
A diagrammatic representation of the prognostic genes identified through univariate Cox regression analysis. Panels **(A-C)** provide a summary of prognostic RNA nodes in the lncRNA-miRNA-mRNA networks. Panels **(Di-Gii)** depict the screening and intersection of key genes for each digestive system tumor, Annotations include indications such as “poor” for poor prognosis, “good” for good prognosis, “up” for up-regulated, and “down” for down-regulated. Panel **(H)** presents a forest plot visualization of key prognostic genes.

The prognostic significance of each RNA node in the shared, specific, and semi-shared lncRNA-miRNA-mRNA networks (Fig. 4, Fig. 5**)** was assessed through univariate Cox regression analysis. This analysis identified 34 lncRNAs, 4 miRNAs, and 131 mRNAs with significant prognostic values (Fig. 6**A-C,** Table S5). In esophageal cancer, 3 lncRNAs and 3 mRNAs showed significant prognostic value. Notably, all these 6 RNAs were derived from nodes within the semi-shared ceRNA network. Furthermore, In stomach adenocarcinoma, 23 lncRNAs, 3 miRNAs, and 62 mRNAs exhibited significant prognostic value. Among these, 21 RNAs originated from nodes within the shared ceRNA network, 10 RNAs were from specific ceRNA network nodes, and 57 RNAs were identified from nodes within the semi-shared ceRNA network. In hepatocellular carcinoma, 4 lncRNAs, 1 miRNA, and 50 mRNAs demonstrated significant prognostic value, including 24 from the shared ceRNA network, 17 from specific ceRNA network nodes, and 14 from the semi-shared ceRNA network. In colon adenocarcinoma, 7 lncRNAs, 1 miRNA, and 33 mRNAs exhibited significant prognostic value, including 5 from the shared ceRNA network, 17 from specific ceRNA network nodes, and 19 from the semi-shared ceRNA network.

**Fig. 7:**
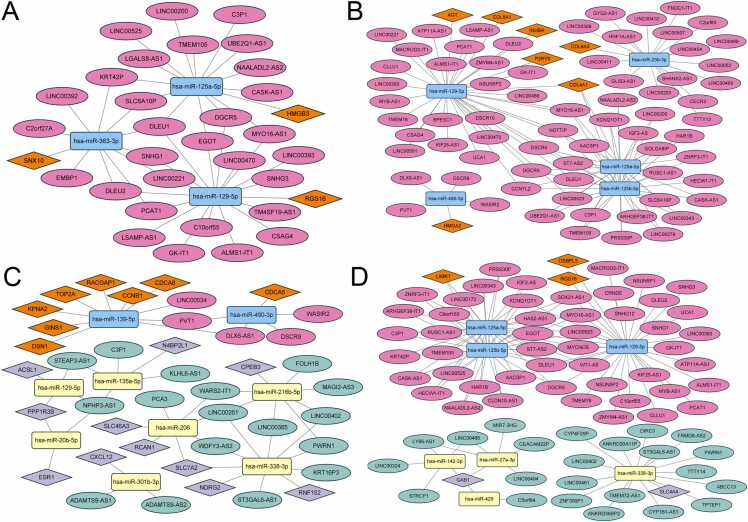
Visualization of sub-ceRNA network. (A-D): ESCA, STAD, LIHC, and COAD-related sub-ceRNA network.

Fig. 6Di, Ei, Fi, and Gi illustrate the differentially expressed RNAs in tumor patients. Briefly, certain upregulated genes (red bars) are associated with poor prognostic outcomes, whereas others are associated with good prognosis (yellow bars); a similar pattern is observed for downregulated genes (green bars vs blue bars). We specifically selected the upregulated genes associated with poor prognosis (red bars) and downregulated genes with good prognosis (green bars). These genes were then re-screened using the GEPIA2 database, followed by an intersection analysis to remove inconsistent RNAs. The remaining genes, identified as key prognostic markers, are visualized in the Venn diagrams (**Figures 6Dii, Eii, Fii, and Gii**). Fig. 6**H** represents the key prognostic genes for each cancer. Additionally, the sub-ceRNA was extracted in the lncRNA-miRNA-mRNA network (Fig. 7). In the sub-ceRNA of ESCA, all three mRNA nodes were from semi-shared network nodes. In the sub-ceRNA of STAD, 1 mRNA node was from shared network nodes, 1 mRNA node was from specific ceRNA network nodes, and 5 mRNA nodes were from semi-shared network nodes. In the sub-ceRNA of LIHC, 9 mRNA nodes were from shared network nodes, 4 mRNA nodes were from specific ceRNA network nodes, and 6 mRNA nodes were all from semi-shared network nodes; In the sub-ceRNA of COAD, 1 mRNA node was from specific ceRNA network nodes and 4 mRNA nodes were from semi-shared network nodes. Displaying the sub-ceRNA networks related to key prognostic mRNAs reveals potential relationships between mRNAs and non-coding RNAs. Furthermore, constructing these sub-ceRNA networks provides a foundation for further research and enables scholars to develop more detailed RNA-dysregulated networks in digestive system tumors.

Since the GEPIA2 database did not include analysis for miRNA, no re-screening for miRNA was conducted. For lncRNA nodes within the ceRNA, priority was given to lncRNAs exhibiting significant differential expression with a consistent trend in both the “edgeR” and GEPIA2 analyses. Key prognostic lncRNAs were identified including up-regulated DLU2 in ESCA, up-regulated DLU2, PVT1, and TPTEP1 in STAD, and down-regulated SNHG14 and C3P1 in LIHC (Fig. S2).

### 3.6 Identification of the putative ceRNA regulatory axes

**Fig. 8:**
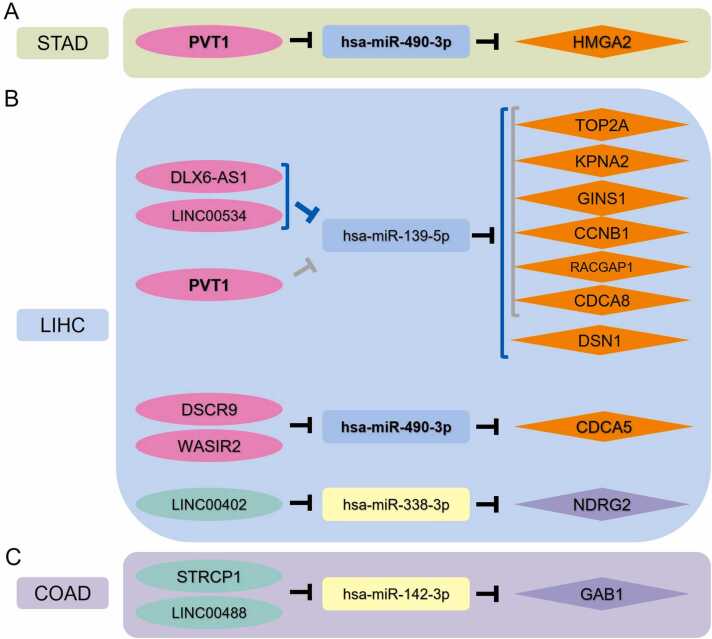
Identification of the putative ceRNA regulatory axes.

Following the principles of the ceRNA hypothesis, correlation analysis was performed on various RNA pairs within the sub-ceRNA network using the ENCORI database. Briefly, in the sub-ceRNA network of each tumor, miRNA-mRNA pairs with significant negative correlation were identified including 1 pair in STAD, 11 pairs in LIHC, and 1 pair in COAD, while no significant pairs were found in ESCA. Due to the absence of significant correlations in ESCA, further analysis and identification of the ESCA-related regulatory axis were not pursued. Furthermore, significant correlations were identified for miRNA-lncRNA pairs:1 pair in STAD, 6 pairs in LIHC, and 2 pairs in COAD (Table S6). Finally, lncRNA-mRNA pairs with significant positive correlation were further screened revealing 1 pair in STAD; 23 pairs in LIHC; and 2 pairs in COAD. Fig. 8 shows the potential ceRNA regulatory axes: 1 regulatory axis involving 3 RNA nodes (1 IncRNA, 1 miRNA, and 1 mRNA) was identified in STAD, 23 regulatory axes involving 18 RNA nodes (6 IncRNAs, 3 miRNAs, and 9 mRNAs) were identified in LIHC and 2 ceRNA regulatory axes involving 4 RNA nodes (2 lncRNAs, 1 miRNA, and 1 mRNA) were identified in COAD (Table 2).

**Table 2:** Results of correlation analysis of the identified ceRNA regulatory axes nodes.

**Types**	**mRNA**	**miRNA**	**lncRNA**	**mRNA-miRNA**		**miRNA-lncRNA**		**mRNA-lncRNA**	
				**Cor**	***P***	**Cor**	***P***	**Cor**	***P***
STAD	HMGA2	miR-490 -3p	PVT1	−0.106	4.19E−02	−0.124	1.69E−02	0.208	5.12E−05
LIHC	TOP2A	miR-139 -5p	DLX6-AS1	−0.472	6.00E−22	−0.183	4.07E−04	0.286	1.86E−08
	DSN1			−0.351	3.63E−12			0.240	2.75E−06
	GINS1			−0.472	5.98E−22			0.322	1.86E−10
	CCNB1			−0.547	2.69E−30			0.306	1.57E−09
	RACGAP1			−0.431	3.84E−18			0.326	1.09E−10
	CDCA8			−0.513	3.18E−26			0.247	1.35E−06
	KPNA2			−0.49	8.91E−24			0.274	7.15E−08
	TOP2A		PVT1	−0.472	6.00E−22	−0.24	2.94E−06	0.133	1.03E−02
	GINS1			−0.472	5.98E−22			0.126	1.48E−02
	CCNB1			−0.547	2.69E−30			0.264	2.12E−07
	RACGAP1			−0.431	3.84E−18			0.190	2.16E−04
	CDCA8			−0.513	3.18E−26			0.172	8.35E−04
	KPNA2			−0.49	8.91E−24			0.287	1.51E−08
	TOP2A		LINC00534	−0.472	6.00E−22	−0.195	1.63E−04	0.224	1.21E−05
	DSN1			−0.351	3.63E−12			0.147	4.45E−03
	GINS1			−0.472	5.98E−22			0.226	1.02E+ 04
	CCNB1			−0.547	2.69E−30			0.239	2.94E−06
	RACGAP1			−0.431	3.84E−18			0.211	3.93E−04
	CDCA8			−0.513	3.18E−26			0.275	6.13E−08
	KPNA2			−0.49	8.91E−24			0.214	3.07E−04
	CDCA5	miR-490 -3p	DSCR9	−0.154	2.94E−3	−0.103	4.82E−2	0.445	1.32E−19
			WASIR2	−0.154	2.94E−3	−0.124	1.67E−2	0.406	2.66E−16
	NDRG2	miR-338 -3p	LINC00402	−0.122	1.85E−2	−0.203	8.09E−5	0.29	1.05E−08
COAD	GAB1	miR-142 -3p	STRCP1	−0.291	3.08E−10	−0.158	7.69E−04	0.225	7.67E−07
			LINC00488	−0.291	3.08E−10	−0.138	3.44E−03	0.225	7.85E−07

## 4 Discussion

Digestive system tumors, including esophageal carcinoma (ESCA), stomach adenocarcinoma (STAD), liver hepatocellular carcinoma (LIHC), and colon adenocarcinoma (COAD), impose a significant health burden globally [19]. Despite significant advances in treatment strategies over the past few years, such as surgery, chemotherapy, targeted therapy, and radiotherapy, patient outcomes remain suboptimal. In this context, personalized and precision medicine offers an evolving paradigm that tailors treatment strategies to the unique molecular and genetic characteristics of each patient, which can significantly enhance therapeutic outcomes.

The ceRNA networks play a key role in carcinogenesis through gene dysregulation. Identifying both shared & specific ceRNA networks across different cancers can enhance our understanding of common etiology. The construction of ceRNA networks in our study presents new potential therapeutic targets by distinguishing specific, shared, and semi-shared ceRNA networks in digestive system tumors. These findings have broad implications for personalized and precision medicine. Understanding common and unique ceRNA networks across different digestive cancers can guide the development of targeted therapies that are customized to the molecular profiles of each cancer. This is crucial as recent research has increasingly demonstrated that ncRNAs, such as miRNAs, lncRNAs, and circRNAs, play critical roles in cancer progression, metastasis, and drug resistance by modulating gene expression and acting as key regulatory elements within ceRNA networks [20], [21]. Identifying common and unique ceRNA networks may lead to the development of targeted therapies and diagnostic tools tailored to the molecular profiles of different cancers [22], [23], [24]. Such advancements are essential for personalized medicine, which aims to improve patient outcomes by customizing treatment strategies based on individual genetic and molecular characteristics.

Our study identified specific, shared, and semi-shared ceRNA networks across digestive system tumors, with substantial implications for diagnostics and therapeutics in these cancers. Recent studies emphasize the evolving role of ncRNA elements in cancer prognosis, treatment, and providing clinical guidelines. For example, Piergentili et al. [25] highlight how ncRNAs can serve as biomarkers for personalized therapeutic strategies, facilitating tailored treatment approaches that enhance patient outcomes. Similarly, Cavaliere et al. [26] explore the implications of ncRNAs in endometrial cancer, reflecting the broader relevance of these findings across different cancers. Furthermore, the legal implications of ambiguous clinical guidelines are addressed by Charles G. Kels and Lori H. Kels [27], emphasizing the need for clear frameworks to support the integration of these innovative approaches into clinical practice. Moreover, as the field of personalized medicine progresses, integrating molecular insights into clinical practice becomes crucial. This development raises important considerations for policy-making and evidence-based guidelines. By incorporating findings related to ncRNAs and their regulatory roles, guidelines can be better informed to address both treatment efficacy and ethical considerations surrounding patient consent and data management.

In this study, data for patients with four types of digestive system tumors (ESCA, STAD, LIHC, and COAD) were obtained from the TCGA database. RNA-pairs relationships data were sourced from miRcode and ENCORI databases. Subsequently, the study focused on analyzing the specific/shared/semi-shared lncRNA-miRNA-mRNA networks in each cancer type individually. Comparing these networks and conducting functional enrichment of DEGs revealed variations in the carcinogenic mechanisms across the digestive tumors. Furthermore, according to ConsensusPathDB results (Fig. 3), ESCA was notably enriched in pathways related to interferon signaling and cytokine signaling disorders. Goedegebuure and Hu et al. demonstrated that future optimization of interferon treatment duration and measurement design of interferon signaling pathways have implications for interferon response to future cancer therapy [28], [29]. Zheng et al. [30] and Bhat et al. [31] demonstrated that inhibiting the expression of specific cytokines such as CXCL6, could be an effective treatment strategy for esophageal cancer. In STAD, pathways like keratinization and the NRF2 pathway were notably enriched. Li et al. [32] demonstrated that targeting keratin-related genes such as KRT23, and transcription factor Nrf2 could serve as potential molecular targets for gastric cancer. In LIHC, the Rap1 signaling pathway and amino acid metabolism were significantly enriched. Mo et al. [33] and Missiaen et al. [34] demonstrated that inhibiting the dysregulated NF-κB/RAP1 signaling pathway or targeting amino acid transporters could offer a viable approach for targeted therapy for HCC in clinical settings. In COAD, enriched pathways included cytokine-cytokine receptor interaction, and vitamin D receptor pathway. Terasaki et al. [35] reported that inhibiting cytokine-cytokine receptor interaction exerts a chemopreventive effect. Additionally, Lu et al. [36] found that activation of the vitamin D receptor pathway can inhibit the epithelial-mesenchymal transition in cancer. From our analysis, cell cycle, DNA Repair, signaling by RHO GTPase, etc., were identified as common potential mechanisms in digestive system tumors, aligning with the previous findings. For example, Matthews et al. [37] and Wang et al. [38] demonstrated the anti-tumor effects of arresting the cell cycle and inhibiting the expression of the RHO GTPase family, such as RHOA.

Univariate Cox regression analysis was used to select ceRNA nodes with prognostic value, and the GEPIA2 database was then used for re-screening, and the respective sub-ceRNA networks were further extracted (Fig. 7). Table 3, Table 4 present the dysregulated expressed key prognostic genes predicted in this study, offering a thorough comparison with previous findings. Specifically, in ESCA, all three predicted prognostic mRNAs (SNX10, RGS16, and HMGB3) have been experimentally demonstrated to be upregulated previously (Tables 3–4). In STAD, among the predicted prognostic biomarkers, five mRNAs (COL4A1, COL6A3, COL8A1, HMGA2, and INHBA) have been revealed to be upregulated in previous experimental studies while one upregulated mRNA (AGT) has been showed in an earlier computational study (Tables 3–4). Additionally, this study discovered P2RY6 as a new prognostic biomarker and indicated that its increased expression is linked to a poor prognosis in STAD. In LIHC, the up-regulation of seven predicted prognostic mRNAs (CDCA5, CDCA8, CCNB1, GINS1, KPNA2, RACGAP1, and TOP2A) and the down-regulation of eight prognostic mRNAs (ACSL1, CPEB3, ESR1, NDRG2, RCAN1, RNF152, SLC46A3, and SLC7A2) have been experimentally reported in earlier studies, while the up-regulation of DSN1 and the down-regulation of CXCL12 have been previously demonstrated in the computational studies (Tables 3–4). Additionally, we predicted two novel prognostic biomarkers, N4BP2L1 and PPP1R3B, and suggested that the abnormally low expressions of these biomarkers were associated with the poor prognosis of LIHC. In COAD, earlier experimental studies have shown the up-regulation of three predicted prognostic mRNAs (RGS16, OSBPL3, and LIMK1) and the down-regulation of one prognostic mRNA (SLC4A4) (Tables 3–4). It is noteworthy that there was controversy regarding the expression trend of GAB1 in COAD. Our analysis showed that GAB1 was down-regulated, consistent with the computational analysis by Liang et al. [39] and Pérez‐Baena et al. [40] However, this contradicts the experimental findings by Bai et al. [41], who reported GAB1 up-regulation. This discrepancy needed to be clarified in future studies. Overall, of the 34 identified key mRNAs, 27 have been validated in earlier experimental studies and 3 in computational studies, three newly identified biomarkers comprising P2RY6 in STAD, N4BP2L1 and PPP1R3B in LIHC need to be further validated through experimental research on their significance and potential as prognostic biomarkers.

**Table 3:** A comparison between the key prognostic mRNAs reported in this study and previous literature.

Cancers	Feature	mRNA	
		Up	Down
ESCA		SNX10, RGS16, HMGB3	\
STAD		COL4A1, COL6A3, COL8A1, HMGA2, INHBA	\
		AGT	\
		P2RY6	\
LIHC		CDCA5, CDCA8, CCNB1, GINS1, KPNA2, RACGAP1, TOP2A	ACSL1, CPEB3, ESR1, NDRG2, RCAN1, RNF152, SLC46A3, SLC7A2
		DSN1	CXCL12
		\	N4BP2L1, PPP1R3B
COAD		LIMK1, OSBPL3, RGS16	SLC4A4
		\	GAB1

Notes

Represent experimentally reported dysregulated mRNAs in corresponding cancers;

Represent computationally reported dysregulated mRNAs in corresponding cancers;

Represent novel dysregulated mRNA predicted in corresponding cancers in this study.

**Table 4:** A summary of the predicted prognostic mRNAs validated in previous literature.

**Cancers**	**mRNA**	**Feature**	**Abstract**
ESCA	HMGB3		Sun et al. [42] demonstrated, through qRT-PCR and western blot analysis, that HMGB3 expression was higher in esophageal cancer tissues. They further found that hypermethylation of the miR-216a led to an increase in its target HMGB3, which subsequently activated the Wnt/β-catenin pathways, thereby promoting esophageal cancer progression.
	RGS16		Zhang et al. [43] demonstrated through qPCR and western blot analysis that RGS16 expression was high in esophageal cancer tissues. They also showed that RGS16 promotes esophageal cell growth and migration by inhibiting the phosphorylation of the Hippo pathway kinase LATS1.
	SNX10		Warnecke-Eberz et al. [44] demonstrated through microarray analysis that the expression of SNX10 was higher in esophageal cancer tissues. They suggest that SNX10 could serve as a potential diagnostic signature for the early detection of tumor cells.
STAD	COL4A1		Liu et al. [45] exhibited that COL4A1 was up-regulated in stomach adenocarcinoma tissues by RT-qPCR.
	COL6A3		Sun et al. [46] demonstrated via qRT-PCR and western blot that COL6A3 was upregulated in gastric cancer tissues. They found that hsa_circ_0006401 sponged miR-3064-5p, alleviating its inhibitory effect on COL6A3, thereby promoting the growth and progression of gastric cancer cells.
	COL8A1		Zhou et al. [47] showed through RT-qPCR that COL8A1 was upregulated in gastric cancer cell lines. Knockdown of COL8A1 inhibited cell proliferation and induced apoptosis in gastric cancer cells.
	HMGA2		Xia et al. [48] revealed through qRT-PCR that HMGA2 was upregulated in gastric cancer tissues. CircFAM73A was found to enhance HMGA2 expression by sponging miR-490-3p, thereby promoting stem cell-like properties and malignancy in gastric cancer cells.
	INHBA		Chen et al. [49] showed that INHBA expression was higher in gastric cancer tissues by immunohistochemistry and RT-qPCR and that the up-regulation of INHBA promoted the progress of GC by activating the TGF-β signaling pathway.
	AGT		Liu et al. [50] showed that AGT was up-regulated in gastric cancer tissues through bioinformatics analysis using three GEO datasets (GSE66229, GSE27342, GSE63089).
LIHC	CDCA5		Wang et al. [51] demonstrated that CDCA5 expression was higher in hepatocellular carcinoma (HCC) tissues, as shown by qPCR and immunohistochemical staining, and that silencing CDCA5 inhibited HCC cell proliferation.
	CDCA8		Cui et al. [52] showed that CDCA8 was up-regulated in HCC, as shown by immunohistochemistry, qRT-PCR, and Western blot, and that the knockdown of CDCA8 inhibited HCC cell proliferation.
	CCNB1		Gu et al. [53] revealed that the up-regulation of CCNB1 could be targeted and inhibited by microRNA-144 and that inhibiting CCNB1 suppressed the invasion, migration, and spread of HCC cells.
	DSN1		Sun et al. [54] showed that the expression of DSN1 was higher in HCC by qPCR.
	GINS1		Li et al. [55] demonstrated that the expression of GINS1 was higher in HCC cell lines by RT-qPCR and that the up-regulation of GINS1 promoted HCC cell growth and sorafenib resistance.
	KPNA2		Guo et al. [56] found that KPNA2 expression was higher in HCC tissues, as predicted by qRT-PCR and confirmed by immunohistochemistry staining, and that the up-regulation of KPNA2 promoted the migration and invasion of HCC cells.
	RACGAP1		Liao et al. [57] exhibited that RACGAP1 expression was higher in HCC tissues, as predicted by immunohistochemical staining and that the high expression of RACGAP1 promoted the proliferation, migration, and invasion capacity of cancer cells.
	TOP2A		Dong et al. [58] demonstrated that TOP2A expression was high in HCC tissues as shown by western blot and RT-qPCR, and that TOP2A promoted epithelial-mesenchymal transition (EMT) in HCC by activating p-ERK1/2/p-SMAD2/Snail pathway.
	ACSL1		Yue et al. [59] demonstrated that ACSL1 expression was lower in HCC tissues by qRT-PCR.
	CPEB3		Hu et al. [60] demonstrated that CPEB3 expression was lower in HCC cell lines as shown by qRT-PCR, and that miR-9-5p/FOXO1/CPEB3 Feed-Forward Loop promoted cell proliferation and tumor progression of HCC.
	ESR1		Hishida et al. [61] demonstrated that ESR1 expression was lower in HCC cell lines as shown by RT-PCR and that this decreased expression was related to the hypermethylation of the promoter.
	NDRG2		Guo et al. [62] showed that NDRG2 expression was lower in HCC tissues, as indicated by immunohistochemistry and Western blot, and that the up-regulation of NDRG2 inhibited the Warburg effect and cell growth of HCC.
	RCAN1		Song et al. [63] demonstrated that RCAN1 expression was lower in HCC tissues as shown by qPCR and that over-expression of CircADAMTS14 inhibited miR-572, thereby increasing RCAN1 expression and inducing apoptosis in HCC cells.
	RNF152		Wan et al. [64] revealed that RNF152 was down-regulated in HCC, as shown by qPCR and western blot, and that RNF152 inhibited HCC progression by deactivating CXCL6 signaling through the ubiquitination and degradation of TSPAN12.
	SLC46A3		Zhao et al. [65] showed that SLC46A3 expression was lower in HCC tissues, as shown by RT-PCR and western blot, and that up-regulation of SLC46A3 inhibited EMT progression and reduced resistance to sorafenib in hepatocellular carcinoma.
	SLC7A2		Xia et al. [66] showed that SLC7A2 expression was lower in HCC tissues, as shown by immunohistochemistry and Western blot, and that down-regulation of SLC7A2 promoted the up-regulation of CXCL1 via the PI3K/Akt/NF-κB pathway, leading to the recruitment of myeloid-derived suppressor cells (MDSCs) and exerting a tumor immunosuppressive effect.
	CXCL12		He et al. [67] demonstrated that CXCL12 expression was lower in HCC through immunohistochemical staining and qRT-PCR.
COAD	LIMK1		Liao et al. [68] demonstrated that LIMK1 expression was higher in colorectal cancer (CRC) tissues as shown by Western blot and immunohistochemistry, and that its over-expression in the cytoplasm and subcellular region of the nucleus-induced EMT and induced the PI3K/Akt signaling pathway, leading to tumor metastasis and poor prognosis.
	OSBPL3		Wang et al. [69] showed that OSBPL3 expression was higher in CRC tissues through qRT-PCR and immunohistochemistry.
	RGS16		Miyoshi et al. [70] demonstrated that expression of RGS16 was higher in CRC tissues by RT-PCR and the high expression of RGS16 might existed as a risk prognostic factor for CRC.
	SLC4A4		Rui et al. [71] showed that SLC4A4 expression was lower in colorectal cancer through qRT-PCR and immunohistochemistry.
	GAB1		Pérez-Baena et al. [40] and Liang et al. [39] demonstrated that GAB1 expression was lower in CRC through computational analysis, consistent with our result. However, Bai et al. [41] revealed that GAB1 expression was higher in CRC tissues, as shown by western blot, and that microRNA-409-3p inhibited GAB1, thereby suppressing cancer invasion and metastasis.

Notes

Represent experimentally reported up/down-regulated mRNA in corresponding tumor types in accordance with our study.

Represent computationally reported up/down regulated mRNA in corresponding tumor types in accordance with our study.

Represent experimentally reported up-regulated mRNA in corresponding tumor types in difference with our study.

Through univariate Cox regression analysis, four prognostic miRNAs associated with the prognosis of digestive tumors were screened out. Table 5 provides a comparison of the aberrant expression levels of the four miRNAs identified in this study with those reported in the existing literature. This study validates previous findings by confirming the down-regulation of hsa-miR-139-5p and hsa-miR-125a-5p in STAD, the down-regulation of hsa-miR-139-5p in LIHC, and the up-regulation of hsa-miR-193a-3p in COAD. Notably, we observed the down-regulation of hsa-miR-125b-5p in gastric cancer, which contrasts with the up-regulation reported in a previous bioinformatics study [74]. It is worth noting that this contradiction might be due to differences in sample sizes. Our results are likely more robust, as our analysis utilized a significantly larger dataset from TCGA-STAD compared to the smaller GEO dataset (GSE93415) used by Zhang et al. [74]. Therefore, further investigation into the expression levels and carcinogenic mechanisms of miR-125b-5p in gastric cancer is necessary.

**Table 5:** A summary of the prognostic miRNAs validated in previous literature.

**Cancers**	**miRNA**	**Feature**	**Abstract**
STAD	miR-125a-5p		Xu et al. [72] demonstrated that hsa-miR-125a-5p was down-regulated in gastric cancer, as shown by qRT-PCR, and that overexpression of miR-125a-5p inhibited the migratory capacity of gastric cancer cells.
	miR-139-5p		Zhang et al. [73] showed that hsa-miR-139-5p was down-regulated in gastric cancer tissues and cell lines, as shown by qRT-PCR, and that hsa-miR-139-5p could target and inhibit SLC39A7, thereby suppressing the proliferation and migration of gastric cancer cells while promoting apoptosis.
	miR-125b-5p		Zhang et al. [74] demonstrated that hsa-miR-125b-5p was up-regulated in gastric cancer, through bioinformatics analysis based on GSE93415.
LIHC	miR-139-5p		Hua et al. [75] revealed that hsa-miR-139-5p was down-regulated in HCC, as shown by qRT-PCR, and that hsa-miR-139-5p inhibited the proliferation and metastasis of HCC cells by targeting ETS1.
COAD	miR-193a-3p		Yong et al. [76] demonstrated that hsa-miR-193a-3p was up-regulated in colorectal cancer, as shown by microarray profiling and RT-PCR.

Notes

Represent experimentally reported up/down-regulated miRNA in corresponding tumor types in accordance with our study.

Represent computationally reported up-regulated miRNA in corresponding tumor types in conflict with our study.

Table 6 illustrates the dysregulated expression of five key prognostic lncRNAs identified in this study and prior cancer experimental studies. Specifically, experimental studies have revealed the up-regulation of DLEU2 in ESCA, up-regulation of both DLEU2 and PVT1, along with down-regulation of TPTEP1 in STAD, and down-regulation of C3P1 in LIHC. A computational study has reported the down-regulation of SNHG14 in STAD [77]. Further investigation and confirmation are needed to understand the abnormal expression level, carcinogenesis mechanism, and RNA-induced network of SNHG14.

**Table 6:** A summary of the prognostic lncRNAs validated in previous literature.

**Cancers**	**lncRNA**	**Feature**	**Abstract**
ESCA	DLEU2		Lu et al. [78] showed through bioinformatic analysis and in vitro experiments that DLEU2 was up-regulated in ESCA, based on data from the GEPIA database. Over-expression of DLEU2 promoted cancer cell proliferation, migration, and invasion while reducing apoptosis.
STAD	DLEU2		Li et al. [79] showed that the expression of DLEU2 was higher in gastric cancer cell lines, as shown by RT-qPCR, and that DLEU2 could down-regulated miR-23b-3p, leading to the higher expression of the target gene NOTCH2. This, in turn, activated the Notch signaling pathway and exacerbated gastric cancer progression.
	PVT1		Han et al. [80] demonstrated that the expression of PVT1 was higher in gastric cancer, as shown by RT-qPCR.
	TPTEP1		Huang et al. [81] demonstrated that expression of TPTEP1 was lower in gastric cancer cell lines, as shown by RT-qPCR, and that TPTEP1 inhibited miR-548d-3p through sponge adsorption, leading to the high expression of the target gene KLF9, thereby inhibiting cancer cell migration and invasion.
	SNHG14		Jin et al. [77] revealed that the expression of SNHG14 was lower in gastric cancer, as shown by computational analysis based on the TCGA database.
LIHC	C3P1		Song et al. [82] demonstrated that C3P1 was down-regulated in HCC, as shown by RT-PCR.

Notes

Represent experimentally reported up/down-regulated lncRNA in corresponding tumor types in accordance with our study.

Represent computationally reported down-regulated lncRNA in corresponding tumor types in accordance with our study.

In this study, we identified key RNA junction pairs through RNA correlation analysis. Despite numerous experimental reports on RNA nodes, the connections between these nodes have not been thoroughly explored. However, some of these connections can be found in the literature. For example, Xia et al. [48] experimentally confirmed the existence of the hsa-miR-490-3p/HMGA component in STAD, which aligns with our RNA correlation analysis findings. Their study further revealed that CircFAM73A upregulated HMGA2 expression by inhibiting miR-490-3p, thereby promoting the stem cell-like properties of GC cells [48]. Zhang et al. [83] and Bin Bao et al. [84] confirmed the existence of hsa-miR-139-5p/TOP2A and hsa-miR-139-5p/CCNB1 in renal clear cell carcinoma and lung adenocarcinoma, respectively, which is consistent with the RNA correlation analysis in LIHC. We aim to provide our results as a resource for researchers examining the particulars of dysregulated RNA networks and potential regulatory pathways in digestive system tumors.

**Fig. 9:**
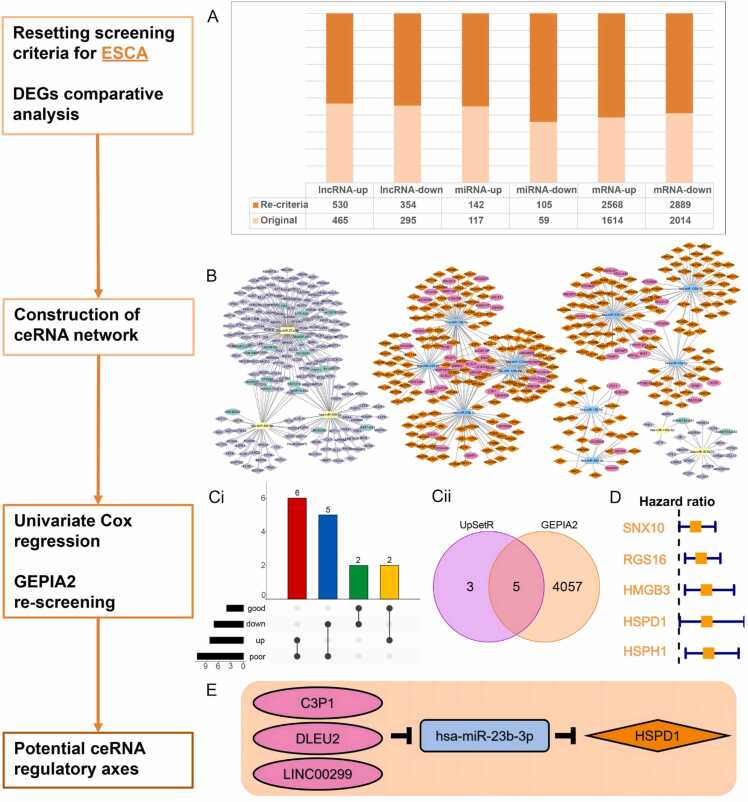
The identification process and results of ceRNA regulatory axes after threshold adjustment for ESCA. (**A**) Statistics of DEGs before and after ESCA adjustment of screening threshold. (**B**) The landscape of the ESCA-related-ceRNA network. (**Ci-Cii**). The screening and intersection of key genes for ESCA. (**D**) A forest plot visualization of ESCA related-key prognostic genes. (**E**) Identification of the potential ceRNA regulatory axes.

The ceRNA comparative analysis of digestive system tumors, especially concerning ESCA, has highlighted several points of interest. However, It is essential to note that limitations in data-comparative analysis may arise due to factors such as the artificial setting of “shared/specific/semi-shared” background conditions and consistent analysis and screening conditions. Consequently, the ceRNA regulatory axis for ESCA remains inconclusive. To address this issue, the screening criteria for ESCA were adjusted to *P*-adj < 0.05 and |log2FC| > 0.585. This adjustment allowed for the inclusion of additional differentially expressed RNAs associated with ESCA. Subsequent intersection analysis of DEGs focused on the dysregulated lncRNA-miRNA-mRNA network identified by ESCA-related DE-RNAs, which included 90 lncRNAs, 15 miRNAs, and 566 mRNAs (Fig. 9B, Fig. S3). Through univariate Cox regression analysis and GEPIA2 differential expression re-screening, SNX10, RGS16, HMGB3, HSPD1, and HSPH1 emerged as the key mRNA nodes associated with ESCA. Additionally, RNA correlation analysis revealed (C3P1, DLEU2, LINC00299)/hsa-miR-23b-3p/HSPD1 as potential ceRNA regulatory axes in ESCA (Fig. 9). Table 7 provides the definitions of RNA nodes that constitute the ESCA regulatory axes before and after threshold adjustment, along with supporting research reports on some RNA pairs. Experimental studies have described the up-regulation of DLEU2 [78], and LINC00299 in ESCA [85]. Furthermore, Jiang et al. [86] performed computational analysis and identified the up-regulation of HSPD1 in ESCA using data from the Human Protein Atlas (HPA) database, aligning with our findings. However, the over-expression of C3P1 in ESCA has not yet been reported. It is noteworthy that the previously reported up-regulation of hsa-miR-23b-3p in esophageal squamous cell carcinoma cell lines was inconsistent with our results, indicating a need for further investigation in future studies [87].

**Table 7:** Changes in ESCA regulatory axes gene definitions before and after threshold adjustment.

**RNA types**	**Partial genes**	**Included Before Threshold Adjustment** **(Yes/No)**	**Included After Threshold Adjustment** **(Yes/No)**	**Threshold adjustment comparison**	
				**|log2FC| >1**	**|log2FC| >0.585**
lncRNA	DLEU2	Yes	Yes	COAD & ESCA & STAD related semi-shared lncRNA	COAD & ESCA & STAD related semi-shared lncRNA
	LINC00299	Yes	Yes	ESCA & STAD & LIHC related semi-shared lncRNA	ESCA & STAD & LIHC related semi-shared lncRNA
	C3P1	Yes	Yes	ESCA & STAD & COAD related semi-shared lncRNA	ESCA & STAD & COAD related semi-shared lncRNA
mRNA	HSPD1	No	Yes	COAD & STAD-related semi-shared mRNA	ESCA & STAD & COAD related semi-shared mRNA
miRNA	miR-23b-3p	No	Yes	STAD-related specific miRNA	ESCA & STAD related semi-shared miRNA

**Note: |log2FC| >1:** Initially used Log2FC threshold which indicates gene expression is at least doubled or halved, and **|log2FC| >0.585:** Log2FC threshold after adjustment which indicates gene expression is increased by at least 1.5 times or decreased by at least 0.67 times

In summary, within the specified background conditions of “shared/specific/semi-shared” based on the same threshold standard in this study, the ceRNA regulatory axis for ESCA has not been identified. It appears that identifying a shared-ceRNA network may be more feasible when comparing 2–3 types of cancer. For the constructing and comparing of ceRNA networks in four digestive system tumors, we aimed to investigate the presence of distinct shared and specific ceRNA networks. By focusing on differentially expressed RNAs, we could identify shared-ceRNA networks across various cancers. When patient clinical information was included for further screening of key prognosis genes, comparisons across different cancers were more likely to reveal common RNA nodes rather than a complete network (axes). The identification of sub-ceRNA networks appeared to be more inclined toward a single cancer type. At the outset, it is important to acknowledge potential limitations, such as variations in cancer types and sample sizes, which could pose challenges in constructing shared networks. Despite these challenges, our study reveals the existence of a semi-shared network when comparing RNA networks across different cancers, with relatively fewer instances of shared and specific networks. This research provides valuable insights for future cross-cancer analyses of RNA networks in digestive system tumors. To enhance precision in identifying shared and specific ceRNA-related mechanisms across digestive system tumors, future investigations may benefit from larger sample sizes and more detailed analyses spanning various cancer types.

## 5 Conclusions

This novel approach constructed and compared shared and specific lncRNA-miRNA-mRNA networks across four digestive system tumors, revealing potential shared and distinct disease biology mediated by RNA. The functional enrichment analysis of these shared-specific genes provides valuable insights into pathways associated with digestive system tumors, enhancing our understanding of shared and specific carcinogenesis. Additionally, by constructing and comparing competing endogenous RNA (ceRNA) networks across different cancers, the study identifies new potential cancer prognostic markers. N4BP2L1 and PPP1R3B in LIHC, P2RY6 in STAD, and C3P1 in ESCA are identified for the first time, emerging as potential biomarkers. Overall, this innovative study serves as a valuable foundation for future cross-cancer analyses, particularly in the context of digestive system tumor-related RNA networks. These findings contribute to advancing our comprehension of the molecular mechanisms underlying these cancers and offer potential avenues for precision medicine.

## Funding

This research was funded by the Key Discipline Construction Project of Guangdong Medical University (4SG23004G) and the National Undergraduate Training Program for Innovation and Entrepreneurship (202210571009).

## CRediT authorship contribution statement

**Zunnan Huang:** Writing – review & editing, Supervision, Resources, Project administration, Methodology, Funding acquisition, Conceptualization. **Siying Lin:** Writing – original draft, Validation, Investigation, Formal analysis. **Yiming Shao:** Writing – review & editing, Supervision. **Aamir Fahira:** Writing – review & editing, Writing – original draft, Investigation, Formal analysis, Conceptualization. **Yulai Tang:** Writing – review & editing, Writing – original draft, Visualization, Validation, Software, Methodology, Investigation, Formal analysis, Data curation, Conceptualization.

## Declaration of Competing Interest

The authors declare no conflicts of interest.
