# Supplementary material for: Shared and specific competing endogenous RNAs network mining in four digestive system tumors
Source: Comput Struct Biotechnol J. 2024 Nov 5;23:4271–87. doi: 10.1016/j.csbj.2024.11.005 (PMC11635987; doi:10.1016/j.csbj.2024.11.005)
Supplement: Supplementary file 1 — Supplementary material [file mmc1.docx]

**Shared and specific competing endogenous RNAs network mining in four digestive system tumors**

Yulai Tang^#^, Aamir Fahira^#^, Siying Lin, Yiming Shao, Zunnan Huang^*^

#These authors contributed equally to this work.

*corresponding author

**Supplementary materials:**

**Table S1**. Shared ceRNA network nodes.

| node1 | node2 | line |
| --- | --- | --- |
| DSCR9 | hsa-miR-490-3p | lncRNA-up_miRNA-down |
| WASIR2 | hsa-miR-490-3p | lncRNA-up_miRNA-down |
| DLX6-AS1 | hsa-miR-490-3p | lncRNA-up_miRNA-down |
| PVT1 | hsa-miR-490-3p | lncRNA-up_miRNA-down |
| DLX6-AS1 | hsa-miR-139-5p | lncRNA-up_miRNA-down |
| PVT1 | hsa-miR-139-5p | lncRNA-up_miRNA-down |
| LINC00534 | hsa-miR-139-5p | lncRNA-up_miRNA-down |
| hsa-miR-490-3p | CEP55 | miRNA-down_mRNA-up |
| hsa-miR-490-3p | CDCA5 | miRNA-down_mRNA-up |
| hsa-miR-490-3p | HMGA2 | miRNA-down_mRNA-up |
| hsa-miR-490-3p | ZIC5 | miRNA-down_mRNA-up |
| hsa-miR-490-3p | RFC3 | miRNA-down_mRNA-up |
| hsa-miR-490-3p | PRC1 | miRNA-down_mRNA-up |
| hsa-miR-490-3p | LIN28B | miRNA-down_mRNA-up |
| hsa-miR-490-3p | SAPCD2 | miRNA-down_mRNA-up |
| hsa-miR-139-5p | LRP8 | miRNA-down_mRNA-up |
| hsa-miR-139-5p | CDCA8 | miRNA-down_mRNA-up |
| hsa-miR-139-5p | RACGAP1 | miRNA-down_mRNA-up |
| hsa-miR-139-5p | E2F7 | miRNA-down_mRNA-up |
| hsa-miR-139-5p | PRC1 | miRNA-down_mRNA-up |
| hsa-miR-139-5p | CCNF | miRNA-down_mRNA-up |
| hsa-miR-139-5p | TEDC2 | miRNA-down_mRNA-up |
| hsa-miR-139-5p | TOP2A | miRNA-down_mRNA-up |
| hsa-miR-139-5p | NME1 | miRNA-down_mRNA-up |
| hsa-miR-139-5p | KPNA2 | miRNA-down_mRNA-up |
| hsa-miR-139-5p | DSN1 | miRNA-down_mRNA-up |
| hsa-miR-139-5p | GINS1 | miRNA-down_mRNA-up |
| hsa-miR-139-5p | LPCAT1 | miRNA-down_mRNA-up |
| hsa-miR-139-5p | ITGA2 | miRNA-down_mRNA-up |
| hsa-miR-139-5p | CCNB1 | miRNA-down_mRNA-up |
| hsa-miR-139-5p | SLC6A14 | miRNA-down_mRNA-up |
| ADAMTS9-AS1 | hsa-miR-301b-3p | lncRNA-down_miRNA-up |
| ADAMTS9-AS2 | hsa-miR-301b-3p | lncRNA-down_miRNA-up |
| ADAMTS9-AS1 | hsa-miR-146b-5p | lncRNA-down_miRNA-up |
| hsa-miR-146b-5p | MMRN1 | miRNA-up_mRNA-down |
| hsa-miR-146b-5p | LIFR | miRNA-up_mRNA-down |
| hsa-miR-146b-5p | FHL1 | miRNA-up_mRNA-down |
| hsa-miR-301b-3p | TENT5C | miRNA-up_mRNA-down |
| hsa-miR-301b-3p | CXCL12 | miRNA-up_mRNA-down |
| hsa-miR-301b-3p | LRRK2 | miRNA-up_mRNA-down |
| hsa-miR-301b-3p | CFL2 | miRNA-up_mRNA-down |
| hsa-miR-301b-3p | FERMT2 | miRNA-up_mRNA-down |
| hsa-miR-301b-3p | FOXF1 | miRNA-up_mRNA-down |
| hsa-miR-301b-3p | SLC8A1 | miRNA-up_mRNA-down |
| hsa-miR-301b-3p | EIF4E3 | miRNA-up_mRNA-down |
| hsa-miR-301b-3p | ARHGEF26 | miRNA-up_mRNA-down |
| hsa-miR-301b-3p | ADGRB3 | miRNA-up_mRNA-down |
| hsa-miR-301b-3p | CHRM2 | miRNA-up_mRNA-down |
| hsa-miR-301b-3p | ZFPM2 | miRNA-up_mRNA-down |
| hsa-miR-301b-3p | SRPX | miRNA-up_mRNA-down |
| hsa-miR-301b-3p | AR | miRNA-up_mRNA-down |

**Table S2.** Specific ceRNA network nodes.

| specific network node types | node1 | node2 | line |
| --- | --- | --- | --- |
| ESCA | KTN1-AS1 | hsa-miR-338-3p | lncRNA up_miRNA-down |
| ESCA | LINC00243 | hsa-miR-338-3p | lncRNA up_miRNA-down |
| ESCA | GBP1P1 | hsa-miR-338-3p | lncRNA up_miRNA-down |
| ESCA | MIAT | hsa-miR-338-3p | lncRNA up_miRNA-down |
| ESCA | HOTAIRM1 | hsa-miR-338-3p | lncRNA up_miRNA-down |
| ESCA | MIR155HG | hsa-miR-338-3p | lncRNA up_miRNA-down |
| ESCA | SOX2-OT | hsa-miR-338-3p | lncRNA up_miRNA-down |
| ESCA | SIAH2-AS1 | hsa-miR-338-3p | lncRNA up_miRNA-down |
| ESCA | LINC00243 | hsa-miR-135a-5p | lncRNA up_miRNA-down |
| ESCA | HOTAIRM1 | hsa-miR-135a-5p | lncRNA up_miRNA-down |
| ESCA | SOX2-OT | hsa-miR-135a-5p | lncRNA up_miRNA-down |
| ESCA | hsa-miR-338-3p | MYCL | miRNA-down_mRNA-up |
| ESCA | hsa-miR-338-3p | IFIT2 | miRNA-down_mRNA-up |
| ESCA | hsa-miR-338-3p | NEMP1 | miRNA-down_mRNA-up |
| ESCA | hsa-miR-338-3p | LCP1 | miRNA-down_mRNA-up |
| ESCA | hsa-miR-338-3p | ARL4C | miRNA-down_mRNA-up |
| ESCA | hsa-miR-338-3p | DCBLD1 | miRNA-down_mRNA-up |
| ESCA | hsa-miR-135a-5p | GCLM | miRNA-down_mRNA-up |
| ESCA | hsa-miR-135a-5p | DCLRE1B | miRNA-down_mRNA-up |
| ESCA | hsa-miR-135a-5p | GATA3 | miRNA-down_mRNA-up |
| ESCA | hsa-miR-135a-5p | PPFIA1 | miRNA-down_mRNA-up |
| ESCA | hsa-miR-135a-5p | OAS2 | miRNA-down_mRNA-up |
| ESCA | hsa-miR-135a-5p | LCP1 | miRNA-down_mRNA-up |
| ESCA | hsa-miR-135a-5p | CMTM3 | miRNA-down_mRNA-up |
| ESCA | hsa-miR-135a-5p | SLFN11 | miRNA-down_mRNA-up |
| ESCA | hsa-miR-135a-5p | TMEM237 | miRNA-down_mRNA-up |
| ESCA | hsa-miR-135a-5p | KCNS3 | miRNA-down_mRNA-up |
| ESCA | hsa-miR-135a-5p | GPC1 | miRNA-down_mRNA-up |
| ESCA | hsa-miR-135a-5p | FBXO45 | miRNA-down_mRNA-up |
| ESCA | hsa-miR-135a-5p | ZNF107 | miRNA-down_mRNA-up |
| ESCA | hsa-miR-135a-5p | TNC | miRNA-down_mRNA-up |
| ESCA | hsa-miR-135a-5p | PIM2 | miRNA-down_mRNA-up |
| STAD | C2orf83 | hsa-miR-23b-3p | lncRNA up_miRNA-down |
| STAD | LINC00469 | hsa-miR-23b-3p | lncRNA up_miRNA-down |
| STAD | TTTY13 | hsa-miR-23b-3p | lncRNA up_miRNA-down |
| STAD | SHANK2-AS1 | hsa-miR-23b-3p | lncRNA up_miRNA-down |
| STAD | LINC00454 | hsa-miR-23b-3p | lncRNA up_miRNA-down |
| STAD | LINC00411 | hsa-miR-23b-3p | lncRNA up_miRNA-down |
| STAD | LINC00309 | hsa-miR-23b-3p | lncRNA up_miRNA-down |
| STAD | LINC00410 | hsa-miR-23b-3p | lncRNA up_miRNA-down |
| STAD | FNDC1-IT1 | hsa-miR-23b-3p | lncRNA up_miRNA-down |
| STAD | GYG2-AS1 | hsa-miR-23b-3p | lncRNA up_miRNA-down |
| STAD | GLIS3-AS1 | hsa-miR-23b-3p | lncRNA up_miRNA-down |
| STAD | HNF1A-AS1 | hsa-miR-23b-3p | lncRNA up_miRNA-down |
| STAD | CECR3 | hsa-miR-23b-3p | lncRNA up_miRNA-down |
| STAD | LINC00499 | hsa-miR-23b-3p | lncRNA up_miRNA-down |
| STAD | LINC00293 | hsa-miR-23b-3p | lncRNA up_miRNA-down |
| STAD | LINC00507 | hsa-miR-23b-3p | lncRNA up_miRNA-down |
| STAD | LINC00052 | hsa-miR-23b-3p | lncRNA up_miRNA-down |
| STAD | CYP2G1P | hsa-miR-20b-5p | lncRNA up_miRNA-down |
| STAD | LINC00308 | hsa-miR-20b-5p | lncRNA up_miRNA-down |
| STAD | GRM7-AS3 | hsa-miR-20b-5p | lncRNA up_miRNA-down |
| STAD | SHANK2-AS1 | hsa-miR-20b-5p | lncRNA up_miRNA-down |
| STAD | LINC00454 | hsa-miR-20b-5p | lncRNA up_miRNA-down |
| STAD | LINC00452 | hsa-miR-20b-5p | lncRNA up_miRNA-down |
| STAD | LINC00379 | hsa-miR-20b-5p | lncRNA up_miRNA-down |
| STAD | CEACAM22P | hsa-miR-20b-5p | lncRNA up_miRNA-down |
| STAD | LINC00410 | hsa-miR-20b-5p | lncRNA up_miRNA-down |
| STAD | DSCAM-IT1 | hsa-miR-20b-5p | lncRNA up_miRNA-down |
| STAD | HNF1A-AS1 | hsa-miR-20b-5p | lncRNA up_miRNA-down |
| STAD | LINC00293 | hsa-miR-20b-5p | lncRNA up_miRNA-down |
| STAD | LINC00052 | hsa-miR-20b-5p | lncRNA up_miRNA-down |
| STAD | ANKRD20A5P | hsa-miR-551a | lncRNA-down_miRNA-up |
| STAD | hsa-miR-23b-3p | OPALIN | miRNA-down_mRNA-up |
| STAD | hsa-miR-23b-3p | RAB3IP | miRNA-down_mRNA-up |
| STAD | hsa-miR-23b-3p | NOL4 | miRNA-down_mRNA-up |
| STAD | hsa-miR-23b-3p | ZNF525 | miRNA-down_mRNA-up |
| STAD | hsa-miR-23b-3p | ZNF761 | miRNA-down_mRNA-up |
| STAD | hsa-miR-23b-3p | SATB2 | miRNA-down_mRNA-up |
| STAD | hsa-miR-23b-3p | COL6A3 | miRNA-down_mRNA-up |
| STAD | hsa-miR-23b-3p | ZIC1 | miRNA-down_mRNA-up |
| STAD | hsa-miR-23b-3p | PCDHA11 | miRNA-down_mRNA-up |
| STAD | hsa-miR-23b-3p | PCDHA12 | miRNA-down_mRNA-up |
| STAD | hsa-miR-23b-3p | CDK6 | miRNA-down_mRNA-up |
| STAD | hsa-miR-23b-3p | ADCY1 | miRNA-down_mRNA-up |
| STAD | hsa-miR-23b-3p | MFHAS1 | miRNA-down_mRNA-up |
| STAD | hsa-miR-23b-3p | PNMA2 | miRNA-down_mRNA-up |
| STAD | hsa-miR-23b-3p | ERI1 | miRNA-down_mRNA-up |
| STAD | hsa-miR-23b-3p | CEMIP2 | miRNA-down_mRNA-up |
| STAD | hsa-miR-20b-5p | KCNJ10 | miRNA-down_mRNA-up |
| STAD | hsa-miR-20b-5p | CCNJ | miRNA-down_mRNA-up |
| STAD | hsa-miR-20b-5p | POLD3 | miRNA-down_mRNA-up |
| STAD | hsa-miR-20b-5p | PVR | miRNA-down_mRNA-up |
| STAD | hsa-miR-20b-5p | SATB2 | miRNA-down_mRNA-up |
| STAD | hsa-miR-20b-5p | CCDC14 | miRNA-down_mRNA-up |
| STAD | hsa-miR-20b-5p | NCEH1 | miRNA-down_mRNA-up |
| STAD | hsa-miR-20b-5p | PCDHA11 | miRNA-down_mRNA-up |
| STAD | hsa-miR-20b-5p | PCDHA12 | miRNA-down_mRNA-up |
| STAD | hsa-miR-20b-5p | DPY19L1 | miRNA-down_mRNA-up |
| STAD | hsa-miR-20b-5p | CDK6 | miRNA-down_mRNA-up |
| STAD | hsa-miR-551a | PARD6G | miRNA-up_mRNA-down |
| LIHC | STEAP3-AS1 | hsa-miR-129-5p | lncRNA-down_miRNA-up |
| LIHC | NPHP3-AS1 | hsa-miR-129-5p | lncRNA-down_miRNA-up |
| LIHC | C3P1 | hsa-miR-135a-5p | lncRNA-down_miRNA-up |
| LIHC | STEAP3-AS1 | hsa-miR-135a-5p | lncRNA-down_miRNA-up |
| LIHC | KLHL6-AS1 | hsa-miR-135a-5p | lncRNA-down_miRNA-up |
| LIHC | NPHP3-AS1 | hsa-miR-135a-5p | lncRNA-down_miRNA-up |
| LIHC | NPHP3-AS1 | hsa-miR-20b-5p | lncRNA-down_miRNA-up |
| LIHC | hsa-miR-135a-5p | MCL1 | miRNA-up_mRNA-down |
| LIHC | hsa-miR-135a-5p | PANK1 | miRNA-up_mRNA-down |
| LIHC | hsa-miR-135a-5p | GATA3 | miRNA-up_mRNA-down |
| LIHC | hsa-miR-135a-5p | SORL1 | miRNA-up_mRNA-down |
| LIHC | hsa-miR-135a-5p | OAS2 | miRNA-up_mRNA-down |
| LIHC | hsa-miR-135a-5p | N4BP2L1 | miRNA-up_mRNA-down |
| LIHC | hsa-miR-135a-5p | MBNL2 | miRNA-up_mRNA-down |
| LIHC | hsa-miR-135a-5p | GNPNAT1 | miRNA-up_mRNA-down |
| LIHC | hsa-miR-135a-5p | ZFP1 | miRNA-up_mRNA-down |
| LIHC | hsa-miR-135a-5p | LDLR | miRNA-up_mRNA-down |
| LIHC | hsa-miR-135a-5p | MYO1B | miRNA-up_mRNA-down |
| LIHC | hsa-miR-135a-5p | LARP1B | miRNA-up_mRNA-down |
| LIHC | hsa-miR-135a-5p | TENT5A | miRNA-up_mRNA-down |
| LIHC | hsa-miR-135a-5p | HIVEP1 | miRNA-up_mRNA-down |
| LIHC | hsa-miR-135a-5p | MYC | miRNA-up_mRNA-down |
| LIHC | hsa-miR-135a-5p | ATP11C | miRNA-up_mRNA-down |
| LIHC | hsa-miR-129-5p | KIAA0040 | miRNA-up_mRNA-down |
| LIHC | hsa-miR-129-5p | DUSP10 | miRNA-up_mRNA-down |
| LIHC | hsa-miR-129-5p | KLF6 | miRNA-up_mRNA-down |
| LIHC | hsa-miR-129-5p | DNAJC12 | miRNA-up_mRNA-down |
| LIHC | hsa-miR-129-5p | SRGN | miRNA-up_mRNA-down |
| LIHC | hsa-miR-129-5p | SOX6 | miRNA-up_mRNA-down |
| LIHC | hsa-miR-129-5p | C11orf54 | miRNA-up_mRNA-down |
| LIHC | hsa-miR-129-5p | SORL1 | miRNA-up_mRNA-down |
| LIHC | hsa-miR-129-5p | PHLDA1 | miRNA-up_mRNA-down |
| LIHC | hsa-miR-129-5p | OAS2 | miRNA-up_mRNA-down |
| LIHC | hsa-miR-129-5p | TNFSF11 | miRNA-up_mRNA-down |
| LIHC | hsa-miR-129-5p | MBNL2 | miRNA-up_mRNA-down |
| LIHC | hsa-miR-129-5p | ZFP36L1 | miRNA-up_mRNA-down |
| LIHC | hsa-miR-129-5p | LDLR | miRNA-up_mRNA-down |
| LIHC | hsa-miR-129-5p | RND3 | miRNA-up_mRNA-down |
| LIHC | hsa-miR-129-5p | SOWAHC | miRNA-up_mRNA-down |
| LIHC | hsa-miR-129-5p | ETS2 | miRNA-up_mRNA-down |
| LIHC | hsa-miR-129-5p | FRMD4B | miRNA-up_mRNA-down |
| LIHC | hsa-miR-129-5p | MFAP3L | miRNA-up_mRNA-down |
| LIHC | hsa-miR-129-5p | ACSL1 | miRNA-up_mRNA-down |
| LIHC | hsa-miR-129-5p | ALB | miRNA-up_mRNA-down |
| LIHC | hsa-miR-129-5p | PDLIM5 | miRNA-up_mRNA-down |
| LIHC | hsa-miR-129-5p | LARP1B | miRNA-up_mRNA-down |
| LIHC | hsa-miR-129-5p | MMUT | miRNA-up_mRNA-down |
| LIHC | hsa-miR-129-5p | TENT5A | miRNA-up_mRNA-down |
| LIHC | hsa-miR-129-5p | PNRC1 | miRNA-up_mRNA-down |
| LIHC | hsa-miR-129-5p | DSE | miRNA-up_mRNA-down |
| LIHC | hsa-miR-129-5p | NCOA7 | miRNA-up_mRNA-down |
| LIHC | hsa-miR-129-5p | CAMK2B | miRNA-up_mRNA-down |
| LIHC | hsa-miR-129-5p | PPP1R3B | miRNA-up_mRNA-down |
| LIHC | hsa-miR-129-5p | ATP11C | miRNA-up_mRNA-down |
| LIHC | hsa-miR-20b-5p | MCL1 | miRNA-up_mRNA-down |
| LIHC | hsa-miR-20b-5p | PLK3 | miRNA-up_mRNA-down |
| LIHC | hsa-miR-20b-5p | GIPC2 | miRNA-up_mRNA-down |
| LIHC | hsa-miR-20b-5p | FOSL1 | miRNA-up_mRNA-down |
| LIHC | hsa-miR-20b-5p | WEE1 | miRNA-up_mRNA-down |
| LIHC | hsa-miR-20b-5p | SERPING1 | miRNA-up_mRNA-down |
| LIHC | hsa-miR-20b-5p | CD69 | miRNA-up_mRNA-down |
| LIHC | hsa-miR-20b-5p | DUSP6 | miRNA-up_mRNA-down |
| LIHC | hsa-miR-20b-5p | TNFSF11 | miRNA-up_mRNA-down |
| LIHC | hsa-miR-20b-5p | MBNL2 | miRNA-up_mRNA-down |
| LIHC | hsa-miR-20b-5p | ZFP36L1 | miRNA-up_mRNA-down |
| LIHC | hsa-miR-20b-5p | DCUN1D3 | miRNA-up_mRNA-down |
| LIHC | hsa-miR-20b-5p | RASD1 | miRNA-up_mRNA-down |
| LIHC | hsa-miR-20b-5p | SOCS6 | miRNA-up_mRNA-down |
| LIHC | hsa-miR-20b-5p | LDLR | miRNA-up_mRNA-down |
| LIHC | hsa-miR-20b-5p | RND3 | miRNA-up_mRNA-down |
| LIHC | hsa-miR-20b-5p | NR4A2 | miRNA-up_mRNA-down |
| LIHC | hsa-miR-20b-5p | KLF11 | miRNA-up_mRNA-down |
| LIHC | hsa-miR-20b-5p | SOWAHC | miRNA-up_mRNA-down |
| LIHC | hsa-miR-20b-5p | IL1RAP | miRNA-up_mRNA-down |
| LIHC | hsa-miR-20b-5p | MFAP3L | miRNA-up_mRNA-down |
| LIHC | hsa-miR-20b-5p | PDLIM5 | miRNA-up_mRNA-down |
| LIHC | hsa-miR-20b-5p | SGMS2 | miRNA-up_mRNA-down |
| LIHC | hsa-miR-20b-5p | LARP1B | miRNA-up_mRNA-down |
| LIHC | hsa-miR-20b-5p | SRD5A1 | miRNA-up_mRNA-down |
| LIHC | hsa-miR-20b-5p | NCOA7 | miRNA-up_mRNA-down |
| LIHC | hsa-miR-20b-5p | ESR1 | miRNA-up_mRNA-down |
| LIHC | hsa-miR-20b-5p | TFPI2 | miRNA-up_mRNA-down |
| LIHC | hsa-miR-20b-5p | CCDC71L | miRNA-up_mRNA-down |
| LIHC | hsa-miR-20b-5p | PPP1R3B | miRNA-up_mRNA-down |
| LIHC | hsa-miR-20b-5p | SAMD12 | miRNA-up_mRNA-down |
| LIHC | hsa-miR-20b-5p | CDC37L1 | miRNA-up_mRNA-down |
| LIHC | hsa-miR-20b-5p | PDCD1LG2 | miRNA-up_mRNA-down |
| LIHC | hsa-miR-20b-5p | PSAT1 | miRNA-up_mRNA-down |
| COAD | STRCP1 | hsa-miR-140-5p | lncRNA-down_miRNA-up |
| COAD | C5orf64 | hsa-miR-140-5p | lncRNA-down_miRNA-up |
| COAD | CEACAM22P | hsa-miR-140-5p | lncRNA-down_miRNA-up |
| COAD | STRCP1 | hsa-miR-142-3p | lncRNA-down_miRNA-up |
| COAD | LINC00324 | hsa-miR-142-3p | lncRNA-down_miRNA-up |
| COAD | LINC00488 | hsa-miR-142-3p | lncRNA-down_miRNA-up |
| COAD | LY86-AS1 | hsa-miR-142-3p | lncRNA-down_miRNA-up |
| COAD | STRCP1 | hsa-miR-193a-3p | lncRNA-down_miRNA-up |
| COAD | CEACAM22P | hsa-miR-193a-3p | lncRNA-down_miRNA-up |
| COAD | LINC00494 | hsa-miR-193a-3p | lncRNA-down_miRNA-up |
| COAD | STRCP1 | hsa-miR-22-3p | lncRNA-down_miRNA-up |
| COAD | LINC00324 | hsa-miR-22-3p | lncRNA-down_miRNA-up |
| COAD | SLC22A18AS | hsa-miR-22-3p | lncRNA-down_miRNA-up |
| COAD | STRCP1 | hsa-miR-24-3p | lncRNA-down_miRNA-up |
| COAD | NCF1B | hsa-miR-24-3p | lncRNA-down_miRNA-up |
| COAD | LINC00488 | hsa-miR-24-3p | lncRNA-down_miRNA-up |
| COAD | LY86-AS1 | hsa-miR-24-3p | lncRNA-down_miRNA-up |
| COAD | FAM95B1 | hsa-miR-24-3p | lncRNA-down_miRNA-up |
| COAD | CEACAM22P | hsa-miR-24-3p | lncRNA-down_miRNA-up |
| COAD | LINC00494 | hsa-miR-24-3p | lncRNA-down_miRNA-up |
| COAD | SLC22A18AS | hsa-miR-24-3p | lncRNA-down_miRNA-up |
| COAD | ABCC6P2 | hsa-miR-24-3p | lncRNA-down_miRNA-up |
| COAD | STRCP1 | hsa-miR-107 | lncRNA-down_miRNA-up |
| COAD | LINC00488 | hsa-miR-107 | lncRNA-down_miRNA-up |
| COAD | LY86-AS1 | hsa-miR-107 | lncRNA-down_miRNA-up |
| COAD | LINC00494 | hsa-miR-107 | lncRNA-down_miRNA-up |
| COAD | MIR7-3HG | hsa-miR-27a-3p | lncRNA-down_miRNA-up |
| COAD | LINC00488 | hsa-miR-27a-3p | lncRNA-down_miRNA-up |
| COAD | LY86-AS1 | hsa-miR-27a-3p | lncRNA-down_miRNA-up |
| COAD | CEACAM22P | hsa-miR-27a-3p | lncRNA-down_miRNA-up |
| COAD | LINC00494 | hsa-miR-27a-3p | lncRNA-down_miRNA-up |
| COAD | MIR7-3HG | hsa-miR-455-5p | lncRNA-down_miRNA-up |
| COAD | LINC00488 | hsa-miR-455-5p | lncRNA-down_miRNA-up |
| COAD | CEACAM22P | hsa-miR-455-5p | lncRNA-down_miRNA-up |
| COAD | C5orf64 | hsa-miR-429 | lncRNA-down_miRNA-up |
| COAD | C5orf64 | hsa-miR-590-5p | lncRNA-down_miRNA-up |
| COAD | LINC00488 | hsa-miR-590-5p | lncRNA-down_miRNA-up |
| COAD | LY86-AS1 | hsa-miR-10a-5p | lncRNA-down_miRNA-up |
| COAD | FAM95B1 | hsa-miR-10a-5p | lncRNA-down_miRNA-up |
| COAD | LINC00494 | hsa-miR-10a-5p | lncRNA-down_miRNA-up |
| COAD | hsa-miR-140-5p | TMEM59 | miRNA-up_mRNA-down |
| COAD | hsa-miR-140-5p | GNG12 | miRNA-up_mRNA-down |
| COAD | hsa-miR-140-5p | IGSF3 | miRNA-up_mRNA-down |
| COAD | hsa-miR-140-5p | DNAJB4 | miRNA-up_mRNA-down |
| COAD | hsa-miR-140-5p | ABCD3 | miRNA-up_mRNA-down |
| COAD | hsa-miR-140-5p | FAS | miRNA-up_mRNA-down |
| COAD | hsa-miR-140-5p | RIMKLB | miRNA-up_mRNA-down |
| COAD | hsa-miR-140-5p | KCTD12 | miRNA-up_mRNA-down |
| COAD | hsa-miR-140-5p | CHP1 | miRNA-up_mRNA-down |
| COAD | hsa-miR-140-5p | CCNYL1 | miRNA-up_mRNA-down |
| COAD | hsa-miR-140-5p | BMP2 | miRNA-up_mRNA-down |
| COAD | hsa-miR-140-5p | RELL1 | miRNA-up_mRNA-down |
| COAD | hsa-miR-140-5p | EMCN | miRNA-up_mRNA-down |
| COAD | hsa-miR-140-5p | MIER3 | miRNA-up_mRNA-down |
| COAD | hsa-miR-140-5p | STOM | miRNA-up_mRNA-down |
| COAD | hsa-miR-142-3p | TMEM59 | miRNA-up_mRNA-down |
| COAD | hsa-miR-142-3p | MIER1 | miRNA-up_mRNA-down |
| COAD | hsa-miR-142-3p | DNAJB4 | miRNA-up_mRNA-down |
| COAD | hsa-miR-142-3p | PDE8A | miRNA-up_mRNA-down |
| COAD | hsa-miR-142-3p | MGAT4A | miRNA-up_mRNA-down |
| COAD | hsa-miR-142-3p | MAP2 | miRNA-up_mRNA-down |
| COAD | hsa-miR-142-3p | TNFRSF13C | miRNA-up_mRNA-down |
| COAD | hsa-miR-142-3p | LXN | miRNA-up_mRNA-down |
| COAD | hsa-miR-142-3p | RBM47 | miRNA-up_mRNA-down |
| COAD | hsa-miR-142-3p | GAB1 | miRNA-up_mRNA-down |
| COAD | hsa-miR-142-3p | MEF2C | miRNA-up_mRNA-down |
| COAD | hsa-miR-142-3p | NR3C1 | miRNA-up_mRNA-down |
| COAD | hsa-miR-142-3p | ANKRD33B | miRNA-up_mRNA-down |
| COAD | hsa-miR-142-3p | GABRG2 | miRNA-up_mRNA-down |
| COAD | hsa-miR-142-3p | SLC22A23 | miRNA-up_mRNA-down |
| COAD | hsa-miR-142-3p | MARCKS | miRNA-up_mRNA-down |
| COAD | hsa-miR-142-3p | WASL | miRNA-up_mRNA-down |
| COAD | hsa-miR-193a-3p | PADI2 | miRNA-up_mRNA-down |
| COAD | hsa-miR-193a-3p | PLXNA2 | miRNA-up_mRNA-down |
| COAD | hsa-miR-193a-3p | PTPRF | miRNA-up_mRNA-down |
| COAD | hsa-miR-193a-3p | LGR4 | miRNA-up_mRNA-down |
| COAD | hsa-miR-193a-3p | DENND5B | miRNA-up_mRNA-down |
| COAD | hsa-miR-193a-3p | SOS2 | miRNA-up_mRNA-down |
| COAD | hsa-miR-193a-3p | TMEM30B | miRNA-up_mRNA-down |
| COAD | hsa-miR-193a-3p | ADCY9 | miRNA-up_mRNA-down |
| COAD | hsa-miR-193a-3p | MGAT4A | miRNA-up_mRNA-down |
| COAD | hsa-miR-193a-3p | SPECC1L | miRNA-up_mRNA-down |
| COAD | hsa-miR-193a-3p | PLS1 | miRNA-up_mRNA-down |
| COAD | hsa-miR-193a-3p | RHOH | miRNA-up_mRNA-down |
| COAD | hsa-miR-193a-3p | EBF1 | miRNA-up_mRNA-down |
| COAD | hsa-miR-193a-3p | SLC26A2 | miRNA-up_mRNA-down |
| COAD | hsa-miR-22-3p | CAMK2N1 | miRNA-up_mRNA-down |
| COAD | hsa-miR-22-3p | SLC9A1 | miRNA-up_mRNA-down |
| COAD | hsa-miR-22-3p | GNG12 | miRNA-up_mRNA-down |
| COAD | hsa-miR-22-3p | IL6R | miRNA-up_mRNA-down |
| COAD | hsa-miR-22-3p | MARCHF8 | miRNA-up_mRNA-down |
| COAD | hsa-miR-22-3p | FAS | miRNA-up_mRNA-down |
| COAD | hsa-miR-22-3p | VWA5A | miRNA-up_mRNA-down |
| COAD | hsa-miR-22-3p | SLC2A13 | miRNA-up_mRNA-down |
| COAD | hsa-miR-22-3p | ACVRL1 | miRNA-up_mRNA-down |
| COAD | hsa-miR-22-3p | TMEM30B | miRNA-up_mRNA-down |
| COAD | hsa-miR-22-3p | MIDEAS | miRNA-up_mRNA-down |
| COAD | hsa-miR-22-3p | RAPGEFL1 | miRNA-up_mRNA-down |
| COAD | hsa-miR-22-3p | ADAM11 | miRNA-up_mRNA-down |
| COAD | hsa-miR-22-3p | TSHZ1 | miRNA-up_mRNA-down |
| COAD | hsa-miR-22-3p | NTSR1 | miRNA-up_mRNA-down |
| COAD | hsa-miR-22-3p | CBX6 | miRNA-up_mRNA-down |
| COAD | hsa-miR-22-3p | SPECC1L | miRNA-up_mRNA-down |
| COAD | hsa-miR-22-3p | NR3C1 | miRNA-up_mRNA-down |
| COAD | hsa-miR-22-3p | PPARGC1B | miRNA-up_mRNA-down |
| COAD | hsa-miR-22-3p | SLC22A23 | miRNA-up_mRNA-down |
| COAD | hsa-miR-22-3p | LRRC1 | miRNA-up_mRNA-down |
| COAD | hsa-miR-22-3p | ENPP2 | miRNA-up_mRNA-down |
| COAD | hsa-miR-22-3p | SLC6A8 | miRNA-up_mRNA-down |
| COAD | hsa-miR-24-3p | CTSS | miRNA-up_mRNA-down |
| COAD | hsa-miR-24-3p | FBLIM1 | miRNA-up_mRNA-down |
| COAD | hsa-miR-24-3p | PTPRF | miRNA-up_mRNA-down |
| COAD | hsa-miR-24-3p | RAVER2 | miRNA-up_mRNA-down |
| COAD | hsa-miR-24-3p | CDC14A | miRNA-up_mRNA-down |
| COAD | hsa-miR-24-3p | CGN | miRNA-up_mRNA-down |
| COAD | hsa-miR-24-3p | DENND5B | miRNA-up_mRNA-down |
| COAD | hsa-miR-24-3p | APPL2 | miRNA-up_mRNA-down |
| COAD | hsa-miR-24-3p | HRK | miRNA-up_mRNA-down |
| COAD | hsa-miR-24-3p | ETNK1 | miRNA-up_mRNA-down |
| COAD | hsa-miR-24-3p | RPH3A | miRNA-up_mRNA-down |
| COAD | hsa-miR-24-3p | SUSD6 | miRNA-up_mRNA-down |
| COAD | hsa-miR-24-3p | BMF | miRNA-up_mRNA-down |
| COAD | hsa-miR-24-3p | SCAMP2 | miRNA-up_mRNA-down |
| COAD | hsa-miR-24-3p | AP3S2 | miRNA-up_mRNA-down |
| COAD | hsa-miR-24-3p | GNG13 | miRNA-up_mRNA-down |
| COAD | hsa-miR-24-3p | PLCD3 | miRNA-up_mRNA-down |
| COAD | hsa-miR-24-3p | TSHZ1 | miRNA-up_mRNA-down |
| COAD | hsa-miR-24-3p | ADD2 | miRNA-up_mRNA-down |
| COAD | hsa-miR-24-3p | MGAT4A | miRNA-up_mRNA-down |
| COAD | hsa-miR-24-3p | STK17B | miRNA-up_mRNA-down |
| COAD | hsa-miR-24-3p | HK2 | miRNA-up_mRNA-down |
| COAD | hsa-miR-24-3p | BCL2L11 | miRNA-up_mRNA-down |
| COAD | hsa-miR-24-3p | ITM2C | miRNA-up_mRNA-down |
| COAD | hsa-miR-24-3p | PTPRT | miRNA-up_mRNA-down |
| COAD | hsa-miR-24-3p | PEX26 | miRNA-up_mRNA-down |
| COAD | hsa-miR-24-3p | SPECC1L | miRNA-up_mRNA-down |
| COAD | hsa-miR-24-3p | TLL1 | miRNA-up_mRNA-down |
| COAD | hsa-miR-24-3p | SEMA6A | miRNA-up_mRNA-down |
| COAD | hsa-miR-24-3p | SLC26A2 | miRNA-up_mRNA-down |
| COAD | hsa-miR-24-3p | SYNPO | miRNA-up_mRNA-down |
| COAD | hsa-miR-24-3p | SLC22A23 | miRNA-up_mRNA-down |
| COAD | hsa-miR-24-3p | PPARD | miRNA-up_mRNA-down |
| COAD | hsa-miR-24-3p | PTP4A1 | miRNA-up_mRNA-down |
| COAD | hsa-miR-24-3p | KIF13B | miRNA-up_mRNA-down |
| COAD | hsa-miR-24-3p | PI15 | miRNA-up_mRNA-down |
| COAD | hsa-miR-24-3p | ZER1 | miRNA-up_mRNA-down |
| COAD | hsa-miR-24-3p | CRAT | miRNA-up_mRNA-down |
| COAD | hsa-miR-24-3p | SYP | miRNA-up_mRNA-down |
| COAD | hsa-miR-107 | SLC9A1 | miRNA-up_mRNA-down |
| COAD | hsa-miR-107 | GNG12 | miRNA-up_mRNA-down |
| COAD | hsa-miR-107 | IGSF3 | miRNA-up_mRNA-down |
| COAD | hsa-miR-107 | PLXNA2 | miRNA-up_mRNA-down |
| COAD | hsa-miR-107 | SIPA1L2 | miRNA-up_mRNA-down |
| COAD | hsa-miR-107 | DNAJB4 | miRNA-up_mRNA-down |
| COAD | hsa-miR-107 | CDC14A | miRNA-up_mRNA-down |
| COAD | hsa-miR-107 | JCAD | miRNA-up_mRNA-down |
| COAD | hsa-miR-107 | CNNM2 | miRNA-up_mRNA-down |
| COAD | hsa-miR-107 | SMPD1 | miRNA-up_mRNA-down |
| COAD | hsa-miR-107 | ENDOD1 | miRNA-up_mRNA-down |
| COAD | hsa-miR-107 | ZC3H12C | miRNA-up_mRNA-down |
| COAD | hsa-miR-107 | RIMKLB | miRNA-up_mRNA-down |
| COAD | hsa-miR-107 | ETNK1 | miRNA-up_mRNA-down |
| COAD | hsa-miR-107 | SOS2 | miRNA-up_mRNA-down |
| COAD | hsa-miR-107 | MIDEAS | miRNA-up_mRNA-down |
| COAD | hsa-miR-107 | SUSD6 | miRNA-up_mRNA-down |
| COAD | hsa-miR-107 | SLC30A4 | miRNA-up_mRNA-down |
| COAD | hsa-miR-107 | CA12 | miRNA-up_mRNA-down |
| COAD | hsa-miR-107 | NEO1 | miRNA-up_mRNA-down |
| COAD | hsa-miR-107 | LITAF | miRNA-up_mRNA-down |
| COAD | hsa-miR-107 | ACOX1 | miRNA-up_mRNA-down |
| COAD | hsa-miR-107 | RAPGEFL1 | miRNA-up_mRNA-down |
| COAD | hsa-miR-107 | DSEL | miRNA-up_mRNA-down |
| COAD | hsa-miR-107 | CERS1 | miRNA-up_mRNA-down |
| COAD | hsa-miR-107 | CD37 | miRNA-up_mRNA-down |
| COAD | hsa-miR-107 | CYRIA | miRNA-up_mRNA-down |
| COAD | hsa-miR-107 | KCNG3 | miRNA-up_mRNA-down |
| COAD | hsa-miR-107 | MGAT4A | miRNA-up_mRNA-down |
| COAD | hsa-miR-107 | KIF1A | miRNA-up_mRNA-down |
| COAD | hsa-miR-107 | CCNYL1 | miRNA-up_mRNA-down |
| COAD | hsa-miR-107 | ITM2C | miRNA-up_mRNA-down |
| COAD | hsa-miR-107 | ACSS2 | miRNA-up_mRNA-down |
| COAD | hsa-miR-107 | PPP1R16B | miRNA-up_mRNA-down |
| COAD | hsa-miR-107 | CBX6 | miRNA-up_mRNA-down |
| COAD | hsa-miR-107 | TRANK1 | miRNA-up_mRNA-down |
| COAD | hsa-miR-107 | HIGD1A | miRNA-up_mRNA-down |
| COAD | hsa-miR-107 | BTLA | miRNA-up_mRNA-down |
| COAD | hsa-miR-107 | TLL1 | miRNA-up_mRNA-down |
| COAD | hsa-miR-107 | EDIL3 | miRNA-up_mRNA-down |
| COAD | hsa-miR-107 | SEMA6A | miRNA-up_mRNA-down |
| COAD | hsa-miR-107 | SLC26A2 | miRNA-up_mRNA-down |
| COAD | hsa-miR-107 | SLC36A1 | miRNA-up_mRNA-down |
| COAD | hsa-miR-107 | NEURL1B | miRNA-up_mRNA-down |
| COAD | hsa-miR-107 | MBOAT1 | miRNA-up_mRNA-down |
| COAD | hsa-miR-107 | CACNA2D1 | miRNA-up_mRNA-down |
| COAD | hsa-miR-107 | AGFG2 | miRNA-up_mRNA-down |
| COAD | hsa-miR-107 | PAG1 | miRNA-up_mRNA-down |
| COAD | hsa-miR-107 | ENPP2 | miRNA-up_mRNA-down |
| COAD | hsa-miR-107 | MOB3B | miRNA-up_mRNA-down |
| COAD | hsa-miR-107 | SEPTIN6 | miRNA-up_mRNA-down |
| COAD | hsa-miR-27a-3p | SLC9A1 | miRNA-up_mRNA-down |
| COAD | hsa-miR-27a-3p | TMEM59 | miRNA-up_mRNA-down |
| COAD | hsa-miR-27a-3p | GNG12 | miRNA-up_mRNA-down |
| COAD | hsa-miR-27a-3p | IGSF3 | miRNA-up_mRNA-down |
| COAD | hsa-miR-27a-3p | SESN2 | miRNA-up_mRNA-down |
| COAD | hsa-miR-27a-3p | TSPAN1 | miRNA-up_mRNA-down |
| COAD | hsa-miR-27a-3p | ST6GALNAC3 | miRNA-up_mRNA-down |
| COAD | hsa-miR-27a-3p | ABCD3 | miRNA-up_mRNA-down |
| COAD | hsa-miR-27a-3p | CSF1 | miRNA-up_mRNA-down |
| COAD | hsa-miR-27a-3p | MAGI3 | miRNA-up_mRNA-down |
| COAD | hsa-miR-27a-3p | NR5A2 | miRNA-up_mRNA-down |
| COAD | hsa-miR-27a-3p | SERTAD4 | miRNA-up_mRNA-down |
| COAD | hsa-miR-27a-3p | FAS | miRNA-up_mRNA-down |
| COAD | hsa-miR-27a-3p | LGR4 | miRNA-up_mRNA-down |
| COAD | hsa-miR-27a-3p | STYK1 | miRNA-up_mRNA-down |
| COAD | hsa-miR-27a-3p | DENND5B | miRNA-up_mRNA-down |
| COAD | hsa-miR-27a-3p | LIMA1 | miRNA-up_mRNA-down |
| COAD | hsa-miR-27a-3p | KITLG | miRNA-up_mRNA-down |
| COAD | hsa-miR-27a-3p | KCTD12 | miRNA-up_mRNA-down |
| COAD | hsa-miR-27a-3p | SUSD6 | miRNA-up_mRNA-down |
| COAD | hsa-miR-27a-3p | FLRT2 | miRNA-up_mRNA-down |
| COAD | hsa-miR-27a-3p | MEIS2 | miRNA-up_mRNA-down |
| COAD | hsa-miR-27a-3p | TMOD2 | miRNA-up_mRNA-down |
| COAD | hsa-miR-27a-3p | NEO1 | miRNA-up_mRNA-down |
| COAD | hsa-miR-27a-3p | PDE8A | miRNA-up_mRNA-down |
| COAD | hsa-miR-27a-3p | SV2B | miRNA-up_mRNA-down |
| COAD | hsa-miR-27a-3p | GNG13 | miRNA-up_mRNA-down |
| COAD | hsa-miR-27a-3p | CDIP1 | miRNA-up_mRNA-down |
| COAD | hsa-miR-27a-3p | LITAF | miRNA-up_mRNA-down |
| COAD | hsa-miR-27a-3p | RBFOX1 | miRNA-up_mRNA-down |
| COAD | hsa-miR-27a-3p | FIGN | miRNA-up_mRNA-down |
| COAD | hsa-miR-27a-3p | EPAS1 | miRNA-up_mRNA-down |
| COAD | hsa-miR-27a-3p | BCL2L11 | miRNA-up_mRNA-down |
| COAD | hsa-miR-27a-3p | MAP2 | miRNA-up_mRNA-down |
| COAD | hsa-miR-27a-3p | UNC80 | miRNA-up_mRNA-down |
| COAD | hsa-miR-27a-3p | PTPRT | miRNA-up_mRNA-down |
| COAD | hsa-miR-27a-3p | SNAP25 | miRNA-up_mRNA-down |
| COAD | hsa-miR-27a-3p | HCLS1 | miRNA-up_mRNA-down |
| COAD | hsa-miR-27a-3p | PLCL2 | miRNA-up_mRNA-down |
| COAD | hsa-miR-27a-3p | ABHD5 | miRNA-up_mRNA-down |
| COAD | hsa-miR-27a-3p | STBD1 | miRNA-up_mRNA-down |
| COAD | hsa-miR-27a-3p | GAB1 | miRNA-up_mRNA-down |
| COAD | hsa-miR-27a-3p | MIER3 | miRNA-up_mRNA-down |
| COAD | hsa-miR-27a-3p | EDIL3 | miRNA-up_mRNA-down |
| COAD | hsa-miR-27a-3p | MEF2C | miRNA-up_mRNA-down |
| COAD | hsa-miR-27a-3p | PJA2 | miRNA-up_mRNA-down |
| COAD | hsa-miR-27a-3p | SEMA6A | miRNA-up_mRNA-down |
| COAD | hsa-miR-27a-3p | PTGER4 | miRNA-up_mRNA-down |
| COAD | hsa-miR-27a-3p | PPARGC1B | miRNA-up_mRNA-down |
| COAD | hsa-miR-27a-3p | NEURL1B | miRNA-up_mRNA-down |
| COAD | hsa-miR-27a-3p | SLC22A23 | miRNA-up_mRNA-down |
| COAD | hsa-miR-27a-3p | TRDN | miRNA-up_mRNA-down |
| COAD | hsa-miR-27a-3p | LRRC1 | miRNA-up_mRNA-down |
| COAD | hsa-miR-27a-3p | MARCKS | miRNA-up_mRNA-down |
| COAD | hsa-miR-27a-3p | WASL | miRNA-up_mRNA-down |
| COAD | hsa-miR-27a-3p | PI15 | miRNA-up_mRNA-down |
| COAD | hsa-miR-27a-3p | RALYL | miRNA-up_mRNA-down |
| COAD | hsa-miR-27a-3p | GOLM1 | miRNA-up_mRNA-down |
| COAD | hsa-miR-27a-3p | GRIN3A | miRNA-up_mRNA-down |
| COAD | hsa-miR-27a-3p | STOM | miRNA-up_mRNA-down |
| COAD | hsa-miR-27a-3p | RXRA | miRNA-up_mRNA-down |
| COAD | hsa-miR-27a-3p | SYP | miRNA-up_mRNA-down |
| COAD | hsa-miR-27a-3p | RAB39B | miRNA-up_mRNA-down |
| COAD | hsa-miR-27a-3p | NLGN4Y | miRNA-up_mRNA-down |
| COAD | hsa-miR-455-5p | GNG12 | miRNA-up_mRNA-down |
| COAD | hsa-miR-455-5p | MIER1 | miRNA-up_mRNA-down |
| COAD | hsa-miR-455-5p | MS4A1 | miRNA-up_mRNA-down |
| COAD | hsa-miR-455-5p | HCFC2 | miRNA-up_mRNA-down |
| COAD | hsa-miR-455-5p | KCTD12 | miRNA-up_mRNA-down |
| COAD | hsa-miR-455-5p | TMOD2 | miRNA-up_mRNA-down |
| COAD | hsa-miR-455-5p | OSBPL1A | miRNA-up_mRNA-down |
| COAD | hsa-miR-455-5p | FIGN | miRNA-up_mRNA-down |
| COAD | hsa-miR-455-5p | DOCK10 | miRNA-up_mRNA-down |
| COAD | hsa-miR-455-5p | EPAS1 | miRNA-up_mRNA-down |
| COAD | hsa-miR-455-5p | CSF2RB | miRNA-up_mRNA-down |
| COAD | hsa-miR-455-5p | MGLL | miRNA-up_mRNA-down |
| COAD | hsa-miR-455-5p | MIER3 | miRNA-up_mRNA-down |
| COAD | hsa-miR-455-5p | PJA2 | miRNA-up_mRNA-down |
| COAD | hsa-miR-455-5p | MARCKS | miRNA-up_mRNA-down |
| COAD | hsa-miR-455-5p | JAZF1 | miRNA-up_mRNA-down |
| COAD | hsa-miR-455-5p | PI15 | miRNA-up_mRNA-down |
| COAD | hsa-miR-455-5p | SLC6A8 | miRNA-up_mRNA-down |
| COAD | hsa-miR-429 | GNG12 | miRNA-up_mRNA-down |
| COAD | hsa-miR-429 | PLXNA2 | miRNA-up_mRNA-down |
| COAD | hsa-miR-429 | MIER1 | miRNA-up_mRNA-down |
| COAD | hsa-miR-429 | CDC14A | miRNA-up_mRNA-down |
| COAD | hsa-miR-429 | NR5A2 | miRNA-up_mRNA-down |
| COAD | hsa-miR-429 | JCAD | miRNA-up_mRNA-down |
| COAD | hsa-miR-429 | FGFR2 | miRNA-up_mRNA-down |
| COAD | hsa-miR-429 | LMO2 | miRNA-up_mRNA-down |
| COAD | hsa-miR-429 | ZC3H12C | miRNA-up_mRNA-down |
| COAD | hsa-miR-429 | FLI1 | miRNA-up_mRNA-down |
| COAD | hsa-miR-429 | DENND5B | miRNA-up_mRNA-down |
| COAD | hsa-miR-429 | RIMKLB | miRNA-up_mRNA-down |
| COAD | hsa-miR-429 | ETNK1 | miRNA-up_mRNA-down |
| COAD | hsa-miR-429 | KCTD12 | miRNA-up_mRNA-down |
| COAD | hsa-miR-429 | GNG2 | miRNA-up_mRNA-down |
| COAD | hsa-miR-429 | BDKRB2 | miRNA-up_mRNA-down |
| COAD | hsa-miR-429 | TMOD2 | miRNA-up_mRNA-down |
| COAD | hsa-miR-429 | NEO1 | miRNA-up_mRNA-down |
| COAD | hsa-miR-429 | ADCY9 | miRNA-up_mRNA-down |
| COAD | hsa-miR-429 | ABHD3 | miRNA-up_mRNA-down |
| COAD | hsa-miR-429 | BCL2 | miRNA-up_mRNA-down |
| COAD | hsa-miR-429 | MGAT4A | miRNA-up_mRNA-down |
| COAD | hsa-miR-429 | FIGN | miRNA-up_mRNA-down |
| COAD | hsa-miR-429 | STK17B | miRNA-up_mRNA-down |
| COAD | hsa-miR-429 | EPAS1 | miRNA-up_mRNA-down |
| COAD | hsa-miR-429 | CCNYL1 | miRNA-up_mRNA-down |
| COAD | hsa-miR-429 | MAP2 | miRNA-up_mRNA-down |
| COAD | hsa-miR-429 | SNAP25 | miRNA-up_mRNA-down |
| COAD | hsa-miR-429 | PPP1R16B | miRNA-up_mRNA-down |
| COAD | hsa-miR-429 | SUSD5 | miRNA-up_mRNA-down |
| COAD | hsa-miR-429 | STAC | miRNA-up_mRNA-down |
| COAD | hsa-miR-429 | GPR27 | miRNA-up_mRNA-down |
| COAD | hsa-miR-429 | NKX3-2 | miRNA-up_mRNA-down |
| COAD | hsa-miR-429 | RELL1 | miRNA-up_mRNA-down |
| COAD | hsa-miR-429 | RBM47 | miRNA-up_mRNA-down |
| COAD | hsa-miR-429 | EMCN | miRNA-up_mRNA-down |
| COAD | hsa-miR-429 | GAB1 | miRNA-up_mRNA-down |
| COAD | hsa-miR-429 | GUCY1A1 | miRNA-up_mRNA-down |
| COAD | hsa-miR-429 | CTNND2 | miRNA-up_mRNA-down |
| COAD | hsa-miR-429 | MIER3 | miRNA-up_mRNA-down |
| COAD | hsa-miR-429 | EFNA5 | miRNA-up_mRNA-down |
| COAD | hsa-miR-429 | PJA2 | miRNA-up_mRNA-down |
| COAD | hsa-miR-429 | NR3C1 | miRNA-up_mRNA-down |
| COAD | hsa-miR-429 | PTGER4 | miRNA-up_mRNA-down |
| COAD | hsa-miR-429 | SLC36A1 | miRNA-up_mRNA-down |
| COAD | hsa-miR-429 | PTP4A1 | miRNA-up_mRNA-down |
| COAD | hsa-miR-429 | MARCKS | miRNA-up_mRNA-down |
| COAD | hsa-miR-429 | JAZF1 | miRNA-up_mRNA-down |
| COAD | hsa-miR-429 | GLI3 | miRNA-up_mRNA-down |
| COAD | hsa-miR-429 | CASD1 | miRNA-up_mRNA-down |
| COAD | hsa-miR-429 | PAG1 | miRNA-up_mRNA-down |
| COAD | hsa-miR-429 | PI15 | miRNA-up_mRNA-down |
| COAD | hsa-miR-429 | DIRAS2 | miRNA-up_mRNA-down |
| COAD | hsa-miR-429 | MTM1 | miRNA-up_mRNA-down |
| COAD | hsa-miR-590-5p | GNG12 | miRNA-up_mRNA-down |
| COAD | hsa-miR-590-5p | RNASEL | miRNA-up_mRNA-down |
| COAD | hsa-miR-590-5p | ABCD3 | miRNA-up_mRNA-down |
| COAD | hsa-miR-590-5p | IL6R | miRNA-up_mRNA-down |
| COAD | hsa-miR-590-5p | CAPN2 | miRNA-up_mRNA-down |
| COAD | hsa-miR-590-5p | STYK1 | miRNA-up_mRNA-down |
| COAD | hsa-miR-590-5p | SLC2A13 | miRNA-up_mRNA-down |
| COAD | hsa-miR-590-5p | LIMA1 | miRNA-up_mRNA-down |
| COAD | hsa-miR-590-5p | ETNK1 | miRNA-up_mRNA-down |
| COAD | hsa-miR-590-5p | SOS2 | miRNA-up_mRNA-down |
| COAD | hsa-miR-590-5p | GNG2 | miRNA-up_mRNA-down |
| COAD | hsa-miR-590-5p | CCR7 | miRNA-up_mRNA-down |
| COAD | hsa-miR-590-5p | WWTR1 | miRNA-up_mRNA-down |
| COAD | hsa-miR-590-5p | MEF2C | miRNA-up_mRNA-down |
| COAD | hsa-miR-590-5p | PJA2 | miRNA-up_mRNA-down |
| COAD | hsa-miR-590-5p | ZSWIM6 | miRNA-up_mRNA-down |
| COAD | hsa-miR-590-5p | TDP2 | miRNA-up_mRNA-down |
| COAD | hsa-miR-590-5p | SAMD9 | miRNA-up_mRNA-down |
| COAD | hsa-miR-590-5p | PI15 | miRNA-up_mRNA-down |
| COAD | hsa-miR-10a-5p | CDC14A | miRNA-up_mRNA-down |
| COAD | hsa-miR-10a-5p | RCSD1 | miRNA-up_mRNA-down |
| COAD | hsa-miR-10a-5p | PRDX6 | miRNA-up_mRNA-down |
| COAD | hsa-miR-10a-5p | NR5A2 | miRNA-up_mRNA-down |
| COAD | hsa-miR-10a-5p | JCAD | miRNA-up_mRNA-down |
| COAD | hsa-miR-10a-5p | SIAE | miRNA-up_mRNA-down |
| COAD | hsa-miR-10a-5p | ZC3H12C | miRNA-up_mRNA-down |
| COAD | hsa-miR-10a-5p | APPL2 | miRNA-up_mRNA-down |
| COAD | hsa-miR-10a-5p | SUSD6 | miRNA-up_mRNA-down |
| COAD | hsa-miR-10a-5p | FLRT2 | miRNA-up_mRNA-down |
| COAD | hsa-miR-10a-5p | SLC30A4 | miRNA-up_mRNA-down |
| COAD | hsa-miR-10a-5p | ABHD3 | miRNA-up_mRNA-down |
| COAD | hsa-miR-10a-5p | TFCP2L1 | miRNA-up_mRNA-down |
| COAD | hsa-miR-10a-5p | FIGN | miRNA-up_mRNA-down |
| COAD | hsa-miR-10a-5p | KCNS3 | miRNA-up_mRNA-down |
| COAD | hsa-miR-10a-5p | EPAS1 | miRNA-up_mRNA-down |
| COAD | hsa-miR-10a-5p | CNNM4 | miRNA-up_mRNA-down |
| COAD | hsa-miR-10a-5p | BCL2L11 | miRNA-up_mRNA-down |
| COAD | hsa-miR-10a-5p | SYNPO | miRNA-up_mRNA-down |
| COAD | hsa-miR-10a-5p | MARCKS | miRNA-up_mRNA-down |
| COAD | hsa-miR-10a-5p | MYCT1 | miRNA-up_mRNA-down |
| COAD | hsa-miR-10a-5p | GIMAP8 | miRNA-up_mRNA-down |
| COAD | hsa-miR-10a-5p | RXRA | miRNA-up_mRNA-down |
| COAD | hsa-miR-10a-5p | SYP | miRNA-up_mRNA-down |

**Table S3**. Semi-shared ceRNA network nodes.

| node1 | node2 | line |
| --- | --- | --- |
| IGF2-AS | hsa-miR-125a-5p | lncRNA-up_miRNA-down |
| LINC00525 | hsa-miR-125a-5p | lncRNA-up_miRNA-down |
| GOLGA8IP | hsa-miR-125a-5p | lncRNA-up_miRNA-down |
| C3P1 | hsa-miR-125a-5p | lncRNA-up_miRNA-down |
| PRSS30P | hsa-miR-125a-5p | lncRNA-up_miRNA-down |
| DLEU1 | hsa-miR-125a-5p | lncRNA-up_miRNA-down |
| HECW1-IT1 | hsa-miR-125a-5p | lncRNA-up_miRNA-down |
| CCNYL2 | hsa-miR-125a-5p | lncRNA-up_miRNA-down |
| WT1-AS | hsa-miR-125a-5p | lncRNA-up_miRNA-down |
| DSCR4 | hsa-miR-125a-5p | lncRNA-up_miRNA-down |
| TMEM105 | hsa-miR-125a-5p | lncRNA-up_miRNA-down |
| LINC00523 | hsa-miR-125a-5p | lncRNA-up_miRNA-down |
| C9orf163 | hsa-miR-125a-5p | lncRNA-up_miRNA-down |
| LINC00173 | hsa-miR-125a-5p | lncRNA-up_miRNA-down |
| KRT42P | hsa-miR-125a-5p | lncRNA-up_miRNA-down |
| SLC6A10P | hsa-miR-125a-5p | lncRNA-up_miRNA-down |
| CLDN10-AS1 | hsa-miR-125a-5p | lncRNA-up_miRNA-down |
| LGALS8-AS1 | hsa-miR-125a-5p | lncRNA-up_miRNA-down |
| RUSC1-AS1 | hsa-miR-125a-5p | lncRNA-up_miRNA-down |
| ST7-AS2 | hsa-miR-125a-5p | lncRNA-up_miRNA-down |
| LINC00343 | hsa-miR-125a-5p | lncRNA-up_miRNA-down |
| NAALADL2-AS2 | hsa-miR-125a-5p | lncRNA-up_miRNA-down |
| SOX21-AS1 | hsa-miR-125a-5p | lncRNA-up_miRNA-down |
| LINC00200 | hsa-miR-125a-5p | lncRNA-up_miRNA-down |
| UBE2Q1-AS1 | hsa-miR-125a-5p | lncRNA-up_miRNA-down |
| HAR1B | hsa-miR-125a-5p | lncRNA-up_miRNA-down |
| LINC00279 | hsa-miR-125a-5p | lncRNA-up_miRNA-down |
| CASK-AS1 | hsa-miR-125a-5p | lncRNA-up_miRNA-down |
| MYCNOS | hsa-miR-125a-5p | lncRNA-up_miRNA-down |
| ZNRF3-IT1 | hsa-miR-125a-5p | lncRNA-up_miRNA-down |
| EGOT | hsa-miR-125a-5p | lncRNA-up_miRNA-down |
| MYO16-AS1 | hsa-miR-125a-5p | lncRNA-up_miRNA-down |
| DGCR5 | hsa-miR-125a-5p | lncRNA-up_miRNA-down |
| HOTTIP | hsa-miR-125a-5p | lncRNA-up_miRNA-down |
| HAS2-AS1 | hsa-miR-125a-5p | lncRNA-up_miRNA-down |
| ARHGEF38-IT1 | hsa-miR-125a-5p | lncRNA-up_miRNA-down |
| AACSP1 | hsa-miR-125a-5p | lncRNA-up_miRNA-down |
| KCNQ1OT1 | hsa-miR-125a-5p | lncRNA-up_miRNA-down |
| IGF2-AS | hsa-miR-125b-5p | lncRNA-up_miRNA-down |
| LINC00525 | hsa-miR-125b-5p | lncRNA-up_miRNA-down |
| GOLGA8IP | hsa-miR-125b-5p | lncRNA-up_miRNA-down |
| C3P1 | hsa-miR-125b-5p | lncRNA-up_miRNA-down |
| PRSS30P | hsa-miR-125b-5p | lncRNA-up_miRNA-down |
| DLEU1 | hsa-miR-125b-5p | lncRNA-up_miRNA-down |
| HECW1-IT1 | hsa-miR-125b-5p | lncRNA-up_miRNA-down |
| CCNYL2 | hsa-miR-125b-5p | lncRNA-up_miRNA-down |
| WT1-AS | hsa-miR-125b-5p | lncRNA-up_miRNA-down |
| DSCR4 | hsa-miR-125b-5p | lncRNA-up_miRNA-down |
| TMEM105 | hsa-miR-125b-5p | lncRNA-up_miRNA-down |
| LINC00523 | hsa-miR-125b-5p | lncRNA-up_miRNA-down |
| C9orf163 | hsa-miR-125b-5p | lncRNA-up_miRNA-down |
| LINC00173 | hsa-miR-125b-5p | lncRNA-up_miRNA-down |
| KRT42P | hsa-miR-125b-5p | lncRNA-up_miRNA-down |
| SLC6A10P | hsa-miR-125b-5p | lncRNA-up_miRNA-down |
| CLDN10-AS1 | hsa-miR-125b-5p | lncRNA-up_miRNA-down |
| LGALS8-AS1 | hsa-miR-125b-5p | lncRNA-up_miRNA-down |
| RUSC1-AS1 | hsa-miR-125b-5p | lncRNA-up_miRNA-down |
| ST7-AS2 | hsa-miR-125b-5p | lncRNA-up_miRNA-down |
| LINC00343 | hsa-miR-125b-5p | lncRNA-up_miRNA-down |
| NAALADL2-AS2 | hsa-miR-125b-5p | lncRNA-up_miRNA-down |
| SOX21-AS1 | hsa-miR-125b-5p | lncRNA-up_miRNA-down |
| LINC00200 | hsa-miR-125b-5p | lncRNA-up_miRNA-down |
| UBE2Q1-AS1 | hsa-miR-125b-5p | lncRNA-up_miRNA-down |
| HAR1B | hsa-miR-125b-5p | lncRNA-up_miRNA-down |
| LINC00279 | hsa-miR-125b-5p | lncRNA-up_miRNA-down |
| CASK-AS1 | hsa-miR-125b-5p | lncRNA-up_miRNA-down |
| MYCNOS | hsa-miR-125b-5p | lncRNA-up_miRNA-down |
| ZNRF3-IT1 | hsa-miR-125b-5p | lncRNA-up_miRNA-down |
| EGOT | hsa-miR-125b-5p | lncRNA-up_miRNA-down |
| MYO16-AS1 | hsa-miR-125b-5p | lncRNA-up_miRNA-down |
| DGCR5 | hsa-miR-125b-5p | lncRNA-up_miRNA-down |
| HOTTIP | hsa-miR-125b-5p | lncRNA-up_miRNA-down |
| HAS2-AS1 | hsa-miR-125b-5p | lncRNA-up_miRNA-down |
| ARHGEF38-IT1 | hsa-miR-125b-5p | lncRNA-up_miRNA-down |
| AACSP1 | hsa-miR-125b-5p | lncRNA-up_miRNA-down |
| KCNQ1OT1 | hsa-miR-125b-5p | lncRNA-up_miRNA-down |
| NSUN5P2 | hsa-miR-129-5p | lncRNA-up_miRNA-down |
| LINC00470 | hsa-miR-129-5p | lncRNA-up_miRNA-down |
| DLEU1 | hsa-miR-129-5p | lncRNA-up_miRNA-down |
| TMEM78 | hsa-miR-129-5p | lncRNA-up_miRNA-down |
| CCNYL2 | hsa-miR-129-5p | lncRNA-up_miRNA-down |
| WT1-AS | hsa-miR-129-5p | lncRNA-up_miRNA-down |
| DSCR4 | hsa-miR-129-5p | lncRNA-up_miRNA-down |
| LINC00221 | hsa-miR-129-5p | lncRNA-up_miRNA-down |
| LINC00523 | hsa-miR-129-5p | lncRNA-up_miRNA-down |
| SNHG12 | hsa-miR-129-5p | lncRNA-up_miRNA-down |
| LINC00501 | hsa-miR-129-5p | lncRNA-up_miRNA-down |
| CLLU1 | hsa-miR-129-5p | lncRNA-up_miRNA-down |
| UCA1 | hsa-miR-129-5p | lncRNA-up_miRNA-down |
| C10orf55 | hsa-miR-129-5p | lncRNA-up_miRNA-down |
| NSUN5P1 | hsa-miR-129-5p | lncRNA-up_miRNA-down |
| LINC00466 | hsa-miR-129-5p | lncRNA-up_miRNA-down |
| LINC00393 | hsa-miR-129-5p | lncRNA-up_miRNA-down |
| ST7-AS2 | hsa-miR-129-5p | lncRNA-up_miRNA-down |
| ZMYM4-AS1 | hsa-miR-129-5p | lncRNA-up_miRNA-down |
| SOX21-AS1 | hsa-miR-129-5p | lncRNA-up_miRNA-down |
| MACROD2-IT1 | hsa-miR-129-5p | lncRNA-up_miRNA-down |
| GK-IT1 | hsa-miR-129-5p | lncRNA-up_miRNA-down |
| KIF25-AS1 | hsa-miR-129-5p | lncRNA-up_miRNA-down |
| ALMS1-IT1 | hsa-miR-129-5p | lncRNA-up_miRNA-down |
| DLEU2 | hsa-miR-129-5p | lncRNA-up_miRNA-down |
| BPESC1 | hsa-miR-129-5p | lncRNA-up_miRNA-down |
| ATP11A-AS1 | hsa-miR-129-5p | lncRNA-up_miRNA-down |
| DSCR10 | hsa-miR-129-5p | lncRNA-up_miRNA-down |
| MYCNOS | hsa-miR-129-5p | lncRNA-up_miRNA-down |
| TM4SF19-AS1 | hsa-miR-129-5p | lncRNA-up_miRNA-down |
| EGOT | hsa-miR-129-5p | lncRNA-up_miRNA-down |
| MYO16-AS1 | hsa-miR-129-5p | lncRNA-up_miRNA-down |
| MYB-AS1 | hsa-miR-129-5p | lncRNA-up_miRNA-down |
| DGCR5 | hsa-miR-129-5p | lncRNA-up_miRNA-down |
| LSAMP-AS1 | hsa-miR-129-5p | lncRNA-up_miRNA-down |
| SNHG3 | hsa-miR-129-5p | lncRNA-up_miRNA-down |
| CSAG4 | hsa-miR-129-5p | lncRNA-up_miRNA-down |
| HOTTIP | hsa-miR-129-5p | lncRNA-up_miRNA-down |
| CRNDE | hsa-miR-129-5p | lncRNA-up_miRNA-down |
| HAS2-AS1 | hsa-miR-129-5p | lncRNA-up_miRNA-down |
| AACSP1 | hsa-miR-129-5p | lncRNA-up_miRNA-down |
| PCAT1 | hsa-miR-129-5p | lncRNA-up_miRNA-down |
| SNHG1 | hsa-miR-129-5p | lncRNA-up_miRNA-down |
| KCNQ1OT1 | hsa-miR-129-5p | lncRNA-up_miRNA-down |
| GOLGA8IP | hsa-miR-363-3p | lncRNA-up_miRNA-down |
| DLEU1 | hsa-miR-363-3p | lncRNA-up_miRNA-down |
| TMEM78 | hsa-miR-363-3p | lncRNA-up_miRNA-down |
| CCNYL2 | hsa-miR-363-3p | lncRNA-up_miRNA-down |
| WT1-AS | hsa-miR-363-3p | lncRNA-up_miRNA-down |
| LINC00221 | hsa-miR-363-3p | lncRNA-up_miRNA-down |
| C2orf27A | hsa-miR-363-3p | lncRNA-up_miRNA-down |
| LINC00501 | hsa-miR-363-3p | lncRNA-up_miRNA-down |
| CLLU1 | hsa-miR-363-3p | lncRNA-up_miRNA-down |
| KRT42P | hsa-miR-363-3p | lncRNA-up_miRNA-down |
| SLC6A10P | hsa-miR-363-3p | lncRNA-up_miRNA-down |
| CLDN10-AS1 | hsa-miR-363-3p | lncRNA-up_miRNA-down |
| LINC00466 | hsa-miR-363-3p | lncRNA-up_miRNA-down |
| RUSC1-AS1 | hsa-miR-363-3p | lncRNA-up_miRNA-down |
| LINC00323 | hsa-miR-363-3p | lncRNA-up_miRNA-down |
| LINC00392 | hsa-miR-363-3p | lncRNA-up_miRNA-down |
| TTC3-AS1 | hsa-miR-363-3p | lncRNA-up_miRNA-down |
| KIF25-AS1 | hsa-miR-363-3p | lncRNA-up_miRNA-down |
| PRRT3-AS1 | hsa-miR-363-3p | lncRNA-up_miRNA-down |
| DLEU2 | hsa-miR-363-3p | lncRNA-up_miRNA-down |
| EMBP1 | hsa-miR-363-3p | lncRNA-up_miRNA-down |
| LINC00462 | hsa-miR-363-3p | lncRNA-up_miRNA-down |
| GAS6-AS1 | hsa-miR-363-3p | lncRNA-up_miRNA-down |
| MYCNOS | hsa-miR-363-3p | lncRNA-up_miRNA-down |
| CRNDE | hsa-miR-363-3p | lncRNA-up_miRNA-down |
| PCAT1 | hsa-miR-363-3p | lncRNA-up_miRNA-down |
| SNHG1 | hsa-miR-363-3p | lncRNA-up_miRNA-down |
| LINC00485 | hsa-miR-363-3p | lncRNA-up_miRNA-down |
| MEG8 | hsa-miR-363-3p | lncRNA-up_miRNA-down |
| KCNQ1OT1 | hsa-miR-363-3p | lncRNA-up_miRNA-down |
| hsa-miR-363-3p | ANP32E | miRNA-down_mRNA-up |
| hsa-miR-363-3p | DUSP10 | miRNA-down_mRNA-up |
| hsa-miR-363-3p | PDPN | miRNA-down_mRNA-up |
| hsa-miR-363-3p | PLPPR4 | miRNA-down_mRNA-up |
| hsa-miR-363-3p | PROX1 | miRNA-down_mRNA-up |
| hsa-miR-363-3p | COX20 | miRNA-down_mRNA-up |
| hsa-miR-363-3p | DKK1 | miRNA-down_mRNA-up |
| hsa-miR-363-3p | ZNF365 | miRNA-down_mRNA-up |
| hsa-miR-363-3p | HMX3 | miRNA-down_mRNA-up |
| hsa-miR-363-3p | MYEOV | miRNA-down_mRNA-up |
| hsa-miR-363-3p | BCAT1 | miRNA-down_mRNA-up |
| hsa-miR-363-3p | PPM1H | miRNA-down_mRNA-up |
| hsa-miR-363-3p | HOXC8 | miRNA-down_mRNA-up |
| hsa-miR-363-3p | FZD10 | miRNA-down_mRNA-up |
| hsa-miR-363-3p | FOXG1 | miRNA-down_mRNA-up |
| hsa-miR-363-3p | PAX9 | miRNA-down_mRNA-up |
| hsa-miR-363-3p | TTC9 | miRNA-down_mRNA-up |
| hsa-miR-363-3p | GOLGA8A | miRNA-down_mRNA-up |
| hsa-miR-363-3p | GOLGA8B | miRNA-down_mRNA-up |
| hsa-miR-363-3p | WDR76 | miRNA-down_mRNA-up |
| hsa-miR-363-3p | RBFOX1 | miRNA-down_mRNA-up |
| hsa-miR-363-3p | CBFB | miRNA-down_mRNA-up |
| hsa-miR-363-3p | ZNF469 | miRNA-down_mRNA-up |
| hsa-miR-363-3p | HOXB8 | miRNA-down_mRNA-up |
| hsa-miR-363-3p | TGIF1 | miRNA-down_mRNA-up |
| hsa-miR-363-3p | ONECUT2 | miRNA-down_mRNA-up |
| hsa-miR-363-3p | HCN2 | miRNA-down_mRNA-up |
| hsa-miR-363-3p | BCL11A | miRNA-down_mRNA-up |
| hsa-miR-363-3p | FBXO41 | miRNA-down_mRNA-up |
| hsa-miR-363-3p | SOX11 | miRNA-down_mRNA-up |
| hsa-miR-363-3p | PKDCC | miRNA-down_mRNA-up |
| hsa-miR-363-3p | MTHFD2 | miRNA-down_mRNA-up |
| hsa-miR-363-3p | SCN2A | miRNA-down_mRNA-up |
| hsa-miR-363-3p | HOXD1 | miRNA-down_mRNA-up |
| hsa-miR-363-3p | MYO1B | miRNA-down_mRNA-up |
| hsa-miR-363-3p | SRXN1 | miRNA-down_mRNA-up |
| hsa-miR-363-3p | CBLN4 | miRNA-down_mRNA-up |
| hsa-miR-363-3p | PMEPA1 | miRNA-down_mRNA-up |
| hsa-miR-363-3p | INSM1 | miRNA-down_mRNA-up |
| hsa-miR-363-3p | SYNDIG1 | miRNA-down_mRNA-up |
| hsa-miR-363-3p | SNAI1 | miRNA-down_mRNA-up |
| hsa-miR-363-3p | WNT5A | miRNA-down_mRNA-up |
| hsa-miR-363-3p | ROBO2 | miRNA-down_mRNA-up |
| hsa-miR-363-3p | CPLX1 | miRNA-down_mRNA-up |
| hsa-miR-363-3p | PPAT | miRNA-down_mRNA-up |
| hsa-miR-363-3p | SLC7A11 | miRNA-down_mRNA-up |
| hsa-miR-363-3p | C4orf46 | miRNA-down_mRNA-up |
| hsa-miR-363-3p | KLHL31 | miRNA-down_mRNA-up |
| hsa-miR-363-3p | COL12A1 | miRNA-down_mRNA-up |
| hsa-miR-363-3p | E2F3 | miRNA-down_mRNA-up |
| hsa-miR-363-3p | SOX4 | miRNA-down_mRNA-up |
| hsa-miR-363-3p | HOXA9 | miRNA-down_mRNA-up |
| hsa-miR-363-3p | IGFBP3 | miRNA-down_mRNA-up |
| hsa-miR-363-3p | SNX10 | miRNA-down_mRNA-up |
| hsa-miR-363-3p | CCT6A | miRNA-down_mRNA-up |
| hsa-miR-363-3p | CSMD1 | miRNA-down_mRNA-up |
| hsa-miR-363-3p | TNFRSF10B | miRNA-down_mRNA-up |
| hsa-miR-363-3p | CCNE2 | miRNA-down_mRNA-up |
| hsa-miR-363-3p | SLC25A32 | miRNA-down_mRNA-up |
| hsa-miR-363-3p | PI15 | miRNA-down_mRNA-up |
| hsa-miR-363-3p | FZD6 | miRNA-down_mRNA-up |
| hsa-miR-363-3p | SQLE | miRNA-down_mRNA-up |
| hsa-miR-363-3p | COL27A1 | miRNA-down_mRNA-up |
| hsa-miR-363-3p | CLCN5 | miRNA-down_mRNA-up |
| hsa-miR-129-5p | RAB3B | miRNA-down_mRNA-up |
| hsa-miR-129-5p | RGS16 | miRNA-down_mRNA-up |
| hsa-miR-129-5p | DUSP10 | miRNA-down_mRNA-up |
| hsa-miR-129-5p | AGT | miRNA-down_mRNA-up |
| hsa-miR-129-5p | CHML | miRNA-down_mRNA-up |
| hsa-miR-129-5p | SYT14 | miRNA-down_mRNA-up |
| hsa-miR-129-5p | VASH2 | miRNA-down_mRNA-up |
| hsa-miR-129-5p | DDX21 | miRNA-down_mRNA-up |
| hsa-miR-129-5p | BDNF | miRNA-down_mRNA-up |
| hsa-miR-129-5p | SYT13 | miRNA-down_mRNA-up |
| hsa-miR-129-5p | PGM2L1 | miRNA-down_mRNA-up |
| hsa-miR-129-5p | CCND1 | miRNA-down_mRNA-up |
| hsa-miR-129-5p | P2RY6 | miRNA-down_mRNA-up |
| hsa-miR-129-5p | BICD1 | miRNA-down_mRNA-up |
| hsa-miR-129-5p | SLC7A1 | miRNA-down_mRNA-up |
| hsa-miR-129-5p | HSPH1 | miRNA-down_mRNA-up |
| hsa-miR-129-5p | POU4F1 | miRNA-down_mRNA-up |
| hsa-miR-129-5p | COL4A1 | miRNA-down_mRNA-up |
| hsa-miR-129-5p | TNFSF11 | miRNA-down_mRNA-up |
| hsa-miR-129-5p | PCDH17 | miRNA-down_mRNA-up |
| hsa-miR-129-5p | ZIC2 | miRNA-down_mRNA-up |
| hsa-miR-129-5p | AJUBA | miRNA-down_mRNA-up |
| hsa-miR-129-5p | FOXG1 | miRNA-down_mRNA-up |
| hsa-miR-129-5p | RBFOX1 | miRNA-down_mRNA-up |
| hsa-miR-129-5p | IGF2BP1 | miRNA-down_mRNA-up |
| hsa-miR-129-5p | SOX9 | miRNA-down_mRNA-up |
| hsa-miR-129-5p | TYMS | miRNA-down_mRNA-up |
| hsa-miR-129-5p | APCDD1 | miRNA-down_mRNA-up |
| hsa-miR-129-5p | ONECUT2 | miRNA-down_mRNA-up |
| hsa-miR-129-5p | ICAM1 | miRNA-down_mRNA-up |
| hsa-miR-129-5p | PPM1N | miRNA-down_mRNA-up |
| hsa-miR-129-5p | BCL2L12 | miRNA-down_mRNA-up |
| hsa-miR-129-5p | STAT1 | miRNA-down_mRNA-up |
| hsa-miR-129-5p | SOX11 | miRNA-down_mRNA-up |
| hsa-miR-129-5p | SCN2A | miRNA-down_mRNA-up |
| hsa-miR-129-5p | PMEPA1 | miRNA-down_mRNA-up |
| hsa-miR-129-5p | NOP56 | miRNA-down_mRNA-up |
| hsa-miR-129-5p | TGIF2 | miRNA-down_mRNA-up |
| hsa-miR-129-5p | DONSON | miRNA-down_mRNA-up |
| hsa-miR-129-5p | RUNX1 | miRNA-down_mRNA-up |
| hsa-miR-129-5p | WNT5A | miRNA-down_mRNA-up |
| hsa-miR-129-5p | EIF5A2 | miRNA-down_mRNA-up |
| hsa-miR-129-5p | CLDN1 | miRNA-down_mRNA-up |
| hsa-miR-129-5p | TRIM71 | miRNA-down_mRNA-up |
| hsa-miR-129-5p | COL8A1 | miRNA-down_mRNA-up |
| hsa-miR-129-5p | SOX2 | miRNA-down_mRNA-up |
| hsa-miR-129-5p | SLC7A11 | miRNA-down_mRNA-up |
| hsa-miR-129-5p | ALB | miRNA-down_mRNA-up |
| hsa-miR-129-5p | ACSL6 | miRNA-down_mRNA-up |
| hsa-miR-129-5p | PITX1 | miRNA-down_mRNA-up |
| hsa-miR-129-5p | SKP2 | miRNA-down_mRNA-up |
| hsa-miR-129-5p | PCDHGA1 | miRNA-down_mRNA-up |
| hsa-miR-129-5p | PCDHGC5 | miRNA-down_mRNA-up |
| hsa-miR-129-5p | PCDHGA8 | miRNA-down_mRNA-up |
| hsa-miR-129-5p | E2F3 | miRNA-down_mRNA-up |
| hsa-miR-129-5p | SOX4 | miRNA-down_mRNA-up |
| hsa-miR-129-5p | POU3F2 | miRNA-down_mRNA-up |
| hsa-miR-129-5p | OSBPL3 | miRNA-down_mRNA-up |
| hsa-miR-129-5p | INHBA | miRNA-down_mRNA-up |
| hsa-miR-129-5p | NRCAM | miRNA-down_mRNA-up |
| hsa-miR-129-5p | CHD7 | miRNA-down_mRNA-up |
| hsa-miR-129-5p | CLVS1 | miRNA-down_mRNA-up |
| hsa-miR-129-5p | PI15 | miRNA-down_mRNA-up |
| hsa-miR-129-5p | LAPTM4B | miRNA-down_mRNA-up |
| hsa-miR-129-5p | FZD6 | miRNA-down_mRNA-up |
| hsa-miR-129-5p | COL27A1 | miRNA-down_mRNA-up |
| hsa-miR-129-5p | ZNF280C | miRNA-down_mRNA-up |
| hsa-miR-129-5p | CLCN4 | miRNA-down_mRNA-up |
| hsa-miR-129-5p | DKC1 | miRNA-down_mRNA-up |
| hsa-miR-125b-5p | E2F2 | miRNA-down_mRNA-up |
| hsa-miR-125b-5p | TMEM63A | miRNA-down_mRNA-up |
| hsa-miR-125b-5p | PLEKHN1 | miRNA-down_mRNA-up |
| hsa-miR-125b-5p | AGTRAP | miRNA-down_mRNA-up |
| hsa-miR-125b-5p | FOXD2 | miRNA-down_mRNA-up |
| hsa-miR-125b-5p | CACNA1E | miRNA-down_mRNA-up |
| hsa-miR-125b-5p | CNNM1 | miRNA-down_mRNA-up |
| hsa-miR-125b-5p | SCD | miRNA-down_mRNA-up |
| hsa-miR-125b-5p | FADS2 | miRNA-down_mRNA-up |
| hsa-miR-125b-5p | BCAT1 | miRNA-down_mRNA-up |
| hsa-miR-125b-5p | SLC7A1 | miRNA-down_mRNA-up |
| hsa-miR-125b-5p | COL4A1 | miRNA-down_mRNA-up |
| hsa-miR-125b-5p | WARS1 | miRNA-down_mRNA-up |
| hsa-miR-125b-5p | OTUB2 | miRNA-down_mRNA-up |
| hsa-miR-125b-5p | FRMD5 | miRNA-down_mRNA-up |
| hsa-miR-125b-5p | ALPK3 | miRNA-down_mRNA-up |
| hsa-miR-125b-5p | ASPHD1 | miRNA-down_mRNA-up |
| hsa-miR-125b-5p | GINS3 | miRNA-down_mRNA-up |
| hsa-miR-125b-5p | CBFB | miRNA-down_mRNA-up |
| hsa-miR-125b-5p | SLC7A6 | miRNA-down_mRNA-up |
| hsa-miR-125b-5p | TBC1D16 | miRNA-down_mRNA-up |
| hsa-miR-125b-5p | C18orf54 | miRNA-down_mRNA-up |
| hsa-miR-125b-5p | SLC1A5 | miRNA-down_mRNA-up |
| hsa-miR-125b-5p | POLR1G | miRNA-down_mRNA-up |
| hsa-miR-125b-5p | BCL2L12 | miRNA-down_mRNA-up |
| hsa-miR-125b-5p | HOXD1 | miRNA-down_mRNA-up |
| hsa-miR-125b-5p | SCLY | miRNA-down_mRNA-up |
| hsa-miR-125b-5p | SNRPB | miRNA-down_mRNA-up |
| hsa-miR-125b-5p | XKR7 | miRNA-down_mRNA-up |
| hsa-miR-125b-5p | PHACTR3 | miRNA-down_mRNA-up |
| hsa-miR-125b-5p | LIF | miRNA-down_mRNA-up |
| hsa-miR-125b-5p | FOXRED2 | miRNA-down_mRNA-up |
| hsa-miR-125b-5p | MTFP1 | miRNA-down_mRNA-up |
| hsa-miR-125b-5p | NUP210 | miRNA-down_mRNA-up |
| hsa-miR-125b-5p | TRIM71 | miRNA-down_mRNA-up |
| hsa-miR-125b-5p | PLXNA1 | miRNA-down_mRNA-up |
| hsa-miR-125b-5p | FOXQ1 | miRNA-down_mRNA-up |
| hsa-miR-125b-5p | E2F3 | miRNA-down_mRNA-up |
| hsa-miR-125b-5p | POU3F2 | miRNA-down_mRNA-up |
| hsa-miR-125b-5p | PODXL | miRNA-down_mRNA-up |
| hsa-miR-125b-5p | LIMK1 | miRNA-down_mRNA-up |
| hsa-miR-125b-5p | TNFRSF10B | miRNA-down_mRNA-up |
| hsa-miR-125b-5p | STC1 | miRNA-down_mRNA-up |
| hsa-miR-125b-5p | EIF4EBP1 | miRNA-down_mRNA-up |
| hsa-miR-125b-5p | ARHGEF39 | miRNA-down_mRNA-up |
| hsa-miR-125b-5p | NR6A1 | miRNA-down_mRNA-up |
| hsa-miR-125b-5p | IRAK1 | miRNA-down_mRNA-up |
| hsa-miR-125b-5p | SUV39H1 | miRNA-down_mRNA-up |
| hsa-miR-125b-5p | HMGB3 | miRNA-down_mRNA-up |
| hsa-miR-125a-5p | E2F2 | miRNA-down_mRNA-up |
| hsa-miR-125a-5p | TMEM63A | miRNA-down_mRNA-up |
| hsa-miR-125a-5p | PLEKHN1 | miRNA-down_mRNA-up |
| hsa-miR-125a-5p | AGTRAP | miRNA-down_mRNA-up |
| hsa-miR-125a-5p | FOXD2 | miRNA-down_mRNA-up |
| hsa-miR-125a-5p | CACNA1E | miRNA-down_mRNA-up |
| hsa-miR-125a-5p | CNNM1 | miRNA-down_mRNA-up |
| hsa-miR-125a-5p | SCD | miRNA-down_mRNA-up |
| hsa-miR-125a-5p | FADS2 | miRNA-down_mRNA-up |
| hsa-miR-125a-5p | BCAT1 | miRNA-down_mRNA-up |
| hsa-miR-125a-5p | OAS3 | miRNA-down_mRNA-up |
| hsa-miR-125a-5p | ARL6IP4 | miRNA-down_mRNA-up |
| hsa-miR-125a-5p | SLC7A1 | miRNA-down_mRNA-up |
| hsa-miR-125a-5p | COL4A1 | miRNA-down_mRNA-up |
| hsa-miR-125a-5p | WARS1 | miRNA-down_mRNA-up |
| hsa-miR-125a-5p | OTUB2 | miRNA-down_mRNA-up |
| hsa-miR-125a-5p | FRMD5 | miRNA-down_mRNA-up |
| hsa-miR-125a-5p | ALPK3 | miRNA-down_mRNA-up |
| hsa-miR-125a-5p | GINS3 | miRNA-down_mRNA-up |
| hsa-miR-125a-5p | CBFB | miRNA-down_mRNA-up |
| hsa-miR-125a-5p | SLC7A6 | miRNA-down_mRNA-up |
| hsa-miR-125a-5p | NAT9 | miRNA-down_mRNA-up |
| hsa-miR-125a-5p | TBC1D16 | miRNA-down_mRNA-up |
| hsa-miR-125a-5p | C18orf54 | miRNA-down_mRNA-up |
| hsa-miR-125a-5p | SLC1A5 | miRNA-down_mRNA-up |
| hsa-miR-125a-5p | POLR1G | miRNA-down_mRNA-up |
| hsa-miR-125a-5p | BCL2L12 | miRNA-down_mRNA-up |
| hsa-miR-125a-5p | HOXD1 | miRNA-down_mRNA-up |
| hsa-miR-125a-5p | SCLY | miRNA-down_mRNA-up |
| hsa-miR-125a-5p | SNRPB | miRNA-down_mRNA-up |
| hsa-miR-125a-5p | PHACTR3 | miRNA-down_mRNA-up |
| hsa-miR-125a-5p | LIF | miRNA-down_mRNA-up |
| hsa-miR-125a-5p | FOXRED2 | miRNA-down_mRNA-up |
| hsa-miR-125a-5p | MTFP1 | miRNA-down_mRNA-up |
| hsa-miR-125a-5p | NUP210 | miRNA-down_mRNA-up |
| hsa-miR-125a-5p | TRIM71 | miRNA-down_mRNA-up |
| hsa-miR-125a-5p | PLXNA1 | miRNA-down_mRNA-up |
| hsa-miR-125a-5p | FOXQ1 | miRNA-down_mRNA-up |
| hsa-miR-125a-5p | E2F3 | miRNA-down_mRNA-up |
| hsa-miR-125a-5p | POU3F2 | miRNA-down_mRNA-up |
| hsa-miR-125a-5p | PODXL | miRNA-down_mRNA-up |
| hsa-miR-125a-5p | LIMK1 | miRNA-down_mRNA-up |
| hsa-miR-125a-5p | TNFRSF10B | miRNA-down_mRNA-up |
| hsa-miR-125a-5p | STC1 | miRNA-down_mRNA-up |
| hsa-miR-125a-5p | EIF4EBP1 | miRNA-down_mRNA-up |
| hsa-miR-125a-5p | ARHGEF39 | miRNA-down_mRNA-up |
| hsa-miR-125a-5p | NR6A1 | miRNA-down_mRNA-up |
| hsa-miR-125a-5p | QSOX2 | miRNA-down_mRNA-up |
| hsa-miR-125a-5p | IRAK1 | miRNA-down_mRNA-up |
| hsa-miR-125a-5p | SUV39H1 | miRNA-down_mRNA-up |
| hsa-miR-125a-5p | HMGB3 | miRNA-down_mRNA-up |
| TPTEP1 | hsa-miR-518a-3p | lncRNA-down_miRNA-up |
| TTTY14 | hsa-miR-518a-3p | lncRNA-down_miRNA-up |
| ZNF781 | hsa-miR-518a-3p | lncRNA-down_miRNA-up |
| SNHG14 | hsa-miR-518a-3p | lncRNA-down_miRNA-up |
| LINC00365 | hsa-miR-518a-3p | lncRNA-down_miRNA-up |
| CT62 | hsa-miR-518a-3p | lncRNA-down_miRNA-up |
| PCA3 | hsa-miR-518a-3p | lncRNA-down_miRNA-up |
| DNMBP-AS1 | hsa-miR-518a-3p | lncRNA-down_miRNA-up |
| JAZF1-AS1 | hsa-miR-518a-3p | lncRNA-down_miRNA-up |
| LINC00330 | hsa-miR-518a-3p | lncRNA-down_miRNA-up |
| LINC00402 | hsa-miR-518a-3p | lncRNA-down_miRNA-up |
| ABCC13 | hsa-miR-518a-3p | lncRNA-down_miRNA-up |
| ARHGEF26-AS1 | hsa-miR-518a-3p | lncRNA-down_miRNA-up |
| ALDH1L1-AS2 | hsa-miR-518a-3p | lncRNA-down_miRNA-up |
| TPTEP1 | hsa-miR-206 | lncRNA-down_miRNA-up |
| PART1 | hsa-miR-206 | lncRNA-down_miRNA-up |
| WDFY3-AS2 | hsa-miR-206 | lncRNA-down_miRNA-up |
| MIR22HG | hsa-miR-206 | lncRNA-down_miRNA-up |
| ANKRD20A11P | hsa-miR-206 | lncRNA-down_miRNA-up |
| SNHG14 | hsa-miR-206 | lncRNA-down_miRNA-up |
| WARS2-IT1 | hsa-miR-206 | lncRNA-down_miRNA-up |
| TMEM72-AS1 | hsa-miR-206 | lncRNA-down_miRNA-up |
| CT62 | hsa-miR-206 | lncRNA-down_miRNA-up |
| PCA3 | hsa-miR-206 | lncRNA-down_miRNA-up |
| DNMBP-AS1 | hsa-miR-206 | lncRNA-down_miRNA-up |
| ANKRD36BP2 | hsa-miR-206 | lncRNA-down_miRNA-up |
| DIRC3 | hsa-miR-206 | lncRNA-down_miRNA-up |
| CYP1B1-AS1 | hsa-miR-206 | lncRNA-down_miRNA-up |
| ZFY-AS1 | hsa-miR-206 | lncRNA-down_miRNA-up |
| LINC00163 | hsa-miR-206 | lncRNA-down_miRNA-up |
| LINC00330 | hsa-miR-206 | lncRNA-down_miRNA-up |
| ABCC13 | hsa-miR-206 | lncRNA-down_miRNA-up |
| LIFR-AS1 | hsa-miR-206 | lncRNA-down_miRNA-up |
| FRMD6-AS2 | hsa-miR-206 | lncRNA-down_miRNA-up |
| LINC00261 | hsa-miR-206 | lncRNA-down_miRNA-up |
| TPTEP1 | hsa-miR-216b-5p | lncRNA-down_miRNA-up |
| FOLH1B | hsa-miR-216b-5p | lncRNA-down_miRNA-up |
| SNHG14 | hsa-miR-216b-5p | lncRNA-down_miRNA-up |
| WARS2-IT1 | hsa-miR-216b-5p | lncRNA-down_miRNA-up |
| LINC00365 | hsa-miR-216b-5p | lncRNA-down_miRNA-up |
| TMEM72-AS1 | hsa-miR-216b-5p | lncRNA-down_miRNA-up |
| FAM138B | hsa-miR-216b-5p | lncRNA-down_miRNA-up |
| LINC00472 | hsa-miR-216b-5p | lncRNA-down_miRNA-up |
| JAZF1-AS1 | hsa-miR-216b-5p | lncRNA-down_miRNA-up |
| MAGI2-AS3 | hsa-miR-216b-5p | lncRNA-down_miRNA-up |
| LINC00402 | hsa-miR-216b-5p | lncRNA-down_miRNA-up |
| ABCC13 | hsa-miR-216b-5p | lncRNA-down_miRNA-up |
| LIFR-AS1 | hsa-miR-216b-5p | lncRNA-down_miRNA-up |
| LINC00461 | hsa-miR-216b-5p | lncRNA-down_miRNA-up |
| LINC00507 | hsa-miR-216b-5p | lncRNA-down_miRNA-up |
| PWRN1 | hsa-miR-216b-5p | lncRNA-down_miRNA-up |
| LINC00261 | hsa-miR-216b-5p | lncRNA-down_miRNA-up |
| TPTEP1 | hsa-miR-338-3p | lncRNA-down_miRNA-up |
| TTTY14 | hsa-miR-338-3p | lncRNA-down_miRNA-up |
| WDFY3-AS2 | hsa-miR-338-3p | lncRNA-down_miRNA-up |
| ZNF300P1 | hsa-miR-338-3p | lncRNA-down_miRNA-up |
| KRT16P3 | hsa-miR-338-3p | lncRNA-down_miRNA-up |
| ANKRD20A11P | hsa-miR-338-3p | lncRNA-down_miRNA-up |
| SNHG14 | hsa-miR-338-3p | lncRNA-down_miRNA-up |
| LINC00365 | hsa-miR-338-3p | lncRNA-down_miRNA-up |
| TMEM72-AS1 | hsa-miR-338-3p | lncRNA-down_miRNA-up |
| CYP4F29P | hsa-miR-338-3p | lncRNA-down_miRNA-up |
| ANKRD36BP2 | hsa-miR-338-3p | lncRNA-down_miRNA-up |
| DIRC3 | hsa-miR-338-3p | lncRNA-down_miRNA-up |
| CYP1B1-AS1 | hsa-miR-338-3p | lncRNA-down_miRNA-up |
| LINC00402 | hsa-miR-338-3p | lncRNA-down_miRNA-up |
| ST3GAL6-AS1 | hsa-miR-338-3p | lncRNA-down_miRNA-up |
| ABCC13 | hsa-miR-338-3p | lncRNA-down_miRNA-up |
| LINC00461 | hsa-miR-338-3p | lncRNA-down_miRNA-up |
| FRMD6-AS2 | hsa-miR-338-3p | lncRNA-down_miRNA-up |
| PWRN1 | hsa-miR-338-3p | lncRNA-down_miRNA-up |
| LINC00261 | hsa-miR-338-3p | lncRNA-down_miRNA-up |
| NAPSB | hsa-miR-184 | lncRNA-down_miRNA-up |
| TTTY14 | hsa-miR-184 | lncRNA-down_miRNA-up |
| TMEM72-AS1 | hsa-miR-184 | lncRNA-down_miRNA-up |
| LINC00472 | hsa-miR-184 | lncRNA-down_miRNA-up |
| FRMD6-AS2 | hsa-miR-184 | lncRNA-down_miRNA-up |
| PWRN1 | hsa-miR-184 | lncRNA-down_miRNA-up |
| hsa-miR-518a-3p | CSRP1 | miRNA-up_mRNA-down |
| hsa-miR-518a-3p | FRMD6 | miRNA-up_mRNA-down |
| hsa-miR-518a-3p | EGR1 | miRNA-up_mRNA-down |
| hsa-miR-206 | SLC35D1 | miRNA-up_mRNA-down |
| hsa-miR-206 | KCNJ10 | miRNA-up_mRNA-down |
| hsa-miR-206 | MPZ | miRNA-up_mRNA-down |
| hsa-miR-206 | SH2D1B | miRNA-up_mRNA-down |
| hsa-miR-206 | NIBAN1 | miRNA-up_mRNA-down |
| hsa-miR-206 | AK5 | miRNA-up_mRNA-down |
| hsa-miR-206 | PRKACB | miRNA-up_mRNA-down |
| hsa-miR-206 | PBX1 | miRNA-up_mRNA-down |
| hsa-miR-206 | PPP1R12B | miRNA-up_mRNA-down |
| hsa-miR-206 | ATP2B4 | miRNA-up_mRNA-down |
| hsa-miR-206 | ACTA2 | miRNA-up_mRNA-down |
| hsa-miR-206 | ZNF365 | miRNA-up_mRNA-down |
| hsa-miR-206 | PDCD4 | miRNA-up_mRNA-down |
| hsa-miR-206 | CDON | miRNA-up_mRNA-down |
| hsa-miR-206 | NCAM1 | miRNA-up_mRNA-down |
| hsa-miR-206 | EMP1 | miRNA-up_mRNA-down |
| hsa-miR-206 | MSRB3 | miRNA-up_mRNA-down |
| hsa-miR-206 | AMER2 | miRNA-up_mRNA-down |
| hsa-miR-206 | SLC46A3 | miRNA-up_mRNA-down |
| hsa-miR-206 | MAB21L1 | miRNA-up_mRNA-down |
| hsa-miR-206 | NBEA | miRNA-up_mRNA-down |
| hsa-miR-206 | SLITRK5 | miRNA-up_mRNA-down |
| hsa-miR-206 | FOXN3 | miRNA-up_mRNA-down |
| hsa-miR-206 | THBS1 | miRNA-up_mRNA-down |
| hsa-miR-206 | MAP1A | miRNA-up_mRNA-down |
| hsa-miR-206 | MAPK3 | miRNA-up_mRNA-down |
| hsa-miR-206 | PHLPP2 | miRNA-up_mRNA-down |
| hsa-miR-206 | MAF | miRNA-up_mRNA-down |
| hsa-miR-206 | VAT1L | miRNA-up_mRNA-down |
| hsa-miR-206 | VAMP2 | miRNA-up_mRNA-down |
| hsa-miR-206 | PMP22 | miRNA-up_mRNA-down |
| hsa-miR-206 | GJC1 | miRNA-up_mRNA-down |
| hsa-miR-206 | MYOCD | miRNA-up_mRNA-down |
| hsa-miR-206 | ITGB3 | miRNA-up_mRNA-down |
| hsa-miR-206 | RNF165 | miRNA-up_mRNA-down |
| hsa-miR-206 | DMPK | miRNA-up_mRNA-down |
| hsa-miR-206 | AFF3 | miRNA-up_mRNA-down |
| hsa-miR-206 | CAVIN2 | miRNA-up_mRNA-down |
| hsa-miR-206 | MXD1 | miRNA-up_mRNA-down |
| hsa-miR-206 | RCAN1 | miRNA-up_mRNA-down |
| hsa-miR-206 | NCAM2 | miRNA-up_mRNA-down |
| hsa-miR-206 | TIMP3 | miRNA-up_mRNA-down |
| hsa-miR-206 | MYLK | miRNA-up_mRNA-down |
| hsa-miR-206 | C3orf70 | miRNA-up_mRNA-down |
| hsa-miR-206 | LIPH | miRNA-up_mRNA-down |
| hsa-miR-206 | LPP | miRNA-up_mRNA-down |
| hsa-miR-206 | SMIM14 | miRNA-up_mRNA-down |
| hsa-miR-206 | TBC1D9 | miRNA-up_mRNA-down |
| hsa-miR-206 | BMPR1B | miRNA-up_mRNA-down |
| hsa-miR-206 | FNIP2 | miRNA-up_mRNA-down |
| hsa-miR-206 | IL6ST | miRNA-up_mRNA-down |
| hsa-miR-206 | PPP2R2B | miRNA-up_mRNA-down |
| hsa-miR-206 | MAN1A1 | miRNA-up_mRNA-down |
| hsa-miR-206 | SLC2A12 | miRNA-up_mRNA-down |
| hsa-miR-206 | SGK1 | miRNA-up_mRNA-down |
| hsa-miR-206 | ID4 | miRNA-up_mRNA-down |
| hsa-miR-206 | PIM1 | miRNA-up_mRNA-down |
| hsa-miR-206 | MAGI2 | miRNA-up_mRNA-down |
| hsa-miR-206 | DLC1 | miRNA-up_mRNA-down |
| hsa-miR-206 | SFRP1 | miRNA-up_mRNA-down |
| hsa-miR-206 | TOX | miRNA-up_mRNA-down |
| hsa-miR-206 | SLC7A2 | miRNA-up_mRNA-down |
| hsa-miR-206 | SLC39A14 | miRNA-up_mRNA-down |
| hsa-miR-206 | KLF4 | miRNA-up_mRNA-down |
| hsa-miR-206 | ACER2 | miRNA-up_mRNA-down |
| hsa-miR-206 | PTGS1 | miRNA-up_mRNA-down |
| hsa-miR-206 | KLHL15 | miRNA-up_mRNA-down |
| hsa-miR-206 | NEXMIF | miRNA-up_mRNA-down |
| hsa-miR-206 | SH3BGRL | miRNA-up_mRNA-down |
| hsa-miR-206 | BEX4 | miRNA-up_mRNA-down |
| hsa-miR-216b-5p | JUN | miRNA-up_mRNA-down |
| hsa-miR-216b-5p | TTLL7 | miRNA-up_mRNA-down |
| hsa-miR-216b-5p | NIBAN1 | miRNA-up_mRNA-down |
| hsa-miR-216b-5p | PRKAA2 | miRNA-up_mRNA-down |
| hsa-miR-216b-5p | CPEB3 | miRNA-up_mRNA-down |
| hsa-miR-216b-5p | ABLIM1 | miRNA-up_mRNA-down |
| hsa-miR-216b-5p | ATRNL1 | miRNA-up_mRNA-down |
| hsa-miR-216b-5p | PGR | miRNA-up_mRNA-down |
| hsa-miR-216b-5p | CRY2 | miRNA-up_mRNA-down |
| hsa-miR-216b-5p | DIXDC1 | miRNA-up_mRNA-down |
| hsa-miR-216b-5p | SOX5 | miRNA-up_mRNA-down |
| hsa-miR-216b-5p | GABARAPL1 | miRNA-up_mRNA-down |
| hsa-miR-216b-5p | EMP1 | miRNA-up_mRNA-down |
| hsa-miR-216b-5p | PDE3A | miRNA-up_mRNA-down |
| hsa-miR-216b-5p | FOXN3 | miRNA-up_mRNA-down |
| hsa-miR-216b-5p | PELI2 | miRNA-up_mRNA-down |
| hsa-miR-216b-5p | SOCS3 | miRNA-up_mRNA-down |
| hsa-miR-216b-5p | HLF | miRNA-up_mRNA-down |
| hsa-miR-216b-5p | EPB41L3 | miRNA-up_mRNA-down |
| hsa-miR-216b-5p | AQP4 | miRNA-up_mRNA-down |
| hsa-miR-216b-5p | ATP8B1 | miRNA-up_mRNA-down |
| hsa-miR-216b-5p | CLIP3 | miRNA-up_mRNA-down |
| hsa-miR-216b-5p | SEZ6L | miRNA-up_mRNA-down |
| hsa-miR-216b-5p | CD200R1 | miRNA-up_mRNA-down |
| hsa-miR-216b-5p | GPD1L | miRNA-up_mRNA-down |
| hsa-miR-216b-5p | CADM2 | miRNA-up_mRNA-down |
| hsa-miR-216b-5p | LPP | miRNA-up_mRNA-down |
| hsa-miR-216b-5p | EPHA5 | miRNA-up_mRNA-down |
| hsa-miR-216b-5p | CDKL2 | miRNA-up_mRNA-down |
| hsa-miR-216b-5p | FAM13A | miRNA-up_mRNA-down |
| hsa-miR-216b-5p | NPR3 | miRNA-up_mRNA-down |
| hsa-miR-216b-5p | ADRB2 | miRNA-up_mRNA-down |
| hsa-miR-216b-5p | GABRA1 | miRNA-up_mRNA-down |
| hsa-miR-216b-5p | SGK1 | miRNA-up_mRNA-down |
| hsa-miR-216b-5p | GCNT2 | miRNA-up_mRNA-down |
| hsa-miR-216b-5p | SLC35F1 | miRNA-up_mRNA-down |
| hsa-miR-216b-5p | FGFR1 | miRNA-up_mRNA-down |
| hsa-miR-216b-5p | RUNX1T1 | miRNA-up_mRNA-down |
| hsa-miR-216b-5p | KLF9 | miRNA-up_mRNA-down |
| hsa-miR-216b-5p | KLHL15 | miRNA-up_mRNA-down |
| hsa-miR-338-3p | EDN2 | miRNA-up_mRNA-down |
| hsa-miR-338-3p | SLC35D1 | miRNA-up_mRNA-down |
| hsa-miR-338-3p | KCNJ10 | miRNA-up_mRNA-down |
| hsa-miR-338-3p | LEPR | miRNA-up_mRNA-down |
| hsa-miR-338-3p | LRRC7 | miRNA-up_mRNA-down |
| hsa-miR-338-3p | ACADM | miRNA-up_mRNA-down |
| hsa-miR-338-3p | PBX1 | miRNA-up_mRNA-down |
| hsa-miR-338-3p | DIP2C | miRNA-up_mRNA-down |
| hsa-miR-338-3p | SKIDA1 | miRNA-up_mRNA-down |
| hsa-miR-338-3p | MPP7 | miRNA-up_mRNA-down |
| hsa-miR-338-3p | VSIR | miRNA-up_mRNA-down |
| hsa-miR-338-3p | MYPN | miRNA-up_mRNA-down |
| hsa-miR-338-3p | LDB3 | miRNA-up_mRNA-down |
| hsa-miR-338-3p | PGR | miRNA-up_mRNA-down |
| hsa-miR-338-3p | SCN2B | miRNA-up_mRNA-down |
| hsa-miR-338-3p | TM7SF2 | miRNA-up_mRNA-down |
| hsa-miR-338-3p | JAM3 | miRNA-up_mRNA-down |
| hsa-miR-338-3p | EMP1 | miRNA-up_mRNA-down |
| hsa-miR-338-3p | NDRG2 | miRNA-up_mRNA-down |
| hsa-miR-338-3p | PTPN21 | miRNA-up_mRNA-down |
| hsa-miR-338-3p | SSTR1 | miRNA-up_mRNA-down |
| hsa-miR-338-3p | NRXN3 | miRNA-up_mRNA-down |
| hsa-miR-338-3p | SNRPN | miRNA-up_mRNA-down |
| hsa-miR-338-3p | THBS1 | miRNA-up_mRNA-down |
| hsa-miR-338-3p | MAP1A | miRNA-up_mRNA-down |
| hsa-miR-338-3p | TPM1 | miRNA-up_mRNA-down |
| hsa-miR-338-3p | MAF | miRNA-up_mRNA-down |
| hsa-miR-338-3p | PRKCB | miRNA-up_mRNA-down |
| hsa-miR-338-3p | CRISPLD2 | miRNA-up_mRNA-down |
| hsa-miR-338-3p | VAMP2 | miRNA-up_mRNA-down |
| hsa-miR-338-3p | MXRA7 | miRNA-up_mRNA-down |
| hsa-miR-338-3p | NTN1 | miRNA-up_mRNA-down |
| hsa-miR-338-3p | UNC45B | miRNA-up_mRNA-down |
| hsa-miR-338-3p | RNF152 | miRNA-up_mRNA-down |
| hsa-miR-338-3p | GNAL | miRNA-up_mRNA-down |
| hsa-miR-338-3p | DTNA | miRNA-up_mRNA-down |
| hsa-miR-338-3p | NEDD4L | miRNA-up_mRNA-down |
| hsa-miR-338-3p | SLC25A23 | miRNA-up_mRNA-down |
| hsa-miR-338-3p | JUNB | miRNA-up_mRNA-down |
| hsa-miR-338-3p | MYT1L | miRNA-up_mRNA-down |
| hsa-miR-338-3p | PAIP2B | miRNA-up_mRNA-down |
| hsa-miR-338-3p | FRZB | miRNA-up_mRNA-down |
| hsa-miR-338-3p | SPHKAP | miRNA-up_mRNA-down |
| hsa-miR-338-3p | EHD3 | miRNA-up_mRNA-down |
| hsa-miR-338-3p | DES | miRNA-up_mRNA-down |
| hsa-miR-338-3p | BTBD3 | miRNA-up_mRNA-down |
| hsa-miR-338-3p | CHL1 | miRNA-up_mRNA-down |
| hsa-miR-338-3p | ARPP21 | miRNA-up_mRNA-down |
| hsa-miR-338-3p | BEND4 | miRNA-up_mRNA-down |
| hsa-miR-338-3p | PCDH7 | miRNA-up_mRNA-down |
| hsa-miR-338-3p | SLC4A4 | miRNA-up_mRNA-down |
| hsa-miR-338-3p | GRIA2 | miRNA-up_mRNA-down |
| hsa-miR-338-3p | PALLD | miRNA-up_mRNA-down |
| hsa-miR-338-3p | HCN1 | miRNA-up_mRNA-down |
| hsa-miR-338-3p | ADRB2 | miRNA-up_mRNA-down |
| hsa-miR-338-3p | CNR1 | miRNA-up_mRNA-down |
| hsa-miR-338-3p | BVES | miRNA-up_mRNA-down |
| hsa-miR-338-3p | PIM1 | miRNA-up_mRNA-down |
| hsa-miR-338-3p | ARG1 | miRNA-up_mRNA-down |
| hsa-miR-338-3p | AKAP12 | miRNA-up_mRNA-down |
| hsa-miR-338-3p | PDE1C | miRNA-up_mRNA-down |
| hsa-miR-338-3p | CAV1 | miRNA-up_mRNA-down |
| hsa-miR-338-3p | AHCYL2 | miRNA-up_mRNA-down |
| hsa-miR-338-3p | FGFR1 | miRNA-up_mRNA-down |
| hsa-miR-338-3p | NCALD | miRNA-up_mRNA-down |
| hsa-miR-338-3p | SLC7A2 | miRNA-up_mRNA-down |
| hsa-miR-338-3p | SLC39A14 | miRNA-up_mRNA-down |
| hsa-miR-338-3p | ENHO | miRNA-up_mRNA-down |
| hsa-miR-184 | PLPP3 | miRNA-up_mRNA-down |
| hsa-miR-184 | ZDHHC22 | miRNA-up_mRNA-down |
| hsa-miR-184 | RGMA | miRNA-up_mRNA-down |
| hsa-miR-184 | EPB41L3 | miRNA-up_mRNA-down |
| hsa-miR-184 | ACO2 | miRNA-up_mRNA-down |
| hsa-miR-184 | CAMK2A | miRNA-up_mRNA-down |
| hsa-miR-184 | FGFR1 | miRNA-up_mRNA-down |

**Table S4**. The dysregulation of SGK1 related network nodes

| RNA types | RNA names | cancer types | nodes dysregulation types |
| --- | --- | --- | --- |
| mRNA | SGK1 | STAD | mRNA-down |
| mRNA | SGK1 | LIHC | mRNA-down |
| mRNA | SGK1 | COAD | mRNA-down |
| miRNA | hsa-miR-216b-5p | STAD | miRNA-up |
| miRNA | hsa-miR-216b-5p | LIHC | miRNA-up |
| miRNA | hsa-miR-206 | LIHC | miRNA-up |
| miRNA | hsa-miR-206 | COAD | miRNA-up |
| lncRNA | TPTEP1 | STAD | lncRNA-down |
| lncRNA | FOLH1B | STAD | lncRNA-down |
| lncRNA | SNHG14 | STAD | lncRNA-down |
| lncRNA | WARS2-IT1 | STAD | lncRNA-down |
| lncRNA | LINC00365 | STAD | lncRNA-down |
| lncRNA | FAM138B | STAD | lncRNA-down |
| lncRNA | JAZF1-AS1 | STAD | lncRNA-down |
| lncRNA | ABCC13 | STAD | lncRNA-down |
| lncRNA | LINC00461 | STAD | lncRNA-down |
| lncRNA | FOLH1B | LIHC | lncRNA-down |
| lncRNA | WARS2-IT1 | LIHC | lncRNA-down |
| lncRNA | LINC00365 | LIHC | lncRNA-down |
| lncRNA | MAGI2-AS3 | LIHC | lncRNA-down |
| lncRNA | LINC00402 | LIHC | lncRNA-down |
| lncRNA | PWRN1 | LIHC | lncRNA-down |
| lncRNA | LINC00261 | LIHC | lncRNA-down |
| lncRNA | WDFY3-AS2 | LIHC | lncRNA-down |
| lncRNA | PCA3 | LIHC | lncRNA-down |
| lncRNA | TPTEP1 | COAD | lncRNA-down |
| lncRNA | MIR22HG | COAD | lncRNA-down |
| lncRNA | ANKRD20A11P | COAD | lncRNA-down |
| lncRNA | TMEM72-AS1 | COAD | lncRNA-down |
| lncRNA | CT62 | COAD | lncRNA-down |
| lncRNA | DNMBP-AS1 | COAD | lncRNA-down |
| lncRNA | ANKRD36BP2 | COAD | lncRNA-down |
| lncRNA | DIRC3 | COAD | lncRNA-down |
| lncRNA | CYP1B1-AS1 | COAD | lncRNA-down |
| lncRNA | ZFY-AS1 | COAD | lncRNA-down |
| lncRNA | LINC00163 | COAD | lncRNA-down |
| lncRNA | LINC00330 | COAD | lncRNA-down |
| lncRNA | ABCC13 | COAD | lncRNA-down |
| lncRNA | LIFR-AS1 | COAD | lncRNA-down |
| lncRNA | FRMD6-AS2 | COAD | lncRNA-down |

**Table S5**. Univariate Cox regression analysis of ceRNA network nodes.

| cancer types | node types | RNA types | id | HR | pvalue |
| --- | --- | --- | --- | --- | --- |
| ESCA | semi-shared | mRNA | SNX10 | 1.239803661 | 4.34E-02 |
| ESCA | semi-shared | mRNA | RGS16 | 1.319508972 | 4.67E-03 |
| ESCA | semi-shared | mRNA | HMGB3 | 1.397802915 | 8.92E-03 |
| ESCA | semi-shared | lncRNA | GK-IT1 | 1.303160025 | 3.73E-03 |
| ESCA | semi-shared | lncRNA | ALMS1-IT1 | 1.305105584 | 2.59E-02 |
| ESCA | semi-shared | lncRNA | DLEU2 | 1.425317914 | 2.54E-02 |
| STAD | shared | mRNA | CCNF | 0.813598938 | 1.43E-02 |
| STAD | shared | mRNA | CEP55 | 0.850620706 | 3.48E-02 |
| STAD | shared | mRNA | LRP8 | 0.865194034 | 3.10E-02 |
| STAD | shared | mRNA | SAPCD2 | 0.867102381 | 1.28E-02 |
| STAD | shared | mRNA | CDCA5 | 0.869267589 | 4.62E-02 |
| STAD | shared | mRNA | LIN28B | 1.05502221 | 4.12E-02 |
| STAD | shared | mRNA | CHRM2 | 1.060110855 | 1.36E-02 |
| STAD | shared | mRNA | HMGA2 | 1.075850011 | 4.73E-02 |
| STAD | shared | mRNA | FHL1 | 1.086663699 | 3.13E-02 |
| STAD | shared | mRNA | ADGRB3 | 1.118250767 | 2.99E-03 |
| STAD | shared | mRNA | AR | 1.118999291 | 9.39E-03 |
| STAD | shared | mRNA | SRPX | 1.145780619 | 3.25E-03 |
| STAD | shared | mRNA | CFL2 | 1.15517939 | 6.94E-03 |
| STAD | shared | mRNA | MMRN1 | 1.160009448 | 5.37E-04 |
| STAD | shared | mRNA | SLC8A1 | 1.165891253 | 1.92E-02 |
| STAD | shared | mRNA | ZFPM2 | 1.181402743 | 1.39E-03 |
| STAD | shared | mRNA | FERMT2 | 1.197597768 | 1.06E-03 |
| STAD | specific | mRNA | POLD3 | 0.763783747 | 1.34E-02 |
| STAD | specific | mRNA | PCDHA11 | 1.074663673 | 1.18E-02 |
| STAD | specific | mRNA | PCDHA12 | 1.088725411 | 3.55E-03 |
| STAD | specific | mRNA | PNMA2 | 1.098554759 | 4.10E-02 |
| STAD | specific | mRNA | OPALIN | 1.113108482 | 4.05E-02 |
| STAD | specific | mRNA | COL6A3 | 1.160587485 | 2.20E-02 |
| STAD | semi-shared | mRNA | QSOX2 | 0.758616576 | 2.65E-02 |
| STAD | semi-shared | mRNA | TMEM63A | 0.773520379 | 1.77E-02 |
| STAD | semi-shared | mRNA | ARHGEF39 | 0.790452235 | 5.48E-03 |
| STAD | semi-shared | mRNA | TNFRSF10B | 0.807597563 | 3.94E-02 |
| STAD | semi-shared | mRNA | SOX9 | 0.809810329 | 3.59E-03 |
| STAD | semi-shared | mRNA | TYMS | 0.833193455 | 4.50E-02 |
| STAD | semi-shared | mRNA | SLC1A5 | 0.856855688 | 4.44E-02 |
| STAD | semi-shared | mRNA | IGF2BP1 | 1.057649968 | 1.35E-02 |
| STAD | semi-shared | mRNA | ALB | 1.058436038 | 1.23E-02 |
| STAD | semi-shared | mRNA | DKK1 | 1.06080002 | 7.66E-03 |
| STAD | semi-shared | mRNA | RGMA | 1.070191855 | 4.86E-02 |
| STAD | semi-shared | mRNA | TRIM71 | 1.071140424 | 1.61E-02 |
| STAD | semi-shared | mRNA | CSMD1 | 1.073043019 | 2.12E-02 |
| STAD | semi-shared | mRNA | PRKAA2 | 1.084499955 | 7.30E-03 |
| STAD | semi-shared | mRNA | CADM2 | 1.097261766 | 4.41E-03 |
| STAD | semi-shared | mRNA | CAMK2A | 1.101370333 | 4.49E-02 |
| STAD | semi-shared | mRNA | PI15 | 1.10470225 | 5.86E-03 |
| STAD | semi-shared | mRNA | PGR | 1.107458813 | 2.47E-02 |
| STAD | semi-shared | mRNA | EPHA5 | 1.109655914 | 2.17E-02 |
| STAD | semi-shared | mRNA | SYT14 | 1.114526685 | 6.49E-03 |
| STAD | semi-shared | mRNA | SYNDIG1 | 1.119199018 | 2.94E-02 |
| STAD | semi-shared | mRNA | AGT | 1.119499427 | 1.43E-02 |
| STAD | semi-shared | mRNA | COL8A1 | 1.126376765 | 1.12E-02 |
| STAD | semi-shared | mRNA | NPR3 | 1.136142968 | 2.31E-03 |
| STAD | semi-shared | mRNA | RAB3B | 1.140330691 | 3.19E-03 |
| STAD | semi-shared | mRNA | BCAT1 | 1.142010002 | 1.92E-02 |
| STAD | semi-shared | mRNA | INHBA | 1.148544106 | 1.81E-02 |
| STAD | semi-shared | mRNA | SLC35F1 | 1.156362019 | 4.06E-03 |
| STAD | semi-shared | mRNA | PDE3A | 1.15775737 | 4.95E-03 |
| STAD | semi-shared | mRNA | CLIP3 | 1.159337552 | 6.28E-03 |
| STAD | semi-shared | mRNA | EPB41L3 | 1.170152915 | 2.60E-02 |
| STAD | semi-shared | mRNA | P2RY6 | 1.182179571 | 2.48E-02 |
| STAD | semi-shared | mRNA | PLPPR4 | 1.19562815 | 8.72E-04 |
| STAD | semi-shared | mRNA | SOX5 | 1.207507133 | 3.16E-03 |
| STAD | semi-shared | mRNA | KLF9 | 1.207748055 | 9.30E-03 |
| STAD | semi-shared | mRNA | FRMD6 | 1.21871476 | 2.06E-03 |
| STAD | semi-shared | mRNA | SOCS3 | 1.224277373 | 1.64E-02 |
| STAD | semi-shared | mRNA | GABARAPL1 | 1.245754448 | 1.41E-02 |
| STAD | semi-shared | mRNA | COL4A1 | 1.259703931 | 9.53E-03 |
| STAD | shared | miRNA | hsa-miR-139-5p | 1.162447824 | 1.91E-02 |
| STAD | semi-shared | miRNA | hsa-miR-125b-5p | 1.113653145 | 4.83E-02 |
| STAD | semi-shared | miRNA | hsa-miR-125a-5p | 1.279614114 | 8.21E-03 |
| STAD | shared | lncRNA | PVT1 | 0.814280769 | 1.35E-02 |
| STAD | shared | lncRNA | ADAMTS9-AS2 | 1.14365553 | 4.39E-03 |
| STAD | shared | lncRNA | ADAMTS9-AS1 | 1.224320154 | 1.87E-04 |
| STAD | specific | lncRNA | LINC00454 | 1.124865894 | 1.93E-02 |
| STAD | specific | lncRNA | LINC00411 | 1.157093583 | 2.50E-02 |
| STAD | specific | lncRNA | LINC00507 | 1.114216422 | 2.85E-02 |
| STAD | specific | lncRNA | LINC00452 | 1.186535993 | 6.32E-03 |
| STAD | semi-shared | lncRNA | TPTEP1 | 1.159031783 | 5.69E-03 |
| STAD | semi-shared | lncRNA | SNHG14 | 1.11184368 | 4.57E-02 |
| STAD | semi-shared | lncRNA | CT62 | 0.894178002 | 4.00E-02 |
| STAD | semi-shared | lncRNA | PCA3 | 1.076864505 | 4.37E-02 |
| STAD | semi-shared | lncRNA | JAZF1-AS1 | 1.155782087 | 2.64E-02 |
| STAD | semi-shared | lncRNA | LINC00461 | 1.12476879 | 1.37E-02 |
| STAD | semi-shared | lncRNA | FRMD6-AS2 | 1.164226121 | 3.22E-03 |
| STAD | semi-shared | lncRNA | GOLGA8IP | 1.154908147 | 5.99E-03 |
| STAD | semi-shared | lncRNA | CCNYL2 | 1.094620662 | 1.61E-02 |
| STAD | semi-shared | lncRNA | KIF25-AS1 | 1.080624046 | 2.20E-02 |
| STAD | semi-shared | lncRNA | DLEU2 | 0.785187371 | 2.59E-02 |
| STAD | semi-shared | lncRNA | LINC00485 | 1.173153062 | 1.49E-02 |
| STAD | semi-shared | lncRNA | ST7-AS2 | 1.147078722 | 4.32E-02 |
| STAD | semi-shared | lncRNA | DSCR10 | 1.245504948 | 1.13E-03 |
| STAD | semi-shared | lncRNA | LSAMP-AS1 | 1.109130328 | 2.79E-02 |
| STAD | semi-shared | lncRNA | ARHGEF38-IT1 | 0.879846707 | 1.74E-02 |
| LIHC | shared | mRNA | FERMT2 | 0.773382164 | 1.39E-02 |
| LIHC | shared | mRNA | ARHGEF26 | 0.881043027 | 9.67E-03 |
| LIHC | shared | mRNA | AR | 0.897683784 | 3.24E-03 |
| LIHC | shared | mRNA | CXCL12 | 0.905279295 | 4.38E-02 |
| LIHC | shared | mRNA | HMGA2 | 1.068443349 | 2.24E-02 |
| LIHC | shared | mRNA | LIN28B | 1.0753832 | 3.45E-03 |
| LIHC | shared | mRNA | ZIC5 | 1.079863968 | 1.41E-02 |
| LIHC | shared | mRNA | E2F7 | 1.128464178 | 1.86E-02 |
| LIHC | shared | mRNA | LRP8 | 1.188611788 | 2.02E-03 |
| LIHC | shared | mRNA | TOP2A | 1.230334858 | 3.01E-04 |
| LIHC | shared | mRNA | TEDC2 | 1.242791048 | 9.16E-04 |
| LIHC | shared | mRNA | SAPCD2 | 1.247334168 | 7.52E-05 |
| LIHC | shared | mRNA | CDCA5 | 1.289711049 | 1.55E-04 |
| LIHC | shared | mRNA | DSN1 | 1.301352494 | 2.07E-02 |
| LIHC | shared | mRNA | GINS1 | 1.321950274 | 3.26E-05 |
| LIHC | shared | mRNA | CEP55 | 1.365381262 | 1.23E-06 |
| LIHC | shared | mRNA | CCNB1 | 1.367426398 | 1.37E-05 |
| LIHC | shared | mRNA | LPCAT1 | 1.374873369 | 1.50E-06 |
| LIHC | shared | mRNA | CCNF | 1.395482534 | 1.22E-04 |
| LIHC | shared | mRNA | RACGAP1 | 1.459027988 | 3.70E-05 |
| LIHC | shared | mRNA | CDCA8 | 1.477911765 | 1.28E-07 |
| LIHC | shared | mRNA | KPNA2 | 1.788655666 | 2.94E-08 |
| LIHC | specific | mRNA | N4BP2L1 | 0.677930904 | 1.47E-05 |
| LIHC | specific | mRNA | ETS2 | 0.747570514 | 1.55E-03 |
| LIHC | specific | mRNA | CDC37L1 | 0.749596738 | 5.89E-03 |
| LIHC | specific | mRNA | DUSP10 | 0.750963476 | 7.81E-04 |
| LIHC | specific | mRNA | PANK1 | 0.76067706 | 5.87E-04 |
| LIHC | specific | mRNA | SERPING1 | 0.769232619 | 5.16E-04 |
| LIHC | specific | mRNA | LDLR | 0.771044944 | 6.91E-03 |
| LIHC | specific | mRNA | MYO1B | 0.795439724 | 7.84E-03 |
| LIHC | specific | mRNA | MMUT | 0.806753517 | 1.36E-02 |
| LIHC | specific | mRNA | PPP1R3B | 0.836296846 | 7.10E-03 |
| LIHC | specific | mRNA | ESR1 | 0.874240776 | 8.38E-04 |
| LIHC | specific | mRNA | ACSL1 | 0.878985868 | 1.22E-02 |
| LIHC | specific | mRNA | CD69 | 0.88432005 | 1.67E-02 |
| LIHC | specific | mRNA | ALB | 0.913643144 | 1.15E-02 |
| LIHC | specific | mRNA | SAMD12 | 1.095696914 | 2.35E-02 |
| LIHC | specific | mRNA | WEE1 | 1.211201838 | 4.35E-02 |
| LIHC | semi-shared | mRNA | NDRG2 | 0.710327142 | 1.51E-04 |
| LIHC | semi-shared | mRNA | SMIM14 | 0.712587114 | 1.14E-04 |
| LIHC | semi-shared | mRNA | RCAN1 | 0.770649714 | 2.89E-03 |
| LIHC | semi-shared | mRNA | DLC1 | 0.775536653 | 5.01E-03 |
| LIHC | semi-shared | mRNA | GABARAPL1 | 0.782112255 | 5.39E-04 |
| LIHC | semi-shared | mRNA | CPEB3 | 0.797376835 | 8.08E-04 |
| LIHC | semi-shared | mRNA | RNF152 | 0.833897336 | 2.02E-02 |
| LIHC | semi-shared | mRNA | PLPP3 | 0.847023406 | 3.01E-02 |
| LIHC | semi-shared | mRNA | SH2D1B | 0.863307249 | 5.00E-02 |
| LIHC | semi-shared | mRNA | SGK1 | 0.870037205 | 2.80E-02 |
| LIHC | semi-shared | mRNA | SLC7A2 | 0.889210125 | 2.18E-02 |
| LIHC | semi-shared | mRNA | SLC46A3 | 0.89773938 | 1.23E-02 |
| LIHC | shared | miRNA | hsa-miR-139-5p | 0.716749241 | 1.16E-07 |
| LIHC | shared | lncRNA | WASIR2 | 1.21766326 | 3.82E-03 |
| LIHC | specific | lncRNA | C3P1 | 0.915982987 | 2.43E-03 |
| LIHC | semi-shared | lncRNA | LINC00402 | 0.820363922 | 1.59E-04 |
| LIHC | semi-shared | lncRNA | LINC00261 | 0.90946381 | 3.77E-02 |
| COAD | shared | mRNA | CCNF | 0.673839831 | 1.40E-02 |
| COAD | shared | mRNA | KPNA2 | 0.64776324 | 1.77E-02 |
| COAD | shared | mRNA | DSN1 | 0.698568657 | 2.90E-02 |
| COAD | specific | mRNA | ACOX1 | 0.556830965 | 2.50E-03 |
| COAD | specific | mRNA | ABCD3 | 0.621910752 | 3.94E-03 |
| COAD | specific | mRNA | RBM47 | 0.623446242 | 1.96E-02 |
| COAD | specific | mRNA | MTM1 | 0.630018282 | 7.49E-03 |
| COAD | specific | mRNA | ABHD5 | 0.653916153 | 3.40E-02 |
| COAD | specific | mRNA | CCNYL1 | 0.660183962 | 4.81E-02 |
| COAD | specific | mRNA | GAB1 | 0.715788307 | 4.50E-02 |
| COAD | specific | mRNA | HIGD1A | 0.719879951 | 1.62E-02 |
| COAD | specific | mRNA | GNG12 | 0.721050587 | 2.25E-02 |
| COAD | specific | mRNA | KIF13B | 0.764444308 | 3.14E-02 |
| COAD | specific | mRNA | VWA5A | 0.826740681 | 3.03E-02 |
| COAD | specific | mRNA | ZC3H12C | 0.864901319 | 3.74E-02 |
| COAD | specific | mRNA | DIRAS2 | 1.150135309 | 4.65E-02 |
| COAD | specific | mRNA | ADAM11 | 1.218109753 | 1.49E-02 |
| COAD | specific | mRNA | OSBPL1A | 1.258271681 | 2.18E-02 |
| COAD | specific | mRNA | RIMKLB | 1.262298488 | 4.33E-03 |
| COAD | semi-shared | mRNA | SLC35D1 | 0.671295623 | 7.16E-03 |
| COAD | semi-shared | mRNA | AGT | 0.869757418 | 4.81E-02 |
| COAD | semi-shared | mRNA | MYPN | 0.877168938 | 4.50E-02 |
| COAD | semi-shared | mRNA | SLC4A4 | 0.906353486 | 1.61E-02 |
| COAD | semi-shared | mRNA | ACSL6 | 0.930769885 | 3.41E-02 |
| COAD | semi-shared | mRNA | DTNA | 1.122355506 | 3.77E-02 |
| COAD | semi-shared | mRNA | NRCAM | 1.140164411 | 1.83E-02 |
| COAD | semi-shared | mRNA | POU4F1 | 1.166343455 | 5.60E-03 |
| COAD | semi-shared | mRNA | RGS16 | 1.216768291 | 4.89E-02 |
| COAD | semi-shared | mRNA | ARL6IP4 | 1.38298267 | 1.76E-03 |
| COAD | semi-shared | mRNA | OSBPL3 | 1.408181987 | 3.63E-02 |
| COAD | semi-shared | mRNA | DMPK | 1.45091643 | 3.80E-03 |
| COAD | semi-shared | mRNA | LIMK1 | 1.607167367 | 6.50E-03 |
| COAD | semi-shared | mRNA | QSOX2 | 1.768673358 | 4.38E-03 |
| COAD | specific | miRNA | hsa-miR-193a-3p | 1.389629688 | 4.28E-03 |
| COAD | shared | lncRNA | WASIR2 | 1.145649201 | 3.07E-02 |
| COAD | shared | lncRNA | LINC00534 | 1.150740877 | 3.87E-02 |
| COAD | semi-shared | lncRNA | LINC00523 | 0.676661521 | 3.81E-02 |
| COAD | semi-shared | lncRNA | MYO16-AS1 | 1.139945806 | 4.09E-02 |
| COAD | semi-shared | lncRNA | WT1-AS | 1.144048317 | 1.81E-02 |
| COAD | semi-shared | lncRNA | NSUN5P2 | 1.197057599 | 1.34E-02 |
| COAD | semi-shared | lncRNA | ALMS1-IT1 | 1.227800561 | 1.88E-02 |

**Table S6**. The results of RNA correlation analysis.

| Cancer types | pair types | mRNA | miRNA | Coefficient-R | p-value |
| --- | --- | --- | --- | --- | --- |
| ESCA | mRNA-miRNA | SNX10 | hsa-miR-363-3p | 0.021 | 7.92E-01 |
| ESCA | mRNA-miRNA | RGS16 | hsa-miR-129-5p | 0.201 | 1.05E-02 |
| ESCA | mRNA-miRNA | HMGB3 | hsa-miR-125a-5p | 0.038 | 6.35E-01 |
| STAD | mRNA-miRNA | **HMGA2** | **hsa-miR-490-3p** | **-0.106** | **4.19E-02** |
| STAD | mRNA-miRNA | COL6A3 | hsa-miR-23b-3p | 0.231 | 6.48E-06 |
| STAD | mRNA-miRNA | AGT | hsa-miR-129-5p | 0.299 | 4.02E-09 |
| STAD | mRNA-miRNA | COL8A1 | hsa-miR-129-5p | 0.147 | 4.43E-03 |
| STAD | mRNA-miRNA | INHBA | hsa-miR-129-5p | -0.027 | 6.01E-01 |
| STAD | mRNA-miRNA | P2RY6 | hsa-miR-129-5p | -0.041 | 4.36E-01 |
| STAD | mRNA-miRNA | COL4A1 | hsa-miR-129-5p | 0.024 | 6.39E-01 |
| STAD | mRNA-miRNA | COL4A1 | hsa-miR-125b-5p | 0.146 | 4.76E-03 |
| STAD | mRNA-miRNA | COL4A1 | hsa-miR-125a-5p | 0.049 | 3.43E-01 |
| LIHC | mRNA-miRNA | **PPP1R3B** | **hsa-miR-129-5p** | **-0.134** | **9.93E-03** |
| LIHC | mRNA-miRNA | **ACSL1** | **hsa-miR-129-5p** | **-0.19** | **2.43E-04** |
| LIHC | mRNA-miRNA | N4BP2L1 | hsa-miR-135a-5p | -0.089 | 8.84E-02 |
| LIHC | mRNA-miRNA | **TOP2A** | **hsa-miR-139-5p** | **-0.472** | **6.00E-22** |
| LIHC | mRNA-miRNA | **DSN1** | **hsa-miR-139-5p** | **-0.351** | **3.63E-12** |
| LIHC | mRNA-miRNA | **GINS1** | **hsa-miR-139-5p** | **-0.472** | **5.98E-22** |
| LIHC | mRNA-miRNA | **CCNB1** | **hsa-miR-139-5p** | **-0.547** | **2.69E-30** |
| LIHC | mRNA-miRNA | **RACGAP1** | **hsa-miR-139-5p** | **-0.431** | **3.84E-18** |
| LIHC | mRNA-miRNA | **CDCA8** | **hsa-miR-139-5p** | **-0.513** | **3.18E-26** |
| LIHC | mRNA-miRNA | **KPNA2** | **hsa-miR-139-5p** | **-0.49** | **8.91E-24** |
| LIHC | mRNA-miRNA | RCAN1 | hsa-miR-206 | 0.089 | 8.81E-02 |
| LIHC | mRNA-miRNA | SLC7A2 | hsa-miR-206 | -0.033 | 5.25E-01 |
| LIHC | mRNA-miRNA | SLC46A3 | hsa-miR-206 | -0.003 | 9.52E-01 |
| LIHC | mRNA-miRNA | PPP1R3B | hsa-miR-20b-5p | 0.013 | 8.01E-01 |
| LIHC | mRNA-miRNA | ESR1 | hsa-miR-20b-5p | -0.072 | 1.70E-01 |
| LIHC | mRNA-miRNA | CPEB3 | hsa-miR-216b-5p | 0.075 | 1.50E-01 |
| LIHC | mRNA-miRNA | **NDRG2** | **hsa-miR-338-3p** | **-0.122** | **1.85E-02** |
| LIHC | mRNA-miRNA | RNF152 | hsa-miR-338-3p | -0.007 | 8.87E-01 |
| LIHC | mRNA-miRNA | SLC7A2 | hsa-miR-338-3p | -0.032 | 5.41E-01 |
| LIHC | mRNA-miRNA | **CDCA5** | **hsa-miR-490-3p** | **-0.154** | **2.94E-03** |
| COAD | mRNA-miRNA | RGS16 | hsa-miR-129-5p | -0.014 | 7.64E-01 |
| COAD | mRNA-miRNA | OSBPL3 | hsa-miR-129-5p | 0.116 | 1.37E-02 |
| COAD | mRNA-miRNA | LIMK1 | hsa-miR-125b-5p | 0.238 | 3.08E-07 |
| COAD | mRNA-miRNA | LIMK1 | hsa-miR-125a-5p | 0.154 | 1.02E-03 |
| COAD | mRNA-miRNA | **GAB1** | **hsa-miR-142-3p** | **-0.291** | **3.08E-10** |
| COAD | mRNA-miRNA | GAB1 | hsa-miR-27a-3p | 0.045 | 3.39E-01 |
| COAD | mRNA-miRNA | GAB1 | hsa-miR-429 | -0.055 | 2.47E-01 |
| COAD | mRNA-miRNA | SLC4A4 | hsa-miR-338-3p | 0.034 | 4.67E-01 |
| STAD | miRNA-lncRNA | hsa-miR-490-3p | DSCR9 | -0.065 | 2.12E-01 |
| STAD | miRNA-lncRNA | hsa-miR-490-3p | WASIR2 | 0.031 | 5.51E-01 |
| STAD | miRNA-lncRNA | hsa-miR-490-3p | DLX6-AS1 | -0.089 | 8.59E-02 |
| STAD | miRNA-lncRNA | **hsa-miR-490-3p** | **PVT1** | **-0.124** | **1.69E-02** |
| LIHC | miRNA-lncRNA | hsa-miR-129-5p | STEAP3-AS1 | -0.02 | 6.95E-01 |
| LIHC | miRNA-lncRNA | hsa-miR-129-5p | NPHP3-AS1 | 0.147 | 4.74E-03 |
| LIHC | miRNA-lncRNA | **hsa-miR-139-5p** | **DLX6-AS1** | **-0.183** | **4.07E-04** |
| LIHC | miRNA-lncRNA | **hsa-miR-139-5p** | **PVT1** | **-0.24** | **2.94E-06** |
| LIHC | miRNA-lncRNA | **hsa-miR-139-5p** | **LINC00534** | **-0.195** | **1.63E-04** |
| LIHC | miRNA-lncRNA | hsa-miR-338-3p | WDFY3-AS2 | -0.03 | 5.62E-01 |
| LIHC | miRNA-lncRNA | hsa-miR-338-3p | KRT16P3 | -0.08 | 1.23E-01 |
| LIHC | miRNA-lncRNA | hsa-miR-338-3p | LINC00365 | 0.125 | 1.60E-02 |
| LIHC | miRNA-lncRNA | **hsa-miR-338-3p** | **LINC00402** | **-0.203** | **8.09E-05** |
| LIHC | miRNA-lncRNA | hsa-miR-338-3p | ST3GAL6-AS1 | -0.08 | 1.27E-01 |
| LIHC | miRNA-lncRNA | hsa-miR-338-3p | PWRN1 | -0.03 | 5.71E-01 |
| LIHC | miRNA-lncRNA | hsa-miR-338-3p | LINC00261 | -0.094 | 7.09E-02 |
| LIHC | miRNA-lncRNA | **hsa-miR-490-3p** | **DSCR9** | **-0.103** | **4.82E-02** |
| LIHC | miRNA-lncRNA | **hsa-miR-490-3p** | **WASIR2** | **-0.124** | **1.67E-02** |
| LIHC | miRNA-lncRNA | hsa-miR-490-3p | DLX6-AS1 | -0.102 | 5.08E-02 |
| LIHC | miRNA-lncRNA | hsa-miR-490-3p | PVT1 | -0.094 | 7.07E-02 |
| COAD | miRNA-lncRNA | **hsa-miR-142-3p** | **STRCP1** | **-0.158** | **7.69E-04** |
| COAD | miRNA-lncRNA | hsa-miR-142-3p | LINC00324 | 0.178 | 1.51E-04 |
| COAD | miRNA-lncRNA | **hsa-miR-142-3p** | **LINC00488** | **-0.138** | **3.44E-03** |
| COAD | miRNA-lncRNA | hsa-miR-142-3p | LY86-AS1 | 0.025 | 5.98E-01 |
| STAD | lncRNA-mRNA | **PVT1** | **HMGA2** | **0.208** | **5.12E-05** |
| LIHC | lncRNA-mRNA | **DLX6-AS1** | **TOP2A** | **0.286** | **1.86E-08** |
| LIHC | lncRNA-mRNA | **DLX6-AS1** | **DSN1** | **0.24** | **2.75E-06** |
| LIHC | lncRNA-mRNA | **DLX6-AS1** | **GINS1** | **0.322** | **1.86E-10** |
| LIHC | lncRNA-mRNA | **DLX6-AS1** | **CCNB1** | **0.306** | **1.57E-09** |
| LIHC | lncRNA-mRNA | **DLX6-AS1** | **RACGAP1** | **0.326** | **1.09E-10** |
| LIHC | lncRNA-mRNA | **DLX6-AS1** | **CDCA8** | **0.247** | **1.35E-06** |
| LIHC | lncRNA-mRNA | **DLX6-AS1** | **KPNA2** | **0.274** | **7.15E-08** |
| LIHC | lncRNA-mRNA | **PVT1** | **TOP2A** | **0.133** | **1.03E-02** |
| LIHC | lncRNA-mRNA | PVT1 | DSN1 | 0.008 | 8.81E-01 |
| LIHC | lncRNA-mRNA | **PVT1** | **GINS1** | **0.126** | **1.48E-02** |
| LIHC | lncRNA-mRNA | **PVT1** | **CCNB1** | **0.264** | **2.12E-07** |
| LIHC | lncRNA-mRNA | **PVT1** | **RACGAP1** | **0.19** | **2.16E-04** |
| LIHC | lncRNA-mRNA | **PVT1** | **CDCA8** | **0.172** | **8.35E-04** |
| LIHC | lncRNA-mRNA | **PVT1** | **KPNA2** | **0.287** | **1.51E-08** |
| LIHC | lncRNA-mRNA | **LINC00534** | **TOP2A** | **0.224** | **1.21E-05** |
| LIHC | lncRNA-mRNA | **LINC00534** | **DSN1** | **0.147** | **4.45E-03** |
| LIHC | lncRNA-mRNA | **LINC00534** | **GINS1** | **0.226** | **1.02E-05** |
| LIHC | lncRNA-mRNA | **LINC00534** | **CCNB1** | **0.239** | **2.94E-06** |
| LIHC | lncRNA-mRNA | **LINC00534** | **RACGAP1** | **0.211** | **3.93E-05** |
| LIHC | lncRNA-mRNA | **LINC00534** | **CDCA8** | **0.275** | **6.13E-08** |
| LIHC | lncRNA-mRNA | **LINC00534** | **KPNA2** | **0.214** | **3.07E-05** |
| LIHC | lncRNA-mRNA | **LINC00402** | **NDRG2** | **0.29** | **1.05E-08** |
| LIHC | lncRNA-mRNA | **DSCR9** | **CDCA5** | **0.445** | **1.32E-19** |
| LIHC | lncRNA-mRNA | **WASIR2** | **CDCA5** | **0.406** | **2.66E-16** |
| COAD | lncRNA-mRNA | **STRCP1** | **GAB1** | **0.225** | **7.67E-07** |
| COAD | lncRNA-mRNA | **LINC00488** | **GAB1** | **0.225** | **7.85E-07** |

**Table S7.** ESCA related-ceRNA network nodes(after threshold adjustment).

| node1 | node2 | line | line-types |
| --- | --- | --- | --- |
| DSCR9 | hsa-miR-490-3p | lncRNA-up_miRNA-down | shared |
| WASIR2 | hsa-miR-490-3p | lncRNA-up_miRNA-down | shared |
| DLX6-AS1 | hsa-miR-490-3p | lncRNA-up_miRNA-down | shared |
| PVT1 | hsa-miR-490-3p | lncRNA-up_miRNA-down | shared |
| DLX6-AS1 | hsa-miR-139-5p | lncRNA-up_miRNA-down | shared |
| PVT1 | hsa-miR-139-5p | lncRNA-up_miRNA-down | shared |
| LINC00534 | hsa-miR-139-5p | lncRNA-up_miRNA-down | shared |
| hsa-miR-490-3p | CEP55 | miRNA-down_mRNA-up | shared |
| hsa-miR-490-3p | CDCA5 | miRNA-down_mRNA-up | shared |
| hsa-miR-490-3p | HMGA2 | miRNA-down_mRNA-up | shared |
| hsa-miR-490-3p | ZIC5 | miRNA-down_mRNA-up | shared |
| hsa-miR-490-3p | COL4A1 | miRNA-down_mRNA-up | shared |
| hsa-miR-490-3p | RFC3 | miRNA-down_mRNA-up | shared |
| hsa-miR-490-3p | PRC1 | miRNA-down_mRNA-up | shared |
| hsa-miR-490-3p | SOX4 | miRNA-down_mRNA-up | shared |
| hsa-miR-490-3p | LIN28B | miRNA-down_mRNA-up | shared |
| hsa-miR-490-3p | SAPCD2 | miRNA-down_mRNA-up | shared |
| hsa-miR-139-5p | LRP8 | miRNA-down_mRNA-up | shared |
| hsa-miR-139-5p | CDCA8 | miRNA-down_mRNA-up | shared |
| hsa-miR-139-5p | RACGAP1 | miRNA-down_mRNA-up | shared |
| hsa-miR-139-5p | E2F7 | miRNA-down_mRNA-up | shared |
| hsa-miR-139-5p | PRC1 | miRNA-down_mRNA-up | shared |
| hsa-miR-139-5p | CCNF | miRNA-down_mRNA-up | shared |
| hsa-miR-139-5p | TEDC2 | miRNA-down_mRNA-up | shared |
| hsa-miR-139-5p | TOP2A | miRNA-down_mRNA-up | shared |
| hsa-miR-139-5p | NME1 | miRNA-down_mRNA-up | shared |
| hsa-miR-139-5p | KPNA2 | miRNA-down_mRNA-up | shared |
| hsa-miR-139-5p | DSN1 | miRNA-down_mRNA-up | shared |
| hsa-miR-139-5p | GINS1 | miRNA-down_mRNA-up | shared |
| hsa-miR-139-5p | LPCAT1 | miRNA-down_mRNA-up | shared |
| hsa-miR-139-5p | ITGA2 | miRNA-down_mRNA-up | shared |
| hsa-miR-139-5p | CCNB1 | miRNA-down_mRNA-up | shared |
| hsa-miR-139-5p | OSBPL3 | miRNA-down_mRNA-up | shared |
| hsa-miR-139-5p | SLC6A14 | miRNA-down_mRNA-up | shared |
| ADAMTS9-AS1 | hsa-miR-301b-3p | lncRNA-down_miRNA-up | shared |
| ADAMTS9-AS2 | hsa-miR-301b-3p | lncRNA-down_miRNA-up | shared |
| ADAMTS9-AS1 | hsa-miR-146b-5p | lncRNA-down_miRNA-up | shared |
| hsa-miR-301b-3p | TENT5C | miRNA-up_mRNA-down | shared |
| hsa-miR-301b-3p | CXCL12 | miRNA-up_mRNA-down | shared |
| hsa-miR-301b-3p | LRRK2 | miRNA-up_mRNA-down | shared |
| hsa-miR-301b-3p | SPART | miRNA-up_mRNA-down | shared |
| hsa-miR-301b-3p | CFL2 | miRNA-up_mRNA-down | shared |
| hsa-miR-301b-3p | FERMT2 | miRNA-up_mRNA-down | shared |
| hsa-miR-301b-3p | FOXF1 | miRNA-up_mRNA-down | shared |
| hsa-miR-301b-3p | SLC8A1 | miRNA-up_mRNA-down | shared |
| hsa-miR-301b-3p | EIF4E3 | miRNA-up_mRNA-down | shared |
| hsa-miR-301b-3p | ARHGEF26 | miRNA-up_mRNA-down | shared |
| hsa-miR-301b-3p | ADGRB3 | miRNA-up_mRNA-down | shared |
| hsa-miR-301b-3p | CHRM2 | miRNA-up_mRNA-down | shared |
| hsa-miR-301b-3p | ZFPM2 | miRNA-up_mRNA-down | shared |
| hsa-miR-301b-3p | KLF4 | miRNA-up_mRNA-down | shared |
| hsa-miR-301b-3p | SRPX | miRNA-up_mRNA-down | shared |
| hsa-miR-301b-3p | AR | miRNA-up_mRNA-down | shared |
| hsa-miR-146b-5p | MMRN1 | miRNA-up_mRNA-down | shared |
| hsa-miR-146b-5p | LIFR | miRNA-up_mRNA-down | shared |
| hsa-miR-146b-5p | FHL1 | miRNA-up_mRNA-down | shared |
| BTN2A3P | hsa-miR-338-3p | lncRNA-up_miRNA-down | specific |
| PMS2P3 | hsa-miR-338-3p | lncRNA-up_miRNA-down | specific |
| SDHAP1 | hsa-miR-338-3p | lncRNA-up_miRNA-down | specific |
| CA5BP1 | hsa-miR-338-3p | lncRNA-up_miRNA-down | specific |
| KTN1-AS1 | hsa-miR-338-3p | lncRNA-up_miRNA-down | specific |
| LINC00243 | hsa-miR-338-3p | lncRNA-up_miRNA-down | specific |
| GBP1P1 | hsa-miR-338-3p | lncRNA-up_miRNA-down | specific |
| MIAT | hsa-miR-338-3p | lncRNA-up_miRNA-down | specific |
| LINC00310 | hsa-miR-338-3p | lncRNA-up_miRNA-down | specific |
| HOTAIRM1 | hsa-miR-338-3p | lncRNA-up_miRNA-down | specific |
| MIR155HG | hsa-miR-338-3p | lncRNA-up_miRNA-down | specific |
| SOX2-OT | hsa-miR-338-3p | lncRNA-up_miRNA-down | specific |
| TIPARP-AS1 | hsa-miR-338-3p | lncRNA-up_miRNA-down | specific |
| SIAH2-AS1 | hsa-miR-338-3p | lncRNA-up_miRNA-down | specific |
| MTUS2-AS1 | hsa-miR-10a-5p | lncRNA-up_miRNA-down | specific |
| RP9P | hsa-miR-10a-5p | lncRNA-up_miRNA-down | specific |
| HCP5 | hsa-miR-10a-5p | lncRNA-up_miRNA-down | specific |
| LINC00243 | hsa-miR-10a-5p | lncRNA-up_miRNA-down | specific |
| GBP1P1 | hsa-miR-10a-5p | lncRNA-up_miRNA-down | specific |
| MIAT | hsa-miR-10a-5p | lncRNA-up_miRNA-down | specific |
| UBAC2-AS1 | hsa-miR-10a-5p | lncRNA-up_miRNA-down | specific |
| MIR155HG | hsa-miR-10a-5p | lncRNA-up_miRNA-down | specific |
| TIPARP-AS1 | hsa-miR-10a-5p | lncRNA-up_miRNA-down | specific |
| LINC00243 | hsa-miR-135a-5p | lncRNA-up_miRNA-down | specific |
| HOTAIRM1 | hsa-miR-135a-5p | lncRNA-up_miRNA-down | specific |
| SOX2-OT | hsa-miR-135a-5p | lncRNA-up_miRNA-down | specific |
| hsa-miR-338-3p | CLSTN1 | miRNA-down_mRNA-up | specific |
| hsa-miR-338-3p | MYCL | miRNA-down_mRNA-up | specific |
| hsa-miR-338-3p | TPM3 | miRNA-down_mRNA-up | specific |
| hsa-miR-338-3p | TAGLN2 | miRNA-down_mRNA-up | specific |
| hsa-miR-338-3p | IPO9 | miRNA-down_mRNA-up | specific |
| hsa-miR-338-3p | IFIT2 | miRNA-down_mRNA-up | specific |
| hsa-miR-338-3p | BUB3 | miRNA-down_mRNA-up | specific |
| hsa-miR-338-3p | SLC43A3 | miRNA-down_mRNA-up | specific |
| hsa-miR-338-3p | COA4 | miRNA-down_mRNA-up | specific |
| hsa-miR-338-3p | RRM1 | miRNA-down_mRNA-up | specific |
| hsa-miR-338-3p | LDHA | miRNA-down_mRNA-up | specific |
| hsa-miR-338-3p | NEMP1 | miRNA-down_mRNA-up | specific |
| hsa-miR-338-3p | TMTC3 | miRNA-down_mRNA-up | specific |
| hsa-miR-338-3p | LCP1 | miRNA-down_mRNA-up | specific |
| hsa-miR-338-3p | TFDP1 | miRNA-down_mRNA-up | specific |
| hsa-miR-338-3p | HIF1A | miRNA-down_mRNA-up | specific |
| hsa-miR-338-3p | AHSA1 | miRNA-down_mRNA-up | specific |
| hsa-miR-338-3p | FURIN | miRNA-down_mRNA-up | specific |
| hsa-miR-338-3p | CYB5B | miRNA-down_mRNA-up | specific |
| hsa-miR-338-3p | TP53 | miRNA-down_mRNA-up | specific |
| hsa-miR-338-3p | SRSF2 | miRNA-down_mRNA-up | specific |
| hsa-miR-338-3p | ZNF286A | miRNA-down_mRNA-up | specific |
| hsa-miR-338-3p | CNP | miRNA-down_mRNA-up | specific |
| hsa-miR-338-3p | ANAPC11 | miRNA-down_mRNA-up | specific |
| hsa-miR-338-3p | R3HDM4 | miRNA-down_mRNA-up | specific |
| hsa-miR-338-3p | MRPS12 | miRNA-down_mRNA-up | specific |
| hsa-miR-338-3p | ARL4C | miRNA-down_mRNA-up | specific |
| hsa-miR-338-3p | TET3 | miRNA-down_mRNA-up | specific |
| hsa-miR-338-3p | FKBP1A | miRNA-down_mRNA-up | specific |
| hsa-miR-338-3p | APMAP | miRNA-down_mRNA-up | specific |
| hsa-miR-338-3p | EIF6 | miRNA-down_mRNA-up | specific |
| hsa-miR-338-3p | KIAA0930 | miRNA-down_mRNA-up | specific |
| hsa-miR-338-3p | HMGXB4 | miRNA-down_mRNA-up | specific |
| hsa-miR-338-3p | TBL1XR1 | miRNA-down_mRNA-up | specific |
| hsa-miR-338-3p | MAGEF1 | miRNA-down_mRNA-up | specific |
| hsa-miR-338-3p | TFG | miRNA-down_mRNA-up | specific |
| hsa-miR-338-3p | DVL3 | miRNA-down_mRNA-up | specific |
| hsa-miR-338-3p | C5orf22 | miRNA-down_mRNA-up | specific |
| hsa-miR-338-3p | TNFAIP8 | miRNA-down_mRNA-up | specific |
| hsa-miR-338-3p | SFXN1 | miRNA-down_mRNA-up | specific |
| hsa-miR-338-3p | DNPH1 | miRNA-down_mRNA-up | specific |
| hsa-miR-338-3p | RPL7L1 | miRNA-down_mRNA-up | specific |
| hsa-miR-338-3p | DSE | miRNA-down_mRNA-up | specific |
| hsa-miR-338-3p | DCBLD1 | miRNA-down_mRNA-up | specific |
| hsa-miR-338-3p | HNRNPA2B1 | miRNA-down_mRNA-up | specific |
| hsa-miR-338-3p | PTPN12 | miRNA-down_mRNA-up | specific |
| hsa-miR-338-3p | DERL1 | miRNA-down_mRNA-up | specific |
| hsa-miR-338-3p | PTDSS1 | miRNA-down_mRNA-up | specific |
| hsa-miR-338-3p | CEP78 | miRNA-down_mRNA-up | specific |
| hsa-miR-338-3p | SET | miRNA-down_mRNA-up | specific |
| hsa-miR-338-3p | MSN | miRNA-down_mRNA-up | specific |
| hsa-miR-10a-5p | GCLM | miRNA-down_mRNA-up | specific |
| hsa-miR-10a-5p | ARPC5 | miRNA-down_mRNA-up | specific |
| hsa-miR-10a-5p | GATA3 | miRNA-down_mRNA-up | specific |
| hsa-miR-10a-5p | IFIT2 | miRNA-down_mRNA-up | specific |
| hsa-miR-10a-5p | SLC43A3 | miRNA-down_mRNA-up | specific |
| hsa-miR-10a-5p | PATL1 | miRNA-down_mRNA-up | specific |
| hsa-miR-10a-5p | PANX1 | miRNA-down_mRNA-up | specific |
| hsa-miR-10a-5p | STAT2 | miRNA-down_mRNA-up | specific |
| hsa-miR-10a-5p | ANKLE2 | miRNA-down_mRNA-up | specific |
| hsa-miR-10a-5p | CNOT2 | miRNA-down_mRNA-up | specific |
| hsa-miR-10a-5p | HIF1A | miRNA-down_mRNA-up | specific |
| hsa-miR-10a-5p | CRLF3 | miRNA-down_mRNA-up | specific |
| hsa-miR-10a-5p | SKA2 | miRNA-down_mRNA-up | specific |
| hsa-miR-10a-5p | H3-3B | miRNA-down_mRNA-up | specific |
| hsa-miR-10a-5p | EIF2AK2 | miRNA-down_mRNA-up | specific |
| hsa-miR-10a-5p | KCNS3 | miRNA-down_mRNA-up | specific |
| hsa-miR-10a-5p | SELENOI | miRNA-down_mRNA-up | specific |
| hsa-miR-10a-5p | MAP4K4 | miRNA-down_mRNA-up | specific |
| hsa-miR-10a-5p | BCL2L11 | miRNA-down_mRNA-up | specific |
| hsa-miR-10a-5p | CNOT9 | miRNA-down_mRNA-up | specific |
| hsa-miR-10a-5p | SLC5A3 | miRNA-down_mRNA-up | specific |
| hsa-miR-10a-5p | CNPY3 | miRNA-down_mRNA-up | specific |
| hsa-miR-10a-5p | ORC5 | miRNA-down_mRNA-up | specific |
| hsa-miR-10a-5p | ZNF273 | miRNA-down_mRNA-up | specific |
| hsa-miR-10a-5p | ARPC1A | miRNA-down_mRNA-up | specific |
| hsa-miR-10a-5p | CTSB | miRNA-down_mRNA-up | specific |
| hsa-miR-10a-5p | C9orf40 | miRNA-down_mRNA-up | specific |
| hsa-miR-135a-5p | GCLM | miRNA-down_mRNA-up | specific |
| hsa-miR-135a-5p | ADAR | miRNA-down_mRNA-up | specific |
| hsa-miR-135a-5p | MOV10 | miRNA-down_mRNA-up | specific |
| hsa-miR-135a-5p | DCLRE1B | miRNA-down_mRNA-up | specific |
| hsa-miR-135a-5p | NUDT5 | miRNA-down_mRNA-up | specific |
| hsa-miR-135a-5p | GATA3 | miRNA-down_mRNA-up | specific |
| hsa-miR-135a-5p | SGPL1 | miRNA-down_mRNA-up | specific |
| hsa-miR-135a-5p | PATL1 | miRNA-down_mRNA-up | specific |
| hsa-miR-135a-5p | PPFIA1 | miRNA-down_mRNA-up | specific |
| hsa-miR-135a-5p | OAS2 | miRNA-down_mRNA-up | specific |
| hsa-miR-135a-5p | LCP1 | miRNA-down_mRNA-up | specific |
| hsa-miR-135a-5p | GABPB1 | miRNA-down_mRNA-up | specific |
| hsa-miR-135a-5p | CMTM3 | miRNA-down_mRNA-up | specific |
| hsa-miR-135a-5p | C1QBP | miRNA-down_mRNA-up | specific |
| hsa-miR-135a-5p | SLFN11 | miRNA-down_mRNA-up | specific |
| hsa-miR-135a-5p | PPP1R9B | miRNA-down_mRNA-up | specific |
| hsa-miR-135a-5p | SLFN5 | miRNA-down_mRNA-up | specific |
| hsa-miR-135a-5p | KCNJ2 | miRNA-down_mRNA-up | specific |
| hsa-miR-135a-5p | MRPS12 | miRNA-down_mRNA-up | specific |
| hsa-miR-135a-5p | TMEM237 | miRNA-down_mRNA-up | specific |
| hsa-miR-135a-5p | KCNS3 | miRNA-down_mRNA-up | specific |
| hsa-miR-135a-5p | FOXN2 | miRNA-down_mRNA-up | specific |
| hsa-miR-135a-5p | SLC1A4 | miRNA-down_mRNA-up | specific |
| hsa-miR-135a-5p | TET3 | miRNA-down_mRNA-up | specific |
| hsa-miR-135a-5p | GPC1 | miRNA-down_mRNA-up | specific |
| hsa-miR-135a-5p | FKBP1A | miRNA-down_mRNA-up | specific |
| hsa-miR-135a-5p | APMAP | miRNA-down_mRNA-up | specific |
| hsa-miR-135a-5p | TBL1XR1 | miRNA-down_mRNA-up | specific |
| hsa-miR-135a-5p | HPS3 | miRNA-down_mRNA-up | specific |
| hsa-miR-135a-5p | FBXO45 | miRNA-down_mRNA-up | specific |
| hsa-miR-135a-5p | CEP135 | miRNA-down_mRNA-up | specific |
| hsa-miR-135a-5p | ZNF131 | miRNA-down_mRNA-up | specific |
| hsa-miR-135a-5p | YWHAG | miRNA-down_mRNA-up | specific |
| hsa-miR-135a-5p | ORC5 | miRNA-down_mRNA-up | specific |
| hsa-miR-135a-5p | DNAAF5 | miRNA-down_mRNA-up | specific |
| hsa-miR-135a-5p | ZNF107 | miRNA-down_mRNA-up | specific |
| hsa-miR-135a-5p | PHTF2 | miRNA-down_mRNA-up | specific |
| hsa-miR-135a-5p | ASAP1 | miRNA-down_mRNA-up | specific |
| hsa-miR-135a-5p | TNC | miRNA-down_mRNA-up | specific |
| hsa-miR-135a-5p | PSMB7 | miRNA-down_mRNA-up | specific |
| hsa-miR-135a-5p | SLC35A2 | miRNA-down_mRNA-up | specific |
| hsa-miR-135a-5p | PIM2 | miRNA-down_mRNA-up | specific |
| LINC00525 | hsa-miR-125a-5p | lncRNA-up_miRNA-down | semi-shared |
| C3P1 | hsa-miR-125a-5p | lncRNA-up_miRNA-down | semi-shared |
| DLEU1 | hsa-miR-125a-5p | lncRNA-up_miRNA-down | semi-shared |
| TMEM105 | hsa-miR-125a-5p | lncRNA-up_miRNA-down | semi-shared |
| KRT42P | hsa-miR-125a-5p | lncRNA-up_miRNA-down | semi-shared |
| SLC6A10P | hsa-miR-125a-5p | lncRNA-up_miRNA-down | semi-shared |
| LGALS8-AS1 | hsa-miR-125a-5p | lncRNA-up_miRNA-down | semi-shared |
| NAALADL2-AS2 | hsa-miR-125a-5p | lncRNA-up_miRNA-down | semi-shared |
| LINC00200 | hsa-miR-125a-5p | lncRNA-up_miRNA-down | semi-shared |
| UBE2Q1-AS1 | hsa-miR-125a-5p | lncRNA-up_miRNA-down | semi-shared |
| CASK-AS1 | hsa-miR-125a-5p | lncRNA-up_miRNA-down | semi-shared |
| EGOT | hsa-miR-125a-5p | lncRNA-up_miRNA-down | semi-shared |
| MYO16-AS1 | hsa-miR-125a-5p | lncRNA-up_miRNA-down | semi-shared |
| DGCR5 | hsa-miR-125a-5p | lncRNA-up_miRNA-down | semi-shared |
| LINC00525 | hsa-miR-125b-5p | lncRNA-up_miRNA-down | semi-shared |
| C3P1 | hsa-miR-125b-5p | lncRNA-up_miRNA-down | semi-shared |
| DLEU1 | hsa-miR-125b-5p | lncRNA-up_miRNA-down | semi-shared |
| TMEM105 | hsa-miR-125b-5p | lncRNA-up_miRNA-down | semi-shared |
| KRT42P | hsa-miR-125b-5p | lncRNA-up_miRNA-down | semi-shared |
| SLC6A10P | hsa-miR-125b-5p | lncRNA-up_miRNA-down | semi-shared |
| LGALS8-AS1 | hsa-miR-125b-5p | lncRNA-up_miRNA-down | semi-shared |
| NAALADL2-AS2 | hsa-miR-125b-5p | lncRNA-up_miRNA-down | semi-shared |
| LINC00200 | hsa-miR-125b-5p | lncRNA-up_miRNA-down | semi-shared |
| UBE2Q1-AS1 | hsa-miR-125b-5p | lncRNA-up_miRNA-down | semi-shared |
| CASK-AS1 | hsa-miR-125b-5p | lncRNA-up_miRNA-down | semi-shared |
| EGOT | hsa-miR-125b-5p | lncRNA-up_miRNA-down | semi-shared |
| MYO16-AS1 | hsa-miR-125b-5p | lncRNA-up_miRNA-down | semi-shared |
| DGCR5 | hsa-miR-125b-5p | lncRNA-up_miRNA-down | semi-shared |
| DLEU1 | hsa-miR-129-5p | lncRNA-up_miRNA-down | semi-shared |
| EGOT | hsa-miR-129-5p | lncRNA-up_miRNA-down | semi-shared |
| MYO16-AS1 | hsa-miR-129-5p | lncRNA-up_miRNA-down | semi-shared |
| DGCR5 | hsa-miR-129-5p | lncRNA-up_miRNA-down | semi-shared |
| LINC00470 | hsa-miR-129-5p | lncRNA-up_miRNA-down | semi-shared |
| ST20 | hsa-miR-129-5p | lncRNA-up_miRNA-down | semi-shared |
| LINC00221 | hsa-miR-129-5p | lncRNA-up_miRNA-down | semi-shared |
| SNHG12 | hsa-miR-129-5p | lncRNA-up_miRNA-down | semi-shared |
| C10orf55 | hsa-miR-129-5p | lncRNA-up_miRNA-down | semi-shared |
| LINC00393 | hsa-miR-129-5p | lncRNA-up_miRNA-down | semi-shared |
| GK-IT1 | hsa-miR-129-5p | lncRNA-up_miRNA-down | semi-shared |
| ALMS1-IT1 | hsa-miR-129-5p | lncRNA-up_miRNA-down | semi-shared |
| DLEU2 | hsa-miR-129-5p | lncRNA-up_miRNA-down | semi-shared |
| TM4SF19-AS1 | hsa-miR-129-5p | lncRNA-up_miRNA-down | semi-shared |
| LSAMP-AS1 | hsa-miR-129-5p | lncRNA-up_miRNA-down | semi-shared |
| SNHG3 | hsa-miR-129-5p | lncRNA-up_miRNA-down | semi-shared |
| CSAG4 | hsa-miR-129-5p | lncRNA-up_miRNA-down | semi-shared |
| PCAT1 | hsa-miR-129-5p | lncRNA-up_miRNA-down | semi-shared |
| SNHG1 | hsa-miR-129-5p | lncRNA-up_miRNA-down | semi-shared |
| DLEU1 | hsa-miR-363-3p | lncRNA-up_miRNA-down | semi-shared |
| KRT42P | hsa-miR-363-3p | lncRNA-up_miRNA-down | semi-shared |
| SLC6A10P | hsa-miR-363-3p | lncRNA-up_miRNA-down | semi-shared |
| ST20 | hsa-miR-363-3p | lncRNA-up_miRNA-down | semi-shared |
| LINC00221 | hsa-miR-363-3p | lncRNA-up_miRNA-down | semi-shared |
| DLEU2 | hsa-miR-363-3p | lncRNA-up_miRNA-down | semi-shared |
| PCAT1 | hsa-miR-363-3p | lncRNA-up_miRNA-down | semi-shared |
| SNHG1 | hsa-miR-363-3p | lncRNA-up_miRNA-down | semi-shared |
| C2orf27A | hsa-miR-363-3p | lncRNA-up_miRNA-down | semi-shared |
| LINC00392 | hsa-miR-363-3p | lncRNA-up_miRNA-down | semi-shared |
| PRRT3-AS1 | hsa-miR-363-3p | lncRNA-up_miRNA-down | semi-shared |
| EMBP1 | hsa-miR-363-3p | lncRNA-up_miRNA-down | semi-shared |
| C3P1 | hsa-miR-23b-3p | lncRNA-up_miRNA-down | semi-shared |
| DLEU1 | hsa-miR-23b-3p | lncRNA-up_miRNA-down | semi-shared |
| TMEM105 | hsa-miR-23b-3p | lncRNA-up_miRNA-down | semi-shared |
| EGOT | hsa-miR-23b-3p | lncRNA-up_miRNA-down | semi-shared |
| MYO16-AS1 | hsa-miR-23b-3p | lncRNA-up_miRNA-down | semi-shared |
| DGCR5 | hsa-miR-23b-3p | lncRNA-up_miRNA-down | semi-shared |
| DLEU2 | hsa-miR-23b-3p | lncRNA-up_miRNA-down | semi-shared |
| TM4SF19-AS1 | hsa-miR-23b-3p | lncRNA-up_miRNA-down | semi-shared |
| PCAT1 | hsa-miR-23b-3p | lncRNA-up_miRNA-down | semi-shared |
| SNHG1 | hsa-miR-23b-3p | lncRNA-up_miRNA-down | semi-shared |
| C2orf27A | hsa-miR-23b-3p | lncRNA-up_miRNA-down | semi-shared |
| EMBP1 | hsa-miR-23b-3p | lncRNA-up_miRNA-down | semi-shared |
| GBAP1 | hsa-miR-23b-3p | lncRNA-up_miRNA-down | semi-shared |
| STK24-AS1 | hsa-miR-23b-3p | lncRNA-up_miRNA-down | semi-shared |
| GAS5 | hsa-miR-23b-3p | lncRNA-up_miRNA-down | semi-shared |
| LINC00299 | hsa-miR-23b-3p | lncRNA-up_miRNA-down | semi-shared |
| hsa-miR-125a-5p | E2F2 | miRNA-down_mRNA-up | semi-shared |
| hsa-miR-125a-5p | AGTRAP | miRNA-down_mRNA-up | semi-shared |
| hsa-miR-125a-5p | UBTD1 | miRNA-down_mRNA-up | semi-shared |
| hsa-miR-125a-5p | CNNM1 | miRNA-down_mRNA-up | semi-shared |
| hsa-miR-125a-5p | SCD | miRNA-down_mRNA-up | semi-shared |
| hsa-miR-125a-5p | FADS2 | miRNA-down_mRNA-up | semi-shared |
| hsa-miR-125a-5p | KLC2 | miRNA-down_mRNA-up | semi-shared |
| hsa-miR-125a-5p | BCAT1 | miRNA-down_mRNA-up | semi-shared |
| hsa-miR-125a-5p | OAS3 | miRNA-down_mRNA-up | semi-shared |
| hsa-miR-125a-5p | WARS1 | miRNA-down_mRNA-up | semi-shared |
| hsa-miR-125a-5p | GINS3 | miRNA-down_mRNA-up | semi-shared |
| hsa-miR-125a-5p | CBFB | miRNA-down_mRNA-up | semi-shared |
| hsa-miR-125a-5p | DVL2 | miRNA-down_mRNA-up | semi-shared |
| hsa-miR-125a-5p | CACNB1 | miRNA-down_mRNA-up | semi-shared |
| hsa-miR-125a-5p | TSEN54 | miRNA-down_mRNA-up | semi-shared |
| hsa-miR-125a-5p | C18orf54 | miRNA-down_mRNA-up | semi-shared |
| hsa-miR-125a-5p | LSM4 | miRNA-down_mRNA-up | semi-shared |
| hsa-miR-125a-5p | SLC1A5 | miRNA-down_mRNA-up | semi-shared |
| hsa-miR-125a-5p | BBC3 | miRNA-down_mRNA-up | semi-shared |
| hsa-miR-125a-5p | POLR1G | miRNA-down_mRNA-up | semi-shared |
| hsa-miR-125a-5p | BCL2L12 | miRNA-down_mRNA-up | semi-shared |
| hsa-miR-125a-5p | KMT5C | miRNA-down_mRNA-up | semi-shared |
| hsa-miR-125a-5p | HOXD1 | miRNA-down_mRNA-up | semi-shared |
| hsa-miR-125a-5p | GLS | miRNA-down_mRNA-up | semi-shared |
| hsa-miR-125a-5p | SNRPB | miRNA-down_mRNA-up | semi-shared |
| hsa-miR-125a-5p | LIF | miRNA-down_mRNA-up | semi-shared |
| hsa-miR-125a-5p | FOXRED2 | miRNA-down_mRNA-up | semi-shared |
| hsa-miR-125a-5p | NUP210 | miRNA-down_mRNA-up | semi-shared |
| hsa-miR-125a-5p | ATP13A3 | miRNA-down_mRNA-up | semi-shared |
| hsa-miR-125a-5p | PARP14 | miRNA-down_mRNA-up | semi-shared |
| hsa-miR-125a-5p | E2F3 | miRNA-down_mRNA-up | semi-shared |
| hsa-miR-125a-5p | LIMK1 | miRNA-down_mRNA-up | semi-shared |
| hsa-miR-125a-5p | MFHAS1 | miRNA-down_mRNA-up | semi-shared |
| hsa-miR-125a-5p | TNFRSF10B | miRNA-down_mRNA-up | semi-shared |
| hsa-miR-125a-5p | EIF4EBP1 | miRNA-down_mRNA-up | semi-shared |
| hsa-miR-125a-5p | QSOX2 | miRNA-down_mRNA-up | semi-shared |
| hsa-miR-125a-5p | IRAK1 | miRNA-down_mRNA-up | semi-shared |
| hsa-miR-125a-5p | SUV39H1 | miRNA-down_mRNA-up | semi-shared |
| hsa-miR-125a-5p | HMGB3 | miRNA-down_mRNA-up | semi-shared |
| hsa-miR-125b-5p | E2F2 | miRNA-down_mRNA-up | semi-shared |
| hsa-miR-125b-5p | AGTRAP | miRNA-down_mRNA-up | semi-shared |
| hsa-miR-125b-5p | UBTD1 | miRNA-down_mRNA-up | semi-shared |
| hsa-miR-125b-5p | CNNM1 | miRNA-down_mRNA-up | semi-shared |
| hsa-miR-125b-5p | SCD | miRNA-down_mRNA-up | semi-shared |
| hsa-miR-125b-5p | FADS2 | miRNA-down_mRNA-up | semi-shared |
| hsa-miR-125b-5p | KLC2 | miRNA-down_mRNA-up | semi-shared |
| hsa-miR-125b-5p | BCAT1 | miRNA-down_mRNA-up | semi-shared |
| hsa-miR-125b-5p | WARS1 | miRNA-down_mRNA-up | semi-shared |
| hsa-miR-125b-5p | GINS3 | miRNA-down_mRNA-up | semi-shared |
| hsa-miR-125b-5p | CBFB | miRNA-down_mRNA-up | semi-shared |
| hsa-miR-125b-5p | DVL2 | miRNA-down_mRNA-up | semi-shared |
| hsa-miR-125b-5p | CACNB1 | miRNA-down_mRNA-up | semi-shared |
| hsa-miR-125b-5p | TSEN54 | miRNA-down_mRNA-up | semi-shared |
| hsa-miR-125b-5p | C18orf54 | miRNA-down_mRNA-up | semi-shared |
| hsa-miR-125b-5p | LSM4 | miRNA-down_mRNA-up | semi-shared |
| hsa-miR-125b-5p | SLC1A5 | miRNA-down_mRNA-up | semi-shared |
| hsa-miR-125b-5p | BBC3 | miRNA-down_mRNA-up | semi-shared |
| hsa-miR-125b-5p | POLR1G | miRNA-down_mRNA-up | semi-shared |
| hsa-miR-125b-5p | BCL2L12 | miRNA-down_mRNA-up | semi-shared |
| hsa-miR-125b-5p | KMT5C | miRNA-down_mRNA-up | semi-shared |
| hsa-miR-125b-5p | HOXD1 | miRNA-down_mRNA-up | semi-shared |
| hsa-miR-125b-5p | GLS | miRNA-down_mRNA-up | semi-shared |
| hsa-miR-125b-5p | SNRPB | miRNA-down_mRNA-up | semi-shared |
| hsa-miR-125b-5p | LIF | miRNA-down_mRNA-up | semi-shared |
| hsa-miR-125b-5p | FOXRED2 | miRNA-down_mRNA-up | semi-shared |
| hsa-miR-125b-5p | NUP210 | miRNA-down_mRNA-up | semi-shared |
| hsa-miR-125b-5p | ATP13A3 | miRNA-down_mRNA-up | semi-shared |
| hsa-miR-125b-5p | PARP14 | miRNA-down_mRNA-up | semi-shared |
| hsa-miR-125b-5p | E2F3 | miRNA-down_mRNA-up | semi-shared |
| hsa-miR-125b-5p | LIMK1 | miRNA-down_mRNA-up | semi-shared |
| hsa-miR-125b-5p | MFHAS1 | miRNA-down_mRNA-up | semi-shared |
| hsa-miR-125b-5p | TNFRSF10B | miRNA-down_mRNA-up | semi-shared |
| hsa-miR-125b-5p | EIF4EBP1 | miRNA-down_mRNA-up | semi-shared |
| hsa-miR-125b-5p | IRAK1 | miRNA-down_mRNA-up | semi-shared |
| hsa-miR-125b-5p | SUV39H1 | miRNA-down_mRNA-up | semi-shared |
| hsa-miR-125b-5p | HMGB3 | miRNA-down_mRNA-up | semi-shared |
| hsa-miR-125b-5p | SPATA33 | miRNA-down_mRNA-up | semi-shared |
| hsa-miR-129-5p | BCL2L12 | miRNA-down_mRNA-up | semi-shared |
| hsa-miR-129-5p | GLS | miRNA-down_mRNA-up | semi-shared |
| hsa-miR-129-5p | ATP13A3 | miRNA-down_mRNA-up | semi-shared |
| hsa-miR-129-5p | E2F3 | miRNA-down_mRNA-up | semi-shared |
| hsa-miR-129-5p | RGS16 | miRNA-down_mRNA-up | semi-shared |
| hsa-miR-129-5p | DUSP10 | miRNA-down_mRNA-up | semi-shared |
| hsa-miR-129-5p | CHML | miRNA-down_mRNA-up | semi-shared |
| hsa-miR-129-5p | SYT14 | miRNA-down_mRNA-up | semi-shared |
| hsa-miR-129-5p | DDX21 | miRNA-down_mRNA-up | semi-shared |
| hsa-miR-129-5p | PGM2L1 | miRNA-down_mRNA-up | semi-shared |
| hsa-miR-129-5p | CCND1 | miRNA-down_mRNA-up | semi-shared |
| hsa-miR-129-5p | P2RY6 | miRNA-down_mRNA-up | semi-shared |
| hsa-miR-129-5p | POLD3 | miRNA-down_mRNA-up | semi-shared |
| hsa-miR-129-5p | BICD1 | miRNA-down_mRNA-up | semi-shared |
| hsa-miR-129-5p | HSPH1 | miRNA-down_mRNA-up | semi-shared |
| hsa-miR-129-5p | POU4F1 | miRNA-down_mRNA-up | semi-shared |
| hsa-miR-129-5p | TNFSF11 | miRNA-down_mRNA-up | semi-shared |
| hsa-miR-129-5p | UCHL3 | miRNA-down_mRNA-up | semi-shared |
| hsa-miR-129-5p | AJUBA | miRNA-down_mRNA-up | semi-shared |
| hsa-miR-129-5p | CBX4 | miRNA-down_mRNA-up | semi-shared |
| hsa-miR-129-5p | TYMS | miRNA-down_mRNA-up | semi-shared |
| hsa-miR-129-5p | ICAM1 | miRNA-down_mRNA-up | semi-shared |
| hsa-miR-129-5p | STAT1 | miRNA-down_mRNA-up | semi-shared |
| hsa-miR-129-5p | SOX11 | miRNA-down_mRNA-up | semi-shared |
| hsa-miR-129-5p | PMEPA1 | miRNA-down_mRNA-up | semi-shared |
| hsa-miR-129-5p | NOP56 | miRNA-down_mRNA-up | semi-shared |
| hsa-miR-129-5p | TGIF2 | miRNA-down_mRNA-up | semi-shared |
| hsa-miR-129-5p | DONSON | miRNA-down_mRNA-up | semi-shared |
| hsa-miR-129-5p | RUNX1 | miRNA-down_mRNA-up | semi-shared |
| hsa-miR-129-5p | WNT5A | miRNA-down_mRNA-up | semi-shared |
| hsa-miR-129-5p | CLDN1 | miRNA-down_mRNA-up | semi-shared |
| hsa-miR-129-5p | SKP2 | miRNA-down_mRNA-up | semi-shared |
| hsa-miR-129-5p | PCDHGC5 | miRNA-down_mRNA-up | semi-shared |
| hsa-miR-129-5p | PCDHGA8 | miRNA-down_mRNA-up | semi-shared |
| hsa-miR-129-5p | DPY19L1 | miRNA-down_mRNA-up | semi-shared |
| hsa-miR-129-5p | INHBA | miRNA-down_mRNA-up | semi-shared |
| hsa-miR-129-5p | TMEM65 | miRNA-down_mRNA-up | semi-shared |
| hsa-miR-129-5p | OTUD6B | miRNA-down_mRNA-up | semi-shared |
| hsa-miR-129-5p | TMEM67 | miRNA-down_mRNA-up | semi-shared |
| hsa-miR-129-5p | LAPTM4B | miRNA-down_mRNA-up | semi-shared |
| hsa-miR-129-5p | FZD6 | miRNA-down_mRNA-up | semi-shared |
| hsa-miR-129-5p | FAM91A1 | miRNA-down_mRNA-up | semi-shared |
| hsa-miR-129-5p | COL27A1 | miRNA-down_mRNA-up | semi-shared |
| hsa-miR-129-5p | NKRF | miRNA-down_mRNA-up | semi-shared |
| hsa-miR-129-5p | ZNF280C | miRNA-down_mRNA-up | semi-shared |
| hsa-miR-129-5p | DKC1 | miRNA-down_mRNA-up | semi-shared |
| hsa-miR-363-3p | BCAT1 | miRNA-down_mRNA-up | semi-shared |
| hsa-miR-363-3p | CBFB | miRNA-down_mRNA-up | semi-shared |
| hsa-miR-363-3p | HOXD1 | miRNA-down_mRNA-up | semi-shared |
| hsa-miR-363-3p | E2F3 | miRNA-down_mRNA-up | semi-shared |
| hsa-miR-363-3p | MFHAS1 | miRNA-down_mRNA-up | semi-shared |
| hsa-miR-363-3p | TNFRSF10B | miRNA-down_mRNA-up | semi-shared |
| hsa-miR-363-3p | DUSP10 | miRNA-down_mRNA-up | semi-shared |
| hsa-miR-363-3p | SOX11 | miRNA-down_mRNA-up | semi-shared |
| hsa-miR-363-3p | PMEPA1 | miRNA-down_mRNA-up | semi-shared |
| hsa-miR-363-3p | WNT5A | miRNA-down_mRNA-up | semi-shared |
| hsa-miR-363-3p | OTUD6B | miRNA-down_mRNA-up | semi-shared |
| hsa-miR-363-3p | FZD6 | miRNA-down_mRNA-up | semi-shared |
| hsa-miR-363-3p | FAM91A1 | miRNA-down_mRNA-up | semi-shared |
| hsa-miR-363-3p | COL27A1 | miRNA-down_mRNA-up | semi-shared |
| hsa-miR-363-3p | ANP32E | miRNA-down_mRNA-up | semi-shared |
| hsa-miR-363-3p | PDPN | miRNA-down_mRNA-up | semi-shared |
| hsa-miR-363-3p | HOXC8 | miRNA-down_mRNA-up | semi-shared |
| hsa-miR-363-3p | FZD10 | miRNA-down_mRNA-up | semi-shared |
| hsa-miR-363-3p | WDR76 | miRNA-down_mRNA-up | semi-shared |
| hsa-miR-363-3p | NUTF2 | miRNA-down_mRNA-up | semi-shared |
| hsa-miR-363-3p | ZNF469 | miRNA-down_mRNA-up | semi-shared |
| hsa-miR-363-3p | TGIF1 | miRNA-down_mRNA-up | semi-shared |
| hsa-miR-363-3p | ADCY3 | miRNA-down_mRNA-up | semi-shared |
| hsa-miR-363-3p | BCL11A | miRNA-down_mRNA-up | semi-shared |
| hsa-miR-363-3p | WDR43 | miRNA-down_mRNA-up | semi-shared |
| hsa-miR-363-3p | MTHFD2 | miRNA-down_mRNA-up | semi-shared |
| hsa-miR-363-3p | MYO1B | miRNA-down_mRNA-up | semi-shared |
| hsa-miR-363-3p | SNAI1 | miRNA-down_mRNA-up | semi-shared |
| hsa-miR-363-3p | ROBO2 | miRNA-down_mRNA-up | semi-shared |
| hsa-miR-363-3p | PPAT | miRNA-down_mRNA-up | semi-shared |
| hsa-miR-363-3p | C4orf46 | miRNA-down_mRNA-up | semi-shared |
| hsa-miR-363-3p | COL12A1 | miRNA-down_mRNA-up | semi-shared |
| hsa-miR-363-3p | IGFBP3 | miRNA-down_mRNA-up | semi-shared |
| hsa-miR-363-3p | SNX10 | miRNA-down_mRNA-up | semi-shared |
| hsa-miR-363-3p | CCT6A | miRNA-down_mRNA-up | semi-shared |
| hsa-miR-363-3p | CCNE2 | miRNA-down_mRNA-up | semi-shared |
| hsa-miR-363-3p | SQLE | miRNA-down_mRNA-up | semi-shared |
| hsa-miR-363-3p | VMA21 | miRNA-down_mRNA-up | semi-shared |
| hsa-miR-23b-3p | GLS | miRNA-down_mRNA-up | semi-shared |
| hsa-miR-23b-3p | NUP210 | miRNA-down_mRNA-up | semi-shared |
| hsa-miR-23b-3p | MFHAS1 | miRNA-down_mRNA-up | semi-shared |
| hsa-miR-23b-3p | TNFRSF10B | miRNA-down_mRNA-up | semi-shared |
| hsa-miR-23b-3p | QSOX2 | miRNA-down_mRNA-up | semi-shared |
| hsa-miR-23b-3p | CHML | miRNA-down_mRNA-up | semi-shared |
| hsa-miR-23b-3p | CCND1 | miRNA-down_mRNA-up | semi-shared |
| hsa-miR-23b-3p | HSPH1 | miRNA-down_mRNA-up | semi-shared |
| hsa-miR-23b-3p | RUNX1 | miRNA-down_mRNA-up | semi-shared |
| hsa-miR-23b-3p | ZNF280C | miRNA-down_mRNA-up | semi-shared |
| hsa-miR-23b-3p | ZNF469 | miRNA-down_mRNA-up | semi-shared |
| hsa-miR-23b-3p | TGIF1 | miRNA-down_mRNA-up | semi-shared |
| hsa-miR-23b-3p | PPAT | miRNA-down_mRNA-up | semi-shared |
| hsa-miR-23b-3p | IFI6 | miRNA-down_mRNA-up | semi-shared |
| hsa-miR-23b-3p | NDC1 | miRNA-down_mRNA-up | semi-shared |
| hsa-miR-23b-3p | DEPDC1 | miRNA-down_mRNA-up | semi-shared |
| hsa-miR-23b-3p | ZNF697 | miRNA-down_mRNA-up | semi-shared |
| hsa-miR-23b-3p | CCSAP | miRNA-down_mRNA-up | semi-shared |
| hsa-miR-23b-3p | CTPS1 | miRNA-down_mRNA-up | semi-shared |
| hsa-miR-23b-3p | SNRPE | miRNA-down_mRNA-up | semi-shared |
| hsa-miR-23b-3p | CD55 | miRNA-down_mRNA-up | semi-shared |
| hsa-miR-23b-3p | FLVCR1 | miRNA-down_mRNA-up | semi-shared |
| hsa-miR-23b-3p | DCUN1D5 | miRNA-down_mRNA-up | semi-shared |
| hsa-miR-23b-3p | RCN1 | miRNA-down_mRNA-up | semi-shared |
| hsa-miR-23b-3p | INHBE | miRNA-down_mRNA-up | semi-shared |
| hsa-miR-23b-3p | OBI1 | miRNA-down_mRNA-up | semi-shared |
| hsa-miR-23b-3p | LINGO1 | miRNA-down_mRNA-up | semi-shared |
| hsa-miR-23b-3p | PMAIP1 | miRNA-down_mRNA-up | semi-shared |
| hsa-miR-23b-3p | MEX3D | miRNA-down_mRNA-up | semi-shared |
| hsa-miR-23b-3p | CNN2 | miRNA-down_mRNA-up | semi-shared |
| hsa-miR-23b-3p | COL5A2 | miRNA-down_mRNA-up | semi-shared |
| hsa-miR-23b-3p | HSPD1 | miRNA-down_mRNA-up | semi-shared |
| hsa-miR-23b-3p | FMNL2 | miRNA-down_mRNA-up | semi-shared |
| hsa-miR-23b-3p | HOXD10 | miRNA-down_mRNA-up | semi-shared |
| hsa-miR-23b-3p | TOPBP1 | miRNA-down_mRNA-up | semi-shared |
| hsa-miR-23b-3p | TFRC | miRNA-down_mRNA-up | semi-shared |
| hsa-miR-23b-3p | IL1RAP | miRNA-down_mRNA-up | semi-shared |
| hsa-miR-23b-3p | HMGB2 | miRNA-down_mRNA-up | semi-shared |
| hsa-miR-23b-3p | NSD2 | miRNA-down_mRNA-up | semi-shared |
| hsa-miR-23b-3p | F2R | miRNA-down_mRNA-up | semi-shared |
| hsa-miR-23b-3p | LMNB1 | miRNA-down_mRNA-up | semi-shared |
| hsa-miR-23b-3p | GGCT | miRNA-down_mRNA-up | semi-shared |
| hsa-miR-23b-3p | ZNF117 | miRNA-down_mRNA-up | semi-shared |
| hsa-miR-23b-3p | TFPI2 | miRNA-down_mRNA-up | semi-shared |
| hsa-miR-23b-3p | ITGB8 | miRNA-down_mRNA-up | semi-shared |
| hsa-miR-23b-3p | MET | miRNA-down_mRNA-up | semi-shared |
| hsa-miR-23b-3p | SNAI2 | miRNA-down_mRNA-up | semi-shared |
| hsa-miR-23b-3p | ERI1 | miRNA-down_mRNA-up | semi-shared |
| hsa-miR-23b-3p | HAUS6 | miRNA-down_mRNA-up | semi-shared |
| hsa-miR-23b-3p | HS6ST2 | miRNA-down_mRNA-up | semi-shared |
| TPTEP1 | hsa-miR-27a-3p | lncRNA-down_miRNA-up | semi-shared |
| SNHG14 | hsa-miR-27a-3p | lncRNA-down_miRNA-up | semi-shared |
| PCA3 | hsa-miR-27a-3p | lncRNA-down_miRNA-up | semi-shared |
| DNMBP-AS1 | hsa-miR-27a-3p | lncRNA-down_miRNA-up | semi-shared |
| PART1 | hsa-miR-27a-3p | lncRNA-down_miRNA-up | semi-shared |
| WDFY3-AS2 | hsa-miR-27a-3p | lncRNA-down_miRNA-up | semi-shared |
| TMEM72-AS1 | hsa-miR-27a-3p | lncRNA-down_miRNA-up | semi-shared |
| CYP1B1-AS1 | hsa-miR-27a-3p | lncRNA-down_miRNA-up | semi-shared |
| LINC00163 | hsa-miR-27a-3p | lncRNA-down_miRNA-up | semi-shared |
| LIFR-AS1 | hsa-miR-27a-3p | lncRNA-down_miRNA-up | semi-shared |
| LINC00261 | hsa-miR-27a-3p | lncRNA-down_miRNA-up | semi-shared |
| FAM138B | hsa-miR-27a-3p | lncRNA-down_miRNA-up | semi-shared |
| MAGI2-AS3 | hsa-miR-27a-3p | lncRNA-down_miRNA-up | semi-shared |
| LINC00507 | hsa-miR-27a-3p | lncRNA-down_miRNA-up | semi-shared |
| ZNF300P1 | hsa-miR-27a-3p | lncRNA-down_miRNA-up | semi-shared |
| TPTEP1 | hsa-miR-455-5p | lncRNA-down_miRNA-up | semi-shared |
| SNHG14 | hsa-miR-455-5p | lncRNA-down_miRNA-up | semi-shared |
| WDFY3-AS2 | hsa-miR-455-5p | lncRNA-down_miRNA-up | semi-shared |
| MAGI2-AS3 | hsa-miR-455-5p | lncRNA-down_miRNA-up | semi-shared |
| LINC00507 | hsa-miR-455-5p | lncRNA-down_miRNA-up | semi-shared |
| LINC00332 | hsa-miR-455-5p | lncRNA-down_miRNA-up | semi-shared |
| SNHG14 | hsa-miR-590-5p | lncRNA-down_miRNA-up | semi-shared |
| LINC00365 | hsa-miR-590-5p | lncRNA-down_miRNA-up | semi-shared |
| JAZF1-AS1 | hsa-miR-590-5p | lncRNA-down_miRNA-up | semi-shared |
| PART1 | hsa-miR-590-5p | lncRNA-down_miRNA-up | semi-shared |
| WDFY3-AS2 | hsa-miR-590-5p | lncRNA-down_miRNA-up | semi-shared |
| CYP1B1-AS1 | hsa-miR-590-5p | lncRNA-down_miRNA-up | semi-shared |
| hsa-miR-27a-3p | EGR1 | miRNA-up_mRNA-down | semi-shared |
| hsa-miR-27a-3p | ACTA2 | miRNA-up_mRNA-down | semi-shared |
| hsa-miR-27a-3p | CDON | miRNA-up_mRNA-down | semi-shared |
| hsa-miR-27a-3p | NCAM1 | miRNA-up_mRNA-down | semi-shared |
| hsa-miR-27a-3p | EMP1 | miRNA-up_mRNA-down | semi-shared |
| hsa-miR-27a-3p | CAVIN2 | miRNA-up_mRNA-down | semi-shared |
| hsa-miR-27a-3p | NRP2 | miRNA-up_mRNA-down | semi-shared |
| hsa-miR-27a-3p | RCAN1 | miRNA-up_mRNA-down | semi-shared |
| hsa-miR-27a-3p | LPP | miRNA-up_mRNA-down | semi-shared |
| hsa-miR-27a-3p | SMIM14 | miRNA-up_mRNA-down | semi-shared |
| hsa-miR-27a-3p | IL6ST | miRNA-up_mRNA-down | semi-shared |
| hsa-miR-27a-3p | ID4 | miRNA-up_mRNA-down | semi-shared |
| hsa-miR-27a-3p | SLC7A2 | miRNA-up_mRNA-down | semi-shared |
| hsa-miR-27a-3p | PRKAA2 | miRNA-up_mRNA-down | semi-shared |
| hsa-miR-27a-3p | CPEB3 | miRNA-up_mRNA-down | semi-shared |
| hsa-miR-27a-3p | PDE3A | miRNA-up_mRNA-down | semi-shared |
| hsa-miR-27a-3p | KCTD12 | miRNA-up_mRNA-down | semi-shared |
| hsa-miR-27a-3p | GPD1L | miRNA-up_mRNA-down | semi-shared |
| hsa-miR-27a-3p | GCNT2 | miRNA-up_mRNA-down | semi-shared |
| hsa-miR-27a-3p | SLC35F1 | miRNA-up_mRNA-down | semi-shared |
| hsa-miR-27a-3p | RUNX1T1 | miRNA-up_mRNA-down | semi-shared |
| hsa-miR-27a-3p | ALDH4A1 | miRNA-up_mRNA-down | semi-shared |
| hsa-miR-27a-3p | SLC9A1 | miRNA-up_mRNA-down | semi-shared |
| hsa-miR-27a-3p | RIMS3 | miRNA-up_mRNA-down | semi-shared |
| hsa-miR-27a-3p | PLPP3 | miRNA-up_mRNA-down | semi-shared |
| hsa-miR-27a-3p | TXNIP | miRNA-up_mRNA-down | semi-shared |
| hsa-miR-27a-3p | SHE | miRNA-up_mRNA-down | semi-shared |
| hsa-miR-27a-3p | PBXIP1 | miRNA-up_mRNA-down | semi-shared |
| hsa-miR-27a-3p | ROR1 | miRNA-up_mRNA-down | semi-shared |
| hsa-miR-27a-3p | ABCD3 | miRNA-up_mRNA-down | semi-shared |
| hsa-miR-27a-3p | TLCD4 | miRNA-up_mRNA-down | semi-shared |
| hsa-miR-27a-3p | MAGI3 | miRNA-up_mRNA-down | semi-shared |
| hsa-miR-27a-3p | ATP1A2 | miRNA-up_mRNA-down | semi-shared |
| hsa-miR-27a-3p | RGS2 | miRNA-up_mRNA-down | semi-shared |
| hsa-miR-27a-3p | BTG2 | miRNA-up_mRNA-down | semi-shared |
| hsa-miR-27a-3p | C1orf115 | miRNA-up_mRNA-down | semi-shared |
| hsa-miR-27a-3p | ITGA8 | miRNA-up_mRNA-down | semi-shared |
| hsa-miR-27a-3p | ZCCHC24 | miRNA-up_mRNA-down | semi-shared |
| hsa-miR-27a-3p | CELF2 | miRNA-up_mRNA-down | semi-shared |
| hsa-miR-27a-3p | CACNB2 | miRNA-up_mRNA-down | semi-shared |
| hsa-miR-27a-3p | ZEB1 | miRNA-up_mRNA-down | semi-shared |
| hsa-miR-27a-3p | SNCG | miRNA-up_mRNA-down | semi-shared |
| hsa-miR-27a-3p | OPCML | miRNA-up_mRNA-down | semi-shared |
| hsa-miR-27a-3p | AMOTL1 | miRNA-up_mRNA-down | semi-shared |
| hsa-miR-27a-3p | JAM3 | miRNA-up_mRNA-down | semi-shared |
| hsa-miR-27a-3p | METTL7A | miRNA-up_mRNA-down | semi-shared |
| hsa-miR-27a-3p | SMAD9 | miRNA-up_mRNA-down | semi-shared |
| hsa-miR-27a-3p | NOVA1 | miRNA-up_mRNA-down | semi-shared |
| hsa-miR-27a-3p | AKAP6 | miRNA-up_mRNA-down | semi-shared |
| hsa-miR-27a-3p | NPAS3 | miRNA-up_mRNA-down | semi-shared |
| hsa-miR-27a-3p | NRXN3 | miRNA-up_mRNA-down | semi-shared |
| hsa-miR-27a-3p | EML1 | miRNA-up_mRNA-down | semi-shared |
| hsa-miR-27a-3p | MEIS2 | miRNA-up_mRNA-down | semi-shared |
| hsa-miR-27a-3p | RAB27A | miRNA-up_mRNA-down | semi-shared |
| hsa-miR-27a-3p | RBPMS2 | miRNA-up_mRNA-down | semi-shared |
| hsa-miR-27a-3p | TMOD2 | miRNA-up_mRNA-down | semi-shared |
| hsa-miR-27a-3p | PAQR5 | miRNA-up_mRNA-down | semi-shared |
| hsa-miR-27a-3p | ARRDC4 | miRNA-up_mRNA-down | semi-shared |
| hsa-miR-27a-3p | SRL | miRNA-up_mRNA-down | semi-shared |
| hsa-miR-27a-3p | GRIN2A | miRNA-up_mRNA-down | semi-shared |
| hsa-miR-27a-3p | KIAA0513 | miRNA-up_mRNA-down | semi-shared |
| hsa-miR-27a-3p | NTN1 | miRNA-up_mRNA-down | semi-shared |
| hsa-miR-27a-3p | SYT4 | miRNA-up_mRNA-down | semi-shared |
| hsa-miR-27a-3p | CCBE1 | miRNA-up_mRNA-down | semi-shared |
| hsa-miR-27a-3p | MKNK2 | miRNA-up_mRNA-down | semi-shared |
| hsa-miR-27a-3p | PRX | miRNA-up_mRNA-down | semi-shared |
| hsa-miR-27a-3p | KLF2 | miRNA-up_mRNA-down | semi-shared |
| hsa-miR-27a-3p | ZFP36 | miRNA-up_mRNA-down | semi-shared |
| hsa-miR-27a-3p | LONRF2 | miRNA-up_mRNA-down | semi-shared |
| hsa-miR-27a-3p | COBLL1 | miRNA-up_mRNA-down | semi-shared |
| hsa-miR-27a-3p | PDE1A | miRNA-up_mRNA-down | semi-shared |
| hsa-miR-27a-3p | PID1 | miRNA-up_mRNA-down | semi-shared |
| hsa-miR-27a-3p | CCDC85A | miRNA-up_mRNA-down | semi-shared |
| hsa-miR-27a-3p | RIMS4 | miRNA-up_mRNA-down | semi-shared |
| hsa-miR-27a-3p | PCP4 | miRNA-up_mRNA-down | semi-shared |
| hsa-miR-27a-3p | SATB1 | miRNA-up_mRNA-down | semi-shared |
| hsa-miR-27a-3p | THRB | miRNA-up_mRNA-down | semi-shared |
| hsa-miR-27a-3p | CSRNP1 | miRNA-up_mRNA-down | semi-shared |
| hsa-miR-27a-3p | PRICKLE2 | miRNA-up_mRNA-down | semi-shared |
| hsa-miR-27a-3p | ABHD5 | miRNA-up_mRNA-down | semi-shared |
| hsa-miR-27a-3p | ABHD6 | miRNA-up_mRNA-down | semi-shared |
| hsa-miR-27a-3p | ST3GAL6 | miRNA-up_mRNA-down | semi-shared |
| hsa-miR-27a-3p | SPARCL1 | miRNA-up_mRNA-down | semi-shared |
| hsa-miR-27a-3p | PPM1K | miRNA-up_mRNA-down | semi-shared |
| hsa-miR-27a-3p | NAP1L5 | miRNA-up_mRNA-down | semi-shared |
| hsa-miR-27a-3p | SLIT2 | miRNA-up_mRNA-down | semi-shared |
| hsa-miR-27a-3p | PCDH7 | miRNA-up_mRNA-down | semi-shared |
| hsa-miR-27a-3p | PDGFRA | miRNA-up_mRNA-down | semi-shared |
| hsa-miR-27a-3p | STBD1 | miRNA-up_mRNA-down | semi-shared |
| hsa-miR-27a-3p | GAB1 | miRNA-up_mRNA-down | semi-shared |
| hsa-miR-27a-3p | ELL2 | miRNA-up_mRNA-down | semi-shared |
| hsa-miR-27a-3p | PJA2 | miRNA-up_mRNA-down | semi-shared |
| hsa-miR-27a-3p | ISL1 | miRNA-up_mRNA-down | semi-shared |
| hsa-miR-27a-3p | MAP1B | miRNA-up_mRNA-down | semi-shared |
| hsa-miR-27a-3p | PDE8B | miRNA-up_mRNA-down | semi-shared |
| hsa-miR-27a-3p | NR2F1 | miRNA-up_mRNA-down | semi-shared |
| hsa-miR-27a-3p | SOWAHA | miRNA-up_mRNA-down | semi-shared |
| hsa-miR-27a-3p | PPARGC1B | miRNA-up_mRNA-down | semi-shared |
| hsa-miR-27a-3p | SLC22A23 | miRNA-up_mRNA-down | semi-shared |
| hsa-miR-27a-3p | GFOD1 | miRNA-up_mRNA-down | semi-shared |
| hsa-miR-27a-3p | RCAN2 | miRNA-up_mRNA-down | semi-shared |
| hsa-miR-27a-3p | CNR1 | miRNA-up_mRNA-down | semi-shared |
| hsa-miR-27a-3p | CAP2 | miRNA-up_mRNA-down | semi-shared |
| hsa-miR-27a-3p | FUT9 | miRNA-up_mRNA-down | semi-shared |
| hsa-miR-27a-3p | VIP | miRNA-up_mRNA-down | semi-shared |
| hsa-miR-27a-3p | SMOC2 | miRNA-up_mRNA-down | semi-shared |
| hsa-miR-27a-3p | RELN | miRNA-up_mRNA-down | semi-shared |
| hsa-miR-27a-3p | MTURN | miRNA-up_mRNA-down | semi-shared |
| hsa-miR-27a-3p | CALD1 | miRNA-up_mRNA-down | semi-shared |
| hsa-miR-27a-3p | EGR3 | miRNA-up_mRNA-down | semi-shared |
| hsa-miR-27a-3p | MICU3 | miRNA-up_mRNA-down | semi-shared |
| hsa-miR-27a-3p | STMN2 | miRNA-up_mRNA-down | semi-shared |
| hsa-miR-27a-3p | NECAB1 | miRNA-up_mRNA-down | semi-shared |
| hsa-miR-27a-3p | TPM2 | miRNA-up_mRNA-down | semi-shared |
| hsa-miR-27a-3p | FNBP1 | miRNA-up_mRNA-down | semi-shared |
| hsa-miR-27a-3p | ADAMTSL1 | miRNA-up_mRNA-down | semi-shared |
| hsa-miR-27a-3p | DNAJB5 | miRNA-up_mRNA-down | semi-shared |
| hsa-miR-27a-3p | NAP1L3 | miRNA-up_mRNA-down | semi-shared |
| hsa-miR-27a-3p | FGF13 | miRNA-up_mRNA-down | semi-shared |
| hsa-miR-27a-3p | TMEM35A | miRNA-up_mRNA-down | semi-shared |
| hsa-miR-455-5p | PDCD4 | miRNA-up_mRNA-down | semi-shared |
| hsa-miR-455-5p | SLITRK5 | miRNA-up_mRNA-down | semi-shared |
| hsa-miR-455-5p | FOXN3 | miRNA-up_mRNA-down | semi-shared |
| hsa-miR-455-5p | PNRC1 | miRNA-up_mRNA-down | semi-shared |
| hsa-miR-455-5p | SLC7A2 | miRNA-up_mRNA-down | semi-shared |
| hsa-miR-455-5p | KLHL15 | miRNA-up_mRNA-down | semi-shared |
| hsa-miR-455-5p | NEXMIF | miRNA-up_mRNA-down | semi-shared |
| hsa-miR-455-5p | SOX5 | miRNA-up_mRNA-down | semi-shared |
| hsa-miR-455-5p | KCTD12 | miRNA-up_mRNA-down | semi-shared |
| hsa-miR-455-5p | AQP4 | miRNA-up_mRNA-down | semi-shared |
| hsa-miR-455-5p | EPHA5 | miRNA-up_mRNA-down | semi-shared |
| hsa-miR-455-5p | CELF2 | miRNA-up_mRNA-down | semi-shared |
| hsa-miR-455-5p | TMOD2 | miRNA-up_mRNA-down | semi-shared |
| hsa-miR-455-5p | PRICKLE2 | miRNA-up_mRNA-down | semi-shared |
| hsa-miR-455-5p | PDGFRA | miRNA-up_mRNA-down | semi-shared |
| hsa-miR-455-5p | PJA2 | miRNA-up_mRNA-down | semi-shared |
| hsa-miR-455-5p | FGF13 | miRNA-up_mRNA-down | semi-shared |
| hsa-miR-455-5p | TPM1 | miRNA-up_mRNA-down | semi-shared |
| hsa-miR-455-5p | DTNA | miRNA-up_mRNA-down | semi-shared |
| hsa-miR-455-5p | FRZB | miRNA-up_mRNA-down | semi-shared |
| hsa-miR-455-5p | AKAP12 | miRNA-up_mRNA-down | semi-shared |
| hsa-miR-455-5p | CASQ2 | miRNA-up_mRNA-down | semi-shared |
| hsa-miR-455-5p | S1PR1 | miRNA-up_mRNA-down | semi-shared |
| hsa-miR-455-5p | HCFC2 | miRNA-up_mRNA-down | semi-shared |
| hsa-miR-455-5p | DSTN | miRNA-up_mRNA-down | semi-shared |
| hsa-miR-455-5p | ACOX3 | miRNA-up_mRNA-down | semi-shared |
| hsa-miR-455-5p | ENPP6 | miRNA-up_mRNA-down | semi-shared |
| hsa-miR-455-5p | SNX24 | miRNA-up_mRNA-down | semi-shared |
| hsa-miR-455-5p | FILIP1 | miRNA-up_mRNA-down | semi-shared |
| hsa-miR-455-5p | JAZF1 | miRNA-up_mRNA-down | semi-shared |
| hsa-miR-455-5p | FLNC | miRNA-up_mRNA-down | semi-shared |
| hsa-miR-455-5p | TEK | miRNA-up_mRNA-down | semi-shared |
| hsa-miR-455-5p | RECK | miRNA-up_mRNA-down | semi-shared |
| hsa-miR-455-5p | PALM2AKAP2 | miRNA-up_mRNA-down | semi-shared |
| hsa-miR-455-5p | RAI2 | miRNA-up_mRNA-down | semi-shared |
| hsa-miR-455-5p | MAP7D2 | miRNA-up_mRNA-down | semi-shared |
| hsa-miR-590-5p | ATP2B4 | miRNA-up_mRNA-down | semi-shared |
| hsa-miR-590-5p | PDCD4 | miRNA-up_mRNA-down | semi-shared |
| hsa-miR-590-5p | SLC2A13 | miRNA-up_mRNA-down | semi-shared |
| hsa-miR-590-5p | THBS1 | miRNA-up_mRNA-down | semi-shared |
| hsa-miR-590-5p | ITGB3 | miRNA-up_mRNA-down | semi-shared |
| hsa-miR-590-5p | CAVIN2 | miRNA-up_mRNA-down | semi-shared |
| hsa-miR-590-5p | TIMP3 | miRNA-up_mRNA-down | semi-shared |
| hsa-miR-590-5p | SMIM14 | miRNA-up_mRNA-down | semi-shared |
| hsa-miR-590-5p | DLC1 | miRNA-up_mRNA-down | semi-shared |
| hsa-miR-590-5p | KLHL15 | miRNA-up_mRNA-down | semi-shared |
| hsa-miR-590-5p | CPEB3 | miRNA-up_mRNA-down | semi-shared |
| hsa-miR-590-5p | SOX5 | miRNA-up_mRNA-down | semi-shared |
| hsa-miR-590-5p | AQP4 | miRNA-up_mRNA-down | semi-shared |
| hsa-miR-590-5p | SEZ6L | miRNA-up_mRNA-down | semi-shared |
| hsa-miR-590-5p | TENT5A | miRNA-up_mRNA-down | semi-shared |
| hsa-miR-590-5p | GCNT2 | miRNA-up_mRNA-down | semi-shared |
| hsa-miR-590-5p | ABCD3 | miRNA-up_mRNA-down | semi-shared |
| hsa-miR-590-5p | BTG2 | miRNA-up_mRNA-down | semi-shared |
| hsa-miR-590-5p | SATB1 | miRNA-up_mRNA-down | semi-shared |
| hsa-miR-590-5p | ST3GAL6 | miRNA-up_mRNA-down | semi-shared |
| hsa-miR-590-5p | NAP1L5 | miRNA-up_mRNA-down | semi-shared |
| hsa-miR-590-5p | PJA2 | miRNA-up_mRNA-down | semi-shared |
| hsa-miR-590-5p | CALD1 | miRNA-up_mRNA-down | semi-shared |
| hsa-miR-590-5p | EGR3 | miRNA-up_mRNA-down | semi-shared |
| hsa-miR-590-5p | NAP1L3 | miRNA-up_mRNA-down | semi-shared |
| hsa-miR-590-5p | LEPR | miRNA-up_mRNA-down | semi-shared |
| hsa-miR-590-5p | MYT1L | miRNA-up_mRNA-down | semi-shared |
| hsa-miR-590-5p | BTBD3 | miRNA-up_mRNA-down | semi-shared |
| hsa-miR-590-5p | BVES | miRNA-up_mRNA-down | semi-shared |
| hsa-miR-590-5p | AKAP12 | miRNA-up_mRNA-down | semi-shared |
| hsa-miR-590-5p | RECK | miRNA-up_mRNA-down | semi-shared |
| hsa-miR-590-5p | PALM2AKAP2 | miRNA-up_mRNA-down | semi-shared |
| hsa-miR-590-5p | YOD1 | miRNA-up_mRNA-down | semi-shared |
| hsa-miR-590-5p | FAM107B | miRNA-up_mRNA-down | semi-shared |
| hsa-miR-590-5p | GRIA4 | miRNA-up_mRNA-down | semi-shared |
| hsa-miR-590-5p | ABAT | miRNA-up_mRNA-down | semi-shared |
| hsa-miR-590-5p | TNS1 | miRNA-up_mRNA-down | semi-shared |
| hsa-miR-590-5p | RHOB | miRNA-up_mRNA-down | semi-shared |
| hsa-miR-590-5p | CSTB | miRNA-up_mRNA-down | semi-shared |
| hsa-miR-590-5p | WWTR1 | miRNA-up_mRNA-down | semi-shared |
| hsa-miR-590-5p | ZBTB47 | miRNA-up_mRNA-down | semi-shared |
| hsa-miR-590-5p | SLMAP | miRNA-up_mRNA-down | semi-shared |
| hsa-miR-590-5p | ZSWIM6 | miRNA-up_mRNA-down | semi-shared |
| hsa-miR-590-5p | ECI2 | miRNA-up_mRNA-down | semi-shared |
| hsa-miR-590-5p | FKBP5 | miRNA-up_mRNA-down | semi-shared |
| hsa-miR-590-5p | PLN | miRNA-up_mRNA-down | semi-shared |
| hsa-miR-590-5p | SASH1 | miRNA-up_mRNA-down | semi-shared |
| hsa-miR-590-5p | CNTFR | miRNA-up_mRNA-down | semi-shared |
| hsa-miR-590-5p | ARMCX1 | miRNA-up_mRNA-down | semi-shared |
| hsa-miR-590-5p | GPRASP2 | miRNA-up_mRNA-down | semi-shared |

**Supplementary Figures:**


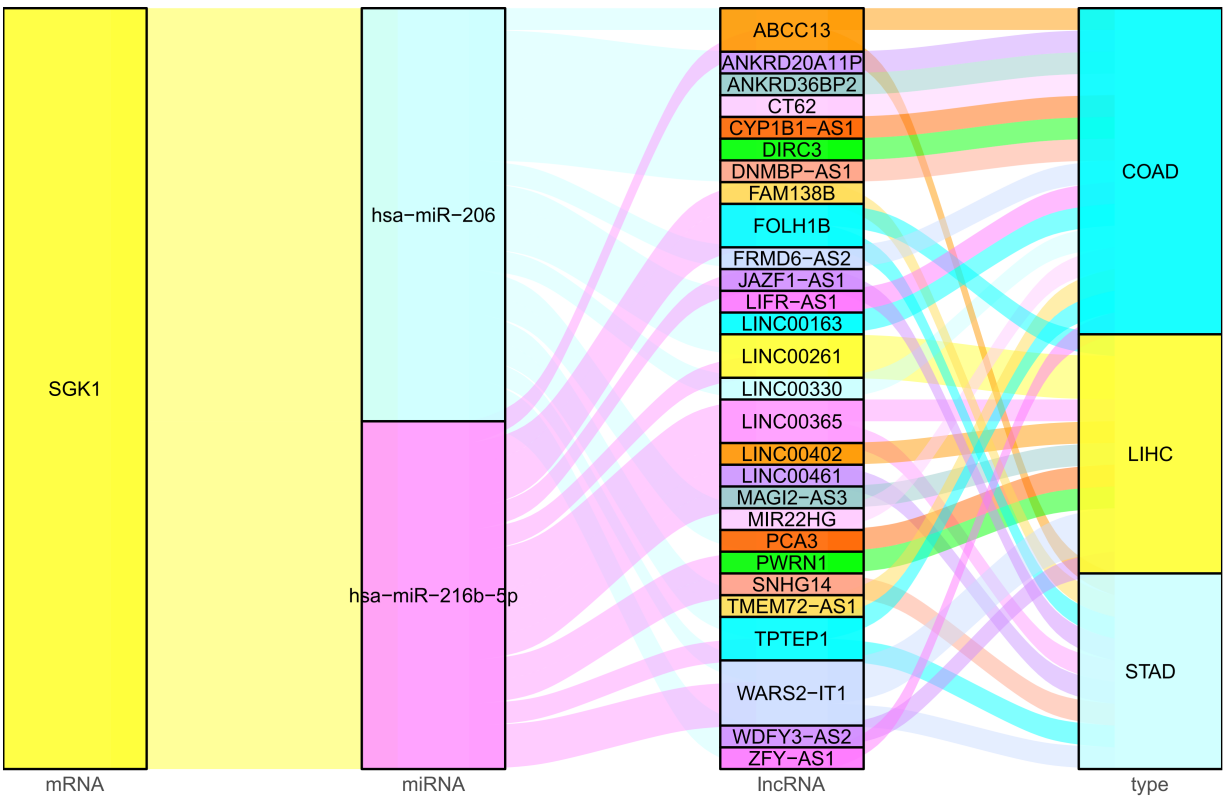


**Figure S1**. SGK1-related lncRNA-miRNA-mRNA network.


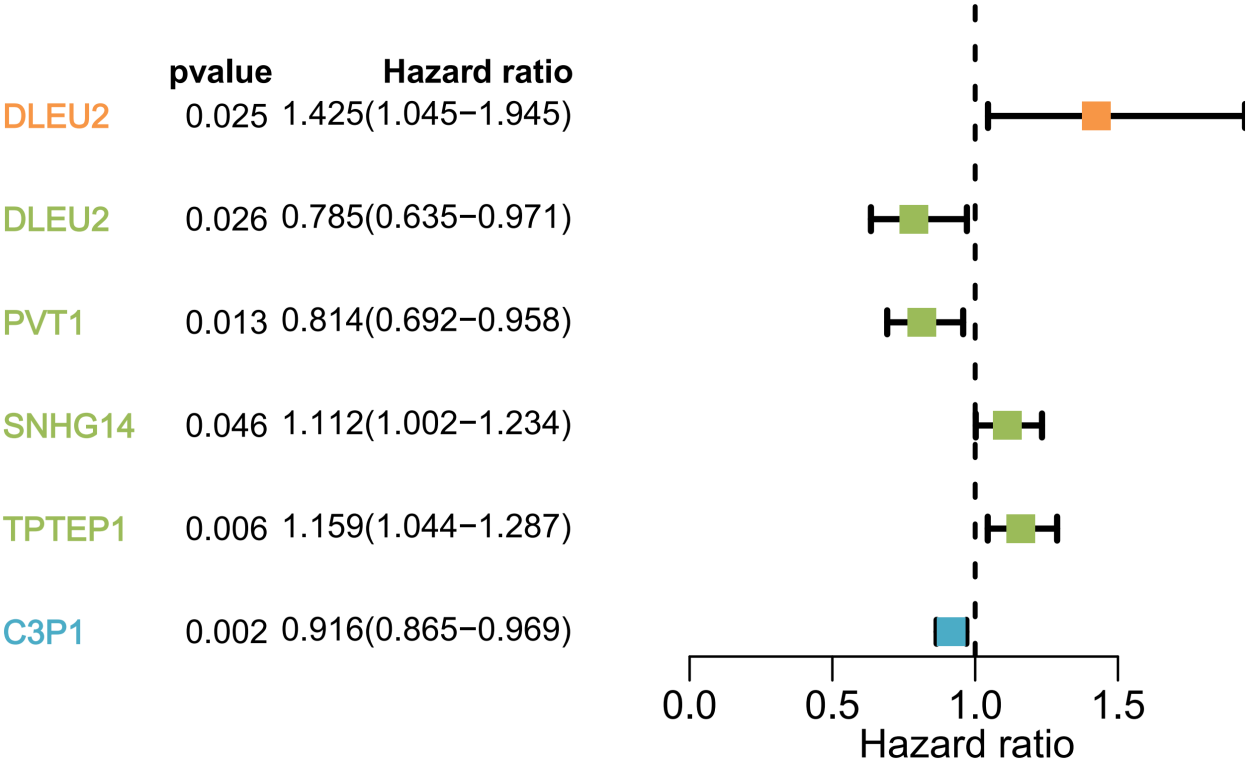


**Figure S2**. Forest plot visualization of the key prognostic lncRNAs.


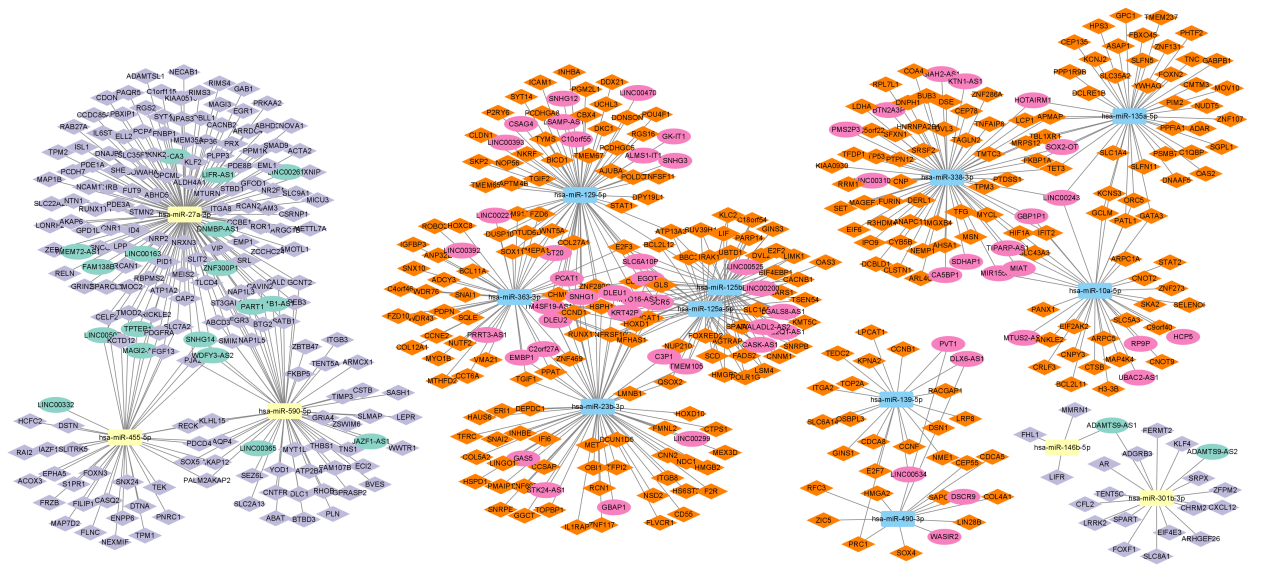


**Figure S3.** ESCA related-ceRNA network nodes (after threshold adjustment).
